# An Adaptive Cholic Acid Dimer for Selective Encapsulation

**DOI:** 10.3390/ijms27114765

**Published:** 2026-05-25

**Authors:** Magdalena-Cristina Stanciu, Gabriela-Liliana Ailiesei, Mirela-Fernanda Zaltariov, Corneliu Cojocaru, Carmen Gherasim, Sofia-Maria Ciocan, Marcela Mihai

**Affiliations:** 1 “Petru Poni” Institute of Macromolecular Chemistry, 41A, Grigore Ghica Voda Alley, 700487 Iasi, Romania; gdarvaru@icmpp.ro (G.-L.A.); zaltariov.mirela@icmpp.ro (M.-F.Z.); cojocaru.corneliu@icmpp.ro (C.C.); gherasim.carmen@icmpp.ro (C.G.); marcela.mihai@icmpp.ro (M.M.); 2 “National College”, 4, Arcu Street, 700125 Iasi, Romania; ciocansofiamaria@gmail.com

**Keywords:** steroidal dimer, click chemistry, inclusion complex, in silico studies

## Abstract

A novel cleft-type cholic acid dimer was synthesized from native bile acid through a six-step reaction sequence. Structural characterization was performed using FTIR, 1D NMR (^1^H, ^13^C and DEPT135), 2D NMR (^1^H,^1^H COSY; ^1^H,^13^C HSQC; ^1^H,^13^C HMBC) and ESI-MS (+). The dimer could entrap both polar and nonpolar guests within its invertible pockets, adapting to changes in solvent polarity. Specifically, UV–Vis absorption and in silico simulations revealed a moderately stable 1:1 host–guest complex for the dimer with Cresol Red sodium salt in an organic solvent. The docked dimer–pyrene complex, investigated by molecular modeling studies, demonstrated an identical 1:1 binding ratio and moderate stability in the micromolar concentration range. Steady-state fluorescence investigations implied that the quenching of pyrene emission by the dimer in an aqueous environment was chiefly a collisional or dynamic pathway rather than the generation of a ground-state dimer–pyrene assembly.

## 1. Introduction

Bile acids (BAs), also known as cholanic acids, are a group of natural compounds playing essential tasks in the digestion and absorption of dietary fats and fat-soluble vitamins, as well as acting as important chemical messengers in metabolism [1,2,3,4]. Primary BAs, cholic acid (CA) and chenodeoxycholic acid (CDCA), are synthesized from cholesterol in the liver, while secondary BAs, deoxycholic acid (DCA), lithocholic acid (LCA), and ursodeoxycholic acid, are formed in the colon through dehydroxylation of primary BAs by the gut microbiota. BAs have a rigid steroidal nucleus composed of four fused rings and a side chain that terminates in a carboxylic acid group. The applications of BAs are mainly based on their facial amphiphilicity, dictated by the occurrence of a hydrophilic face (α-face) with one to three hydroxyl groups (at positions C3, C7, C12), a carboxylic acid tail (C24), and a hydrophobic face (β-face) composed of the methyl groups (C18H3, C19H3, C21H3) and a stiff curved steroidal skeleton [5,6]. BAs were commonly used as versatile small molecules in biomedical applications, being studied as excipients and carriers in pharmaceutical formulations to improve the bioavailability of various drugs [7,8,9,10,11]. BAs were also used as building blocks in supramolecular chemistry, contributing to the construction of new molecular architectures, in which they were hosts, such as cyclic structures (cholaphanes [7,8,12,13,14], cyclocholates [7,8,13,14], cyclophanes [7,8,13,14,15], bile acid-based molecular boxes [8,13,14], crown ethers [8,13,16], cryptands [8,13,14]) or acyclic structures like cleft [8,13,14] and tweezers molecules [8,13,14], or bile acid-based chiral dendrons [8,13,14]. The above-mentioned chemical structures could selectively bind different guests, such as ions [17] or nonpolar/polar organic molecules [18], forming host–guest complexes, also known as inclusion compounds. These complexes were formed through host–guest weak interactions, such as hydrogen bonding, van der Waals forces, and hydrophobic interactions. Organic guest molecules of appropriate size and shape (molecular complementarity) can be physically trapped within the molecular pockets of the host–guest complex. The molecular cavities of the BAs complexes are adaptable because steroids can rearrange their internal medium (from hydrophobic to hydrophilic, or vice versa) in response to changes in solvent polarity (the solvophobic effect). A special class of receptors comprises oligocholate-based foldamers, which exhibit high binding affinity for specific guest molecules due to their ability to fold into helical structures, enabling selective encapsulation [19,20,21,22]. Bile acid host–guest chemistry has applications in chiral separation [23,24], drug delivery systems [25,26,27], and hydrogelators [28,29]. The host–guest complexes based on BAs were also studied using molecular modeling studies, which clarified the molecular recognition mechanism and the structural stability of the inclusion compounds by focusing on host–guest stoichiometry and binding affinity, geometric complementarity, and stabilizing forces. Cyclodextrins and calixarenes are well-known as classic supramolecular hosts, possessing rigid hydrophobic cavities for molecular encapsulation that do not adapt to solvent polarity [30,31]. Flexible hosts based on BAs, able to restructure their steroidal backbone to embed hydrophobic probes in water and hydrophilic guests in nonpolar solvents, were also described [18,32,33]. Some of these steroidal hosts were obtained via a click reaction, which enabled linking of bile acid units in head-to-head or head-to-tail orientations by functionalizing the steroidal nucleus with azide and terminal alkyne groups [18,32,33]. The click reaction is regiospecific, yielding 1,4-disubstituted 1,2,3-triazoles in high yields when Cu(I) catalyst is used.

The current study reported the synthesis of a new tail-to-head dimer based on CA in which two bile acid moieties bearing azide and terminal alkyne functionalities were coupled via a Cu(I)-catalyzed click reaction. The characterization of pure cholanic acid and its derivatives was achieved using FTIR, 1D NMR (^1^H, ^13^C, and DEPT135), 2D NMR (^1^H,^1^H COSY; ^1^H,^13^C HSQC; ^1^H,^13^C HMBC), and ESI-MS (+). This is the first report of a complete 2D NMR assignment for H and C atoms of CA in DMSO-d_6_. The primary objective of this paper was to investigate the inclusion properties of the 1,2,3-triazole-linked product. UV–Vis and steady-state fluorescence spectroscopy were used to demonstrate the sequestration of ionic and hydrophobic guests within the invertible amphiphilic pockets of the steroidal dimer, as well as to determine the stoichiometry and stability of the inclusion complexes. Furthermore, molecular modeling studies were performed to reconcile the experimental findings, enabling analysis of the host–guest complex dynamics and geometry.

## 2. Results and Discussion

### 2.1. Synthesis and Chemical Structure Characterization

**Scheme 1:**
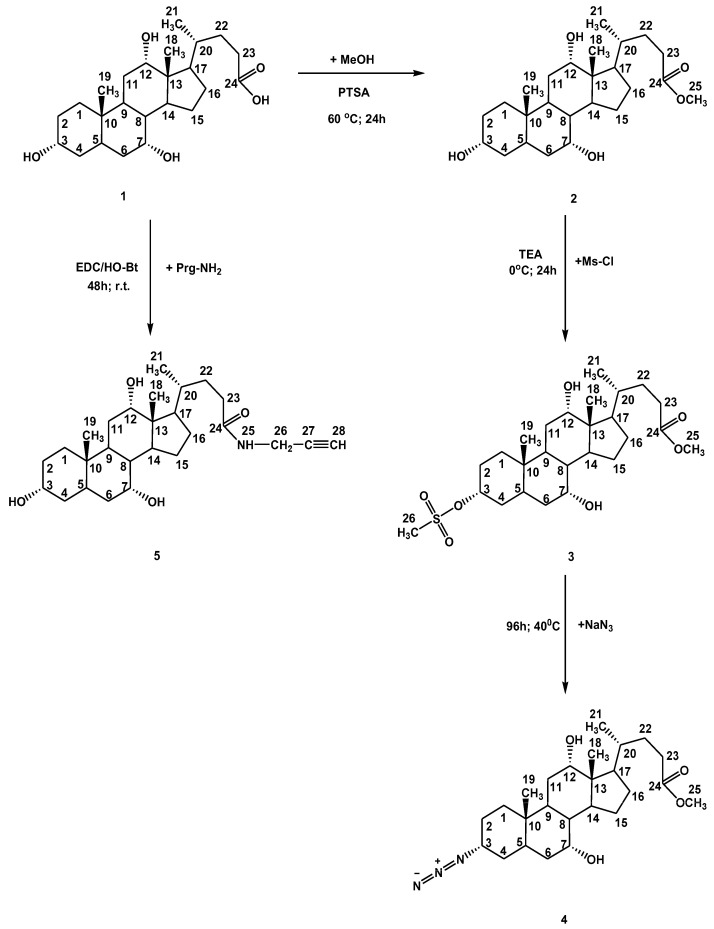
Synthetic route for compounds **1**–**5**.

**Scheme 2:**
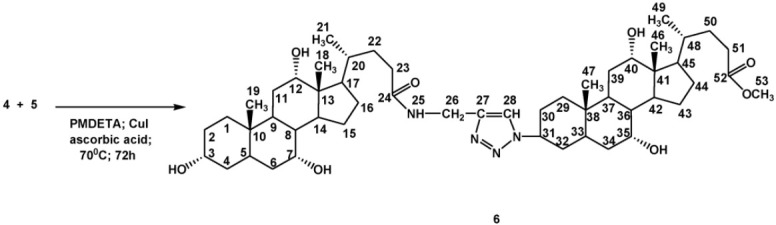
Synthetic path for compound **6**.

The synthesis of the steroidal dimer was carried out in six steps, as illustrated in Scheme 1 and Scheme 2. Since steroidal acid (**1**) is the starting compound for dimer (**6**) synthesis, and a complete 2D NMR investigation in DMSO-d_6_ has not yet been reported in the literature, the values of all its ^1^H and ^13^C signals were presented and discussed below. Previous NMR studies on pristine CA revealed complete ^1^H and ^13^C chemical shift assignments in D_2_O and CD_3_OD as deutered solvents [34,35,36,37]. Other works reported structural elucidation of CA solely through ^1^H NMR spectroscopy in CDCl_3_ and DMSO-d_6_ [34,35,36].

For a better comparison between the data reported in this article and those existing in the literature (highlighted in blue), Table 1 is introduced, containing the ^1^H and ^13^C chemical shifts for the studied compounds.

**Figure 1:**
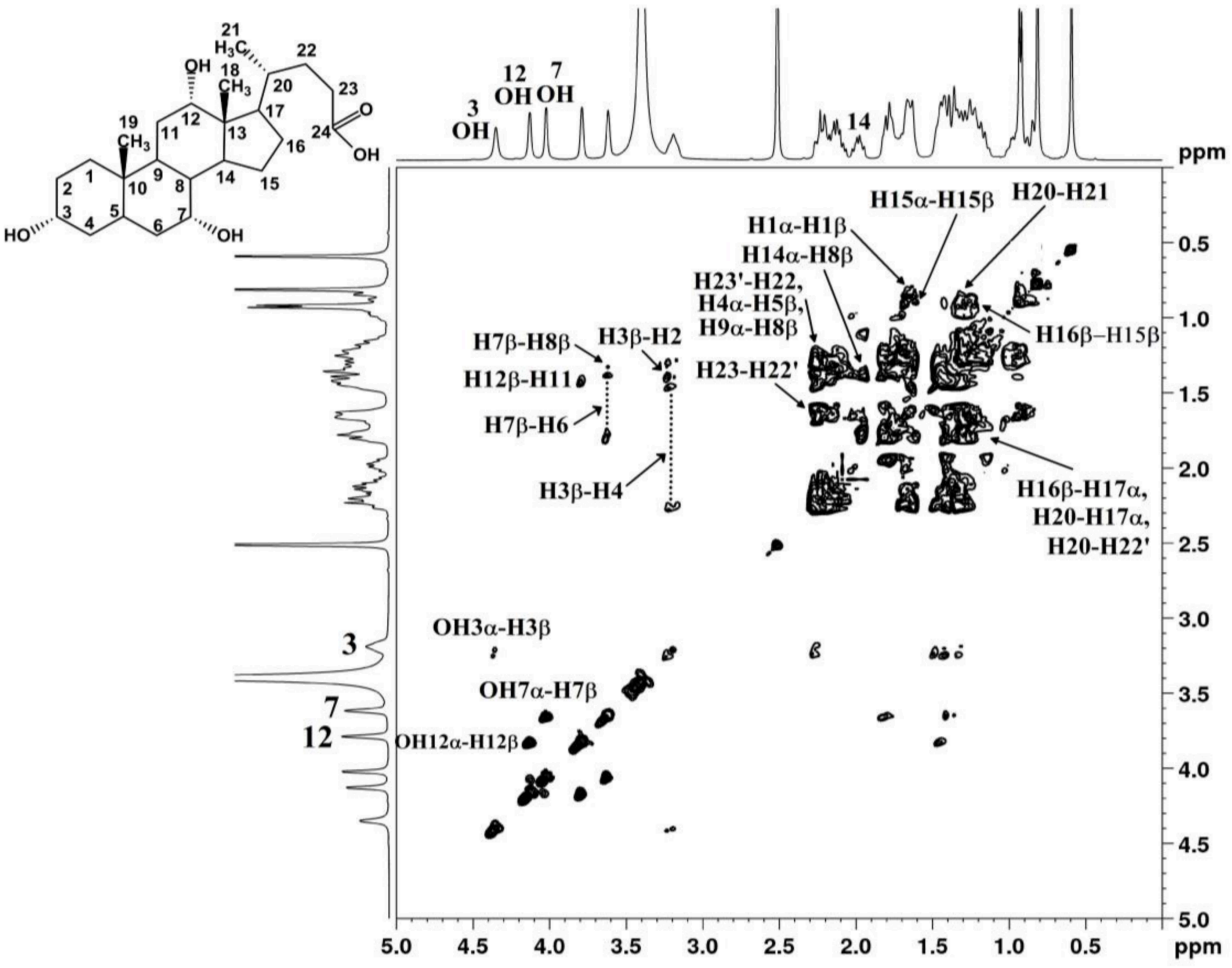
The ^1^H, ^1^H COSY spectrum of CA.

**Figure 2:**
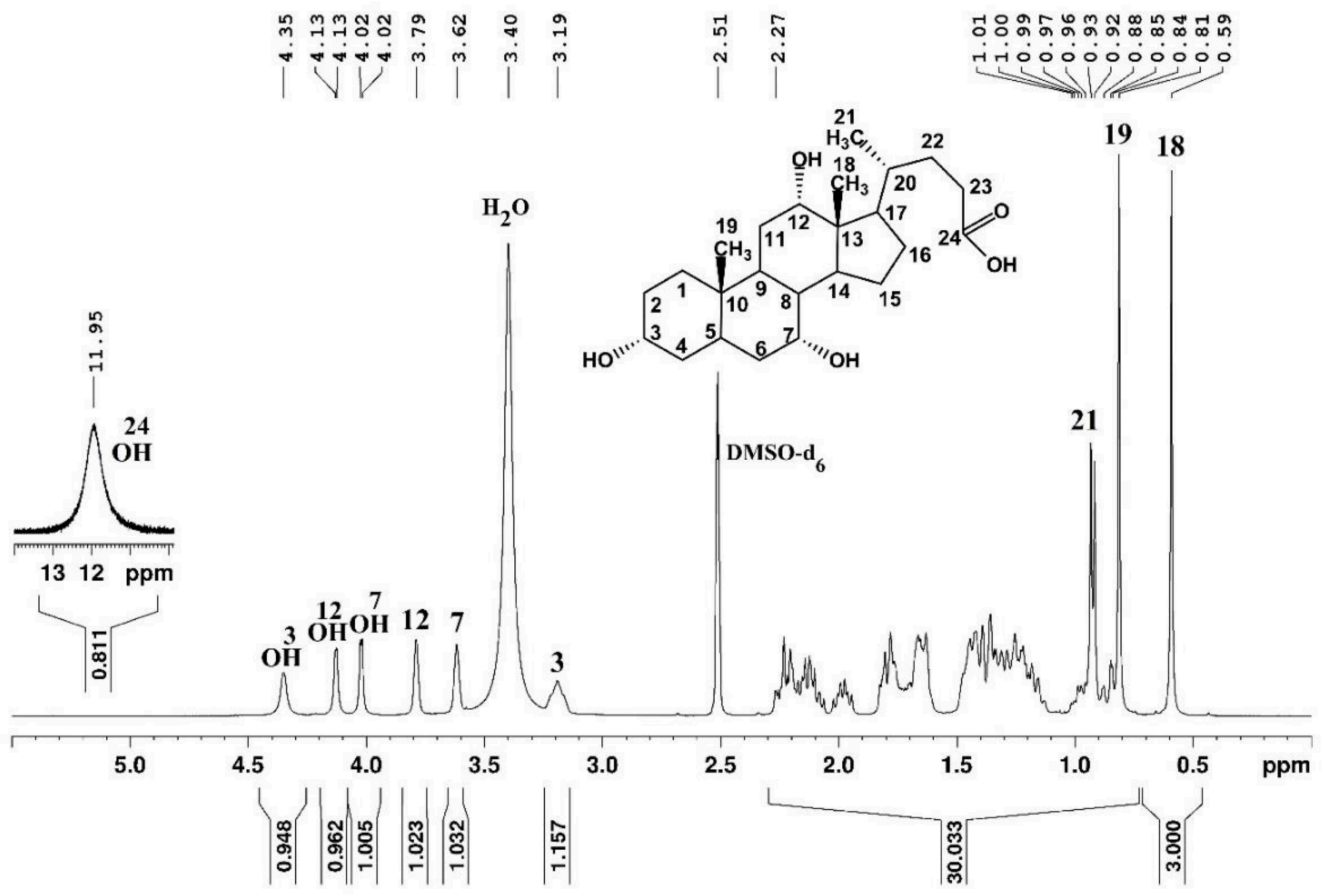
The ^1^H NMR spectrum of CA.

**Figure 3:**
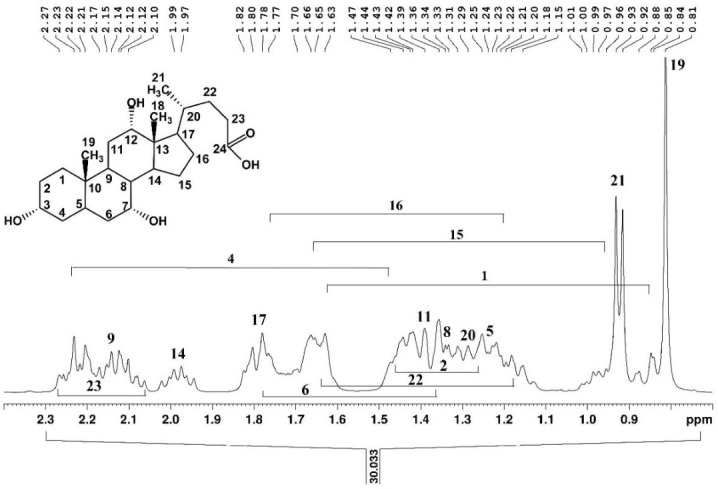
Detailed 0.5-2.4 ppm region in the ^1^H NMR spectrum of CA.

**Figure 4:**
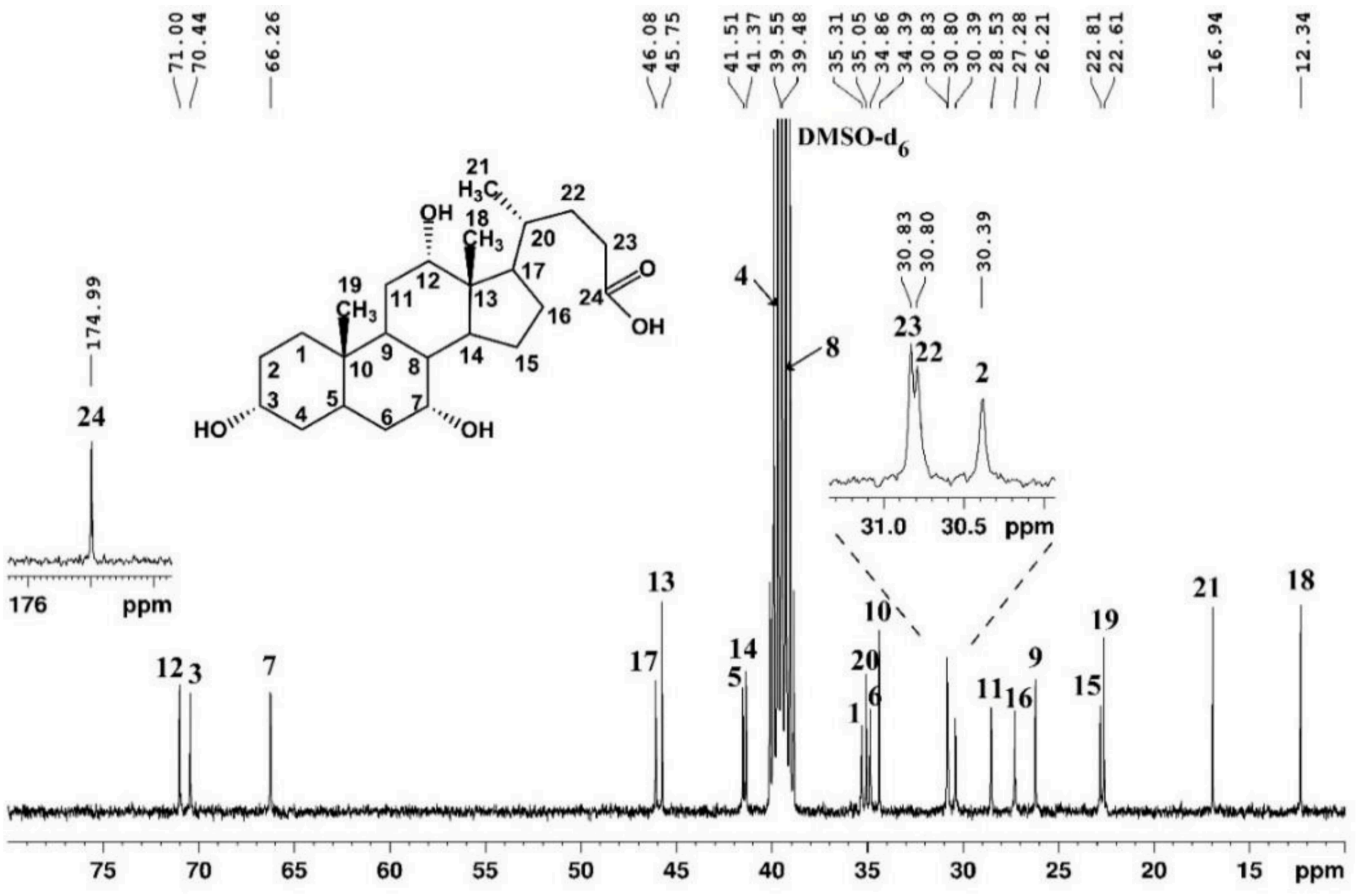
The ^13^C NMR spectrum of CA.

The characterization of CA was performed using 1D and 2D NMR (^1^H,^1^H COSY- in Figure 1; ^1^H- in Figure 2 and Figure 3; ^13^C- in Figure 4; enlarged view of ^1^H,^1^H COSY in Figure S1; DEPT135- in Figure S2; ^1^H,^13^C HSQC-in Figure S3; ^1^H,^13^C HMBC-in Figure S4), FTIR (Figures S5a and S6a) and ESI-MS (+) techniques (Figure S7a). In the chemical structure of CA (Scheme 1), substituents oriented below the plane of the steroidal nucleus are referred to as α (represented by dashed bonds), while those located above the plane are denoted as β (represented by solid bonds). Each methylene group of the steroidal skeleton (at positions C1, C2, C4, C6, C15, and C16) contains two geminal hydrogen atoms, which are spatially non-equivalent (diastereotopic), with one occupying the α-face and the other the β-face of the steroidal nucleus. Consequently, they exhibited distinct chemical shifts in the ^1^H NMR spectrum (Figure 2 and Figure 3). For easier visualization of the attributions, the α and β notations were used only for the COSY spectrum (Figure 1). The methylene groups at positions C22 and C23 of the CA side chain also have two geminal hydrogens. Since these protons did not adopt the α/β facial orientations, they were identified as H22/H22′ and H23/H23′, respectively, to distinguish them. The methyl group signals were assigned based on their chemical shifts and multiplicities: H18 and H19 appeared as singlets (0.59 and 0.81 ppm), while H21 was identified as a doublet (0.91–0.93 ppm). The geminal protons of the three steroidal hydroxyl groups were found downfield between 3.19 and 3.78 ppm, and the terminal carboxylic proton (C24OOH) was clearly identified at 11.95 ppm (Table 1). To distinguish between the peaks corresponding to OH groups in the ^1^H NMR spectrum, the COSY spectrum (Figure 1) was used, with the mention that some notations were simplified by using vertical dots to highlight cross correlations between vicinal CH α/β and geminal CH_2_ α/β. For a better view, an enlarged image of the 0.8–2.4 ppm region of the COSY spectrum is provided in Figure S1. The hydroxyl groups (OH3α, OH12α, and OH7α) were validated by specific cross-peaks arising from J-coupling to their respective neighboring protons. Similarly, the identification of H20 and H8β was made possible by key correlations (H20-H21 and H14α-H8β) (Figure 1 and Figure S1). The assignment of proton signals was performed using COSY correlations, starting with the attributions of H20 (1.29 ppm) and H8β (1.34 ppm) via their cross-peaks with H21 and H14α. The signals for H1α (1.63 ppm) and H1β (0.84 ppm), along with the stereospecific H15α (1.66 ppm) and H15β (0.95 ppm), were similarly identified. Using H15β as a reference, a sequential mapping revealed the shifts for H16β (1.19 ppm), H17α (1.78 ppm), H22’/H22 (1.64/1.18 ppm), and H23’ (2.27 ppm). Additionally, correlations with H3β and H7β allowed for the assignment of methylene protons H4α/β (2.22/1.46 ppm), H6α/β (1.80/1.36 ppm), and H2α/β (1.26/1.46 ppm). A previous study was consulted to differentiate between the α and β geminal protons of the methylene groups (C1, C2, C4, C6, C15, C16, C22, and C23) of CA and its derivatives, as the 2D NMR techniques employed in this work (COSY, HMBC, and HSQC) did not allow for such a distinction [38].

The vast majority of proton steroidal signals (0.84–2.27 ppm) were too overlapped to be identified individually only from the COSY spectrum, the full identification of the signals being achieved using the ^1^H,^13^C HSQC spectrum (Figure S3), which revealed all direct bond H-C correlations (represented by horizontal dots). HSQC analysis revealed that the C11 methylene protons (α and β) are isochronous (1.39 ppm) due to their identical chemical environment. The DEPT135 spectrum of CA (Figure S2) [39] enabled the precise assignment of overlapping and closely spaced signals, helping in the assignment of all ^13^C signals (Figure 4). Specifically, it allowed the identification of C4 (39.55 ppm) and C8 (39.47 ppm), which were overlapped by the residual DMSO-d6 peak, and differentiated various methylene, methine, and methyl carbons, namely C1 (35.30 ppm), C5 (41.51 ppm), C6 (34.86 ppm), C14 (41.36 ppm), C15 (22.80 ppm), C19 (22.61 ppm), and C20 (35.05 ppm). Furthermore, the absence of signals in the DEPT135 spectrum confirmed the quaternary nature of C10 (34.39 ppm) and C13 (45.75 ppm) and allowed their assignment. All ^1^H and ^13^C assignments were confirmed by the HMBC data (Figure S4), which showed multiple-bond correlations (2*J* and 3*J*). Two-bond correlations (2*J*) such as H14–C15, H23–C22, and H21–C20, as well as three-bond correlations (3*J*) like H8–C6, H15–C17, and H22′–C24, could be seen in the spectrum. These findings were in good agreement with previous bile acids’ NMR studies [34]. The FTIR spectrum of the pristine CA (Figure S5a) was consistent with earlier works [1,40,41]. Due to the formation of hydrogen bonds with varying strengths, the FTIR spectra of compounds 1–6 displayed a broad, unresolved absorption envelope in the 3800–3080 cm^−1^ region (Figure S6). To address this, a curve-fitting deconvolution method was applied, which successfully resolved the broad band of compound 1 into distinct sub-bands (Figure S6a). These discrete signals, ranging from 3550 to 3170 cm^−1^, were attributed to the different steric environments and spatial orientations of the hydroxyl groups at C3, C7, C12, and C24. Specifically, bands above 3300 cm^−1^ were assigned to free and intermolecularly H-bonded OH stretching from the ring hydroxyls, while those below 3300 cm^−1^ characterized the carboxylic OH (C24) involved in strong dimeric or polymeric interactions [42,43].

The FTIR analysis (Figure S5a) confirmed the steroidal framework through several diagnostic signals: the C=O stretching at 1716 cm^−1^, and various aliphatic CH_2_ and CH_3_ vibrations, including asymmetric/symmetric stretching (2966–2872 cm^−1^), CH_2_ scissoring (1468 cm^−1^), and the characteristic methyl “umbrella” deformation (1375 cm^−1^). Furthermore, the fingerprint region (1300–1000 cm^−1^) enabled precise assignment of C–O stretching vibrations associated with the OH groups at C3, C7, and C12. The characterization was completed by identifying in-plane and out-of-plane OH bending vibrations, as well as skeletal vibrations of the steroidal ring system appearing below 1000 cm^−1^. The ESI-MS (+) spectrum of compound **1** (Figure S7a) exhibited peaks at *m*/*z* 431.2506 ([M + Na]^+^), 817.4658 ([2M + H]^+^), and 839.4621 ([2M + Na]^+^), consistent with the molecular weight of the protonated bile acid and its corresponding protonated and sodiated dimeric forms, respectively.

**Figure 5:**
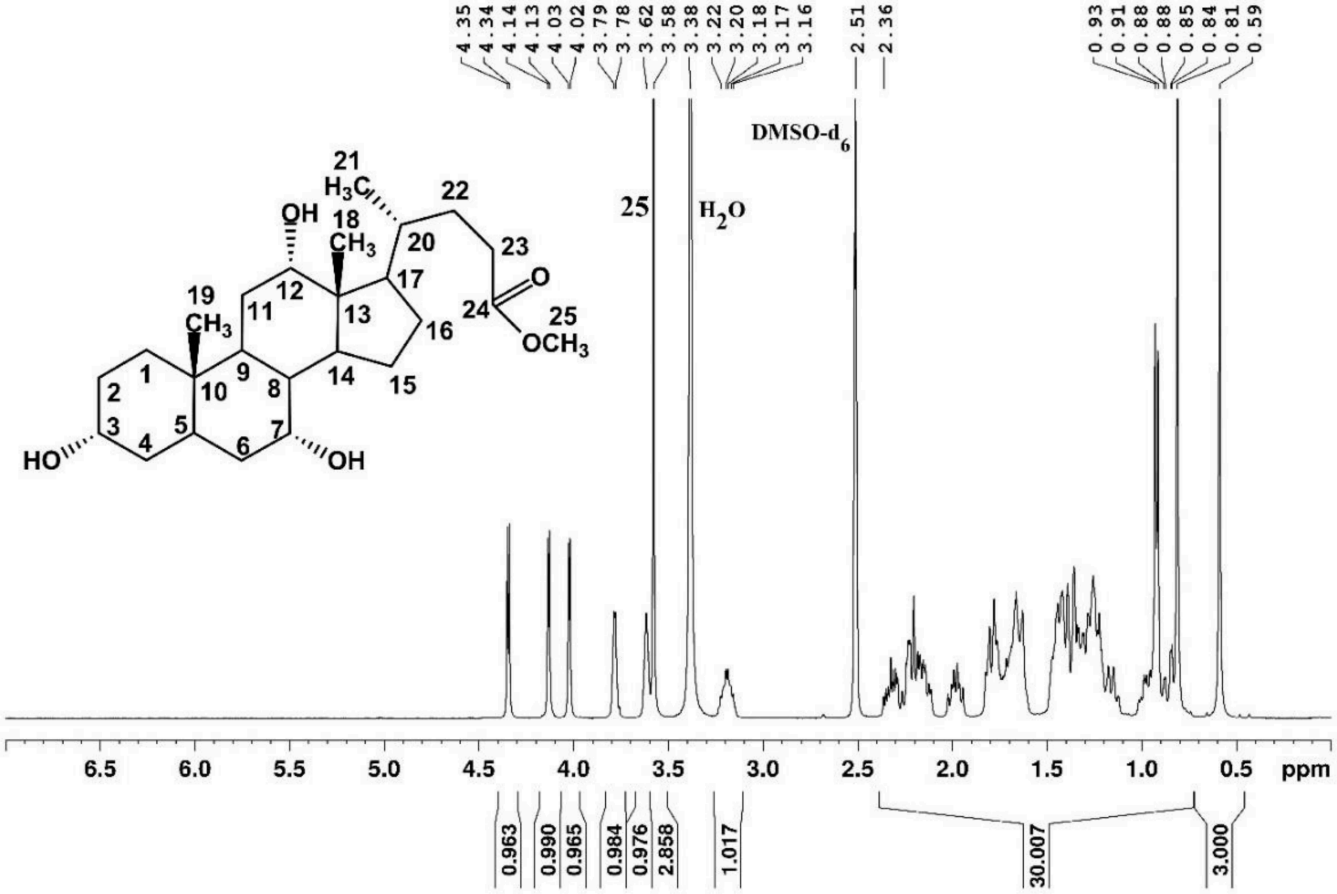
The ^1^H NMR spectrum of compound **2**.

**Figure 6:**
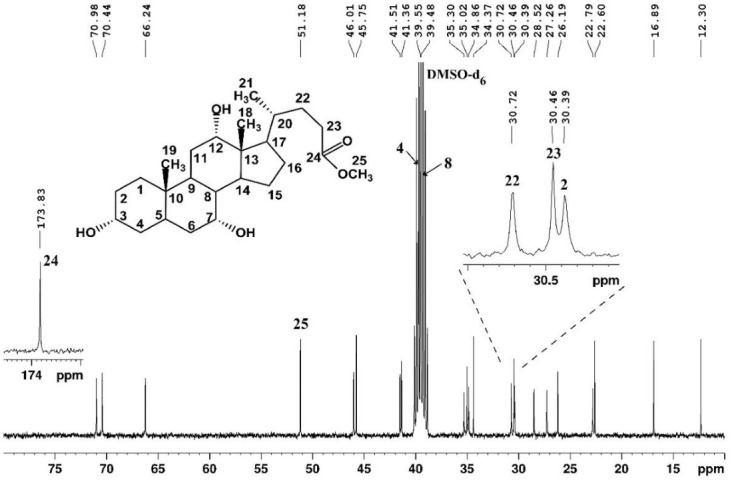
The ^13^C NMR spectrum of compound **2**.

Methyl 3α,7α,12α-trihydroxy-5β-cholane-24-oate (**2**) (Scheme 1) was obtained by esterification of the carboxyl group of CA (**1**) with absolute methanol in the presence of PTSA, which acts as a protonic catalyst that increases the electrophilicity of the carbonyl group and facilitates the nucleophilic attack of the alcohol. The use of PTSA is preferred over mineral acids as catalysts due to its milder nature and the higher yield of the reaction product. The structural identity of product **2** was established using 1D, 2D NMR, FTIR, and ESI-MS (+) analyses. The ^1^H (Figure 5) and ^13^C NMR spectra (Figure 6) confirmed ester formation, characterized by a methoxy group signal at 3.58 ppm (H25) and 51.18 ppm (C25). All ^1^H and ^13^C NMR assignments were also validated by ^1^H,^1^H COSY (Figure S8), ^1^H,^13^C HSQC (Figure S9), and ^1^H,^13^C HMBC spectra (Figure S10). FTIR analysis (Figure S5b) confirms the conversion of compound 1 to compound 2 via esterification, evidenced by the disappearance of C=O (1715 cm^−1^) and OH (3276–3170 cm^−1^) signals and the appearance of ester C=O (1736 cm^−1^), CH_3_ (2870, 1436 cm^−1^), and C–O–C (1194 cm^−1^) bands.

Deconvolution (Figure S6b) reveals that OH groups at C3, C7, and C12 remain intact, existing in both free and hydrogen-bonded states. The ESI-MS (+) spectrum of compound **2** (Figure S7b) showed peaks that were in accordance with the molecular weight of the sodiated monomer (*m*/*z* 445.2710) and its corresponding protonated (*m*/*z* 845.5103) and sodiated dimeric (*m*/*z* 868.2158) forms, respectively.

**Figure 7:**
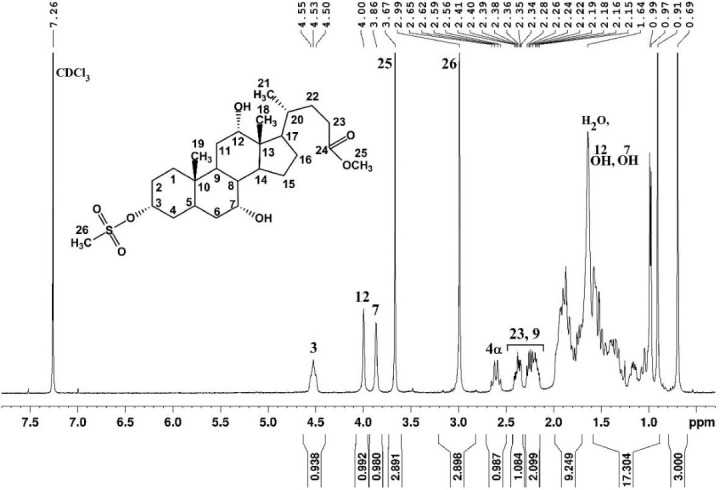
The ^1^H NMR spectrum of compound **3**.

**Figure 8:**
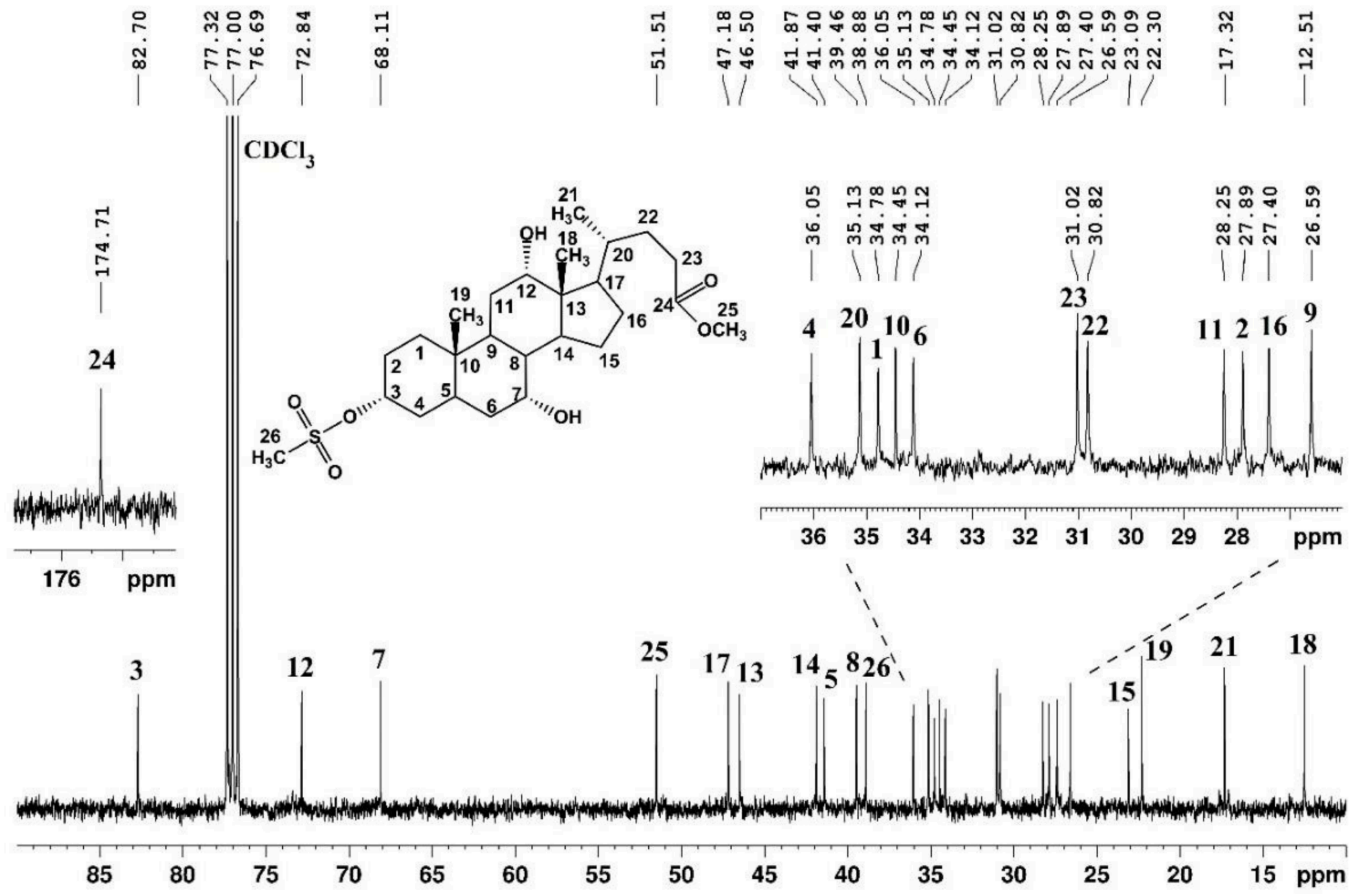
The ^13^C NMR spectrum of compound **3**.

Mesylation of product **2** with mesyl chloride (Scheme 1) in the presence of TEA, which neutralized the HCl formed during the reaction and assisted in the deprotonation of the alcohol, allowed the isolation of compound **3**. The 1D, 2D NMR, FTIR, and ESI-MS (+) spectra indicated the successful introduction of the mesyl group onto C3. The newly introduced group caused a significant deshielding of neighboring protons, shifting the H3α and H4α signals to 4.53 ppm and 2.60 ppm, respectively. The occurrence of a singlet at 3 ppm, assigned to the methyl protons (H26) of the mesyloxy group, also confirmed the formation of compound **3** (Figure 7). Similarly, the ^13^C NMR spectrum (Figure 8) revealed the C3 signal at 82.70 ppm, the most downfield-shifted resonance due to the proximity of the mesyl group. The signal attributed to the C26 carbon of the mesyloxy group was observed at 39 ppm. All signal attributions were confirmed by 2D NMR experiments (Figures S11–S13). The FTIR spectrum of product **3** (Figure S5c) confirmed the introduction of the mesyl group at the C3 position of the steroidal nucleus, as evidenced by two strong bands characteristic of the sulfonate group: an asymmetric stretching vibration at 1334 cm^−1^ and a symmetric stretching vibration at 1173 cm^−1^. The characteristic bending vibration of the CH_3_ group from the mesyloxy moiety was seen at 1371 cm^−1^. The deconvolution of the infrared spectrum of compound **3** in the 3800–3080 cm^−1^ spectral range (Figure S6c) showed a series of hidden peaks that were redshifted by 10–50 cm^−1^ compared to those of compound **2**, confirming the success of the mesylation reaction and the formation of new intermolecular H-bonding interactions involving the mesyl group.

The presence of this moiety reorganized the pre-existing intermolecular associations within compound **2**. The ESI-MS (+) analysis of compound **3** (Figure S7c) displayed the sodiated monomer at *m*/*z* 523.4809 and the signal at *m*/*z* 1001.4532, representing the protonated self-associated dimer.

**Figure 9:**
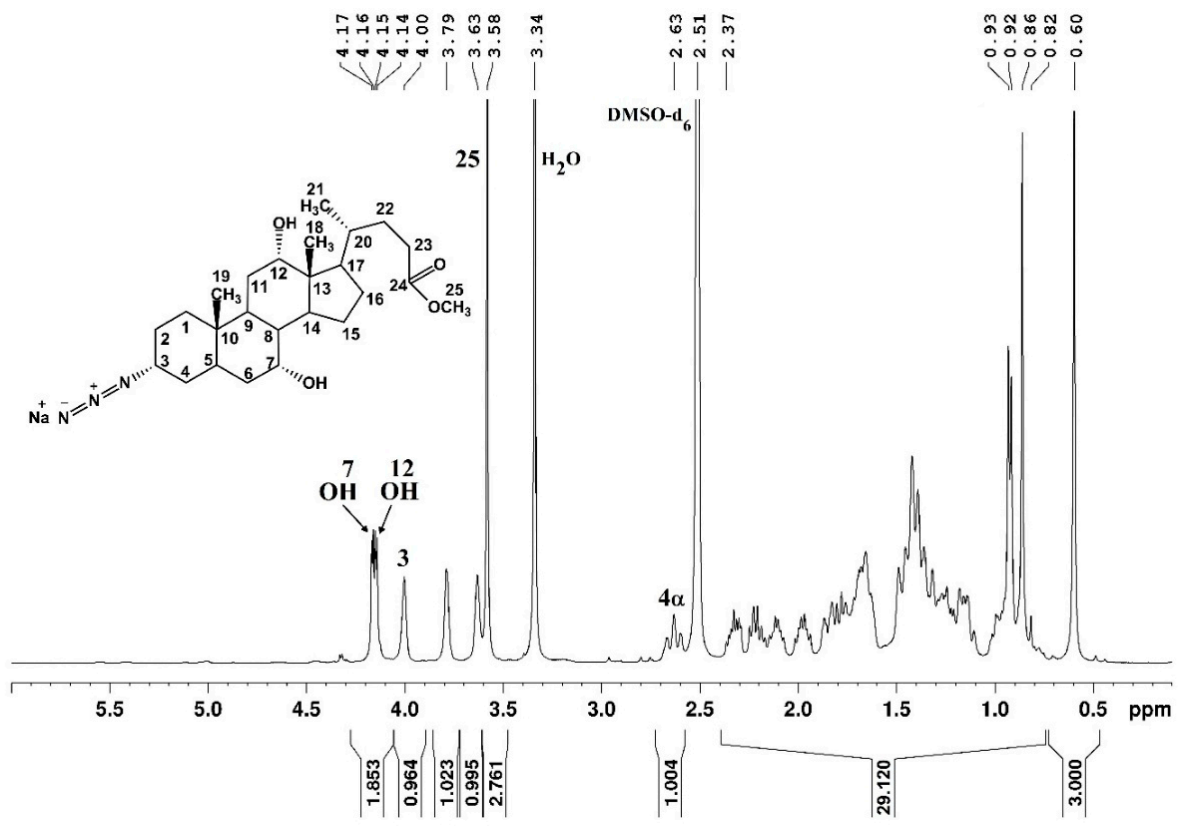
The ^1^H NMR spectrum of compound **4**.

The azidation of compound **3** (Scheme 1), carried out with sodium azide at 40 °C, afforded the obtaining of product **4**. The NMR, FTIR, and ESI-MS (+) techniques showed the successful synthesis of the azide compound. The examination of its ^1^H NMR spectrum (Figure 9) revealed that the azide substituent induced a significant deshielding effect on neighboring protons.

**Figure 10:**
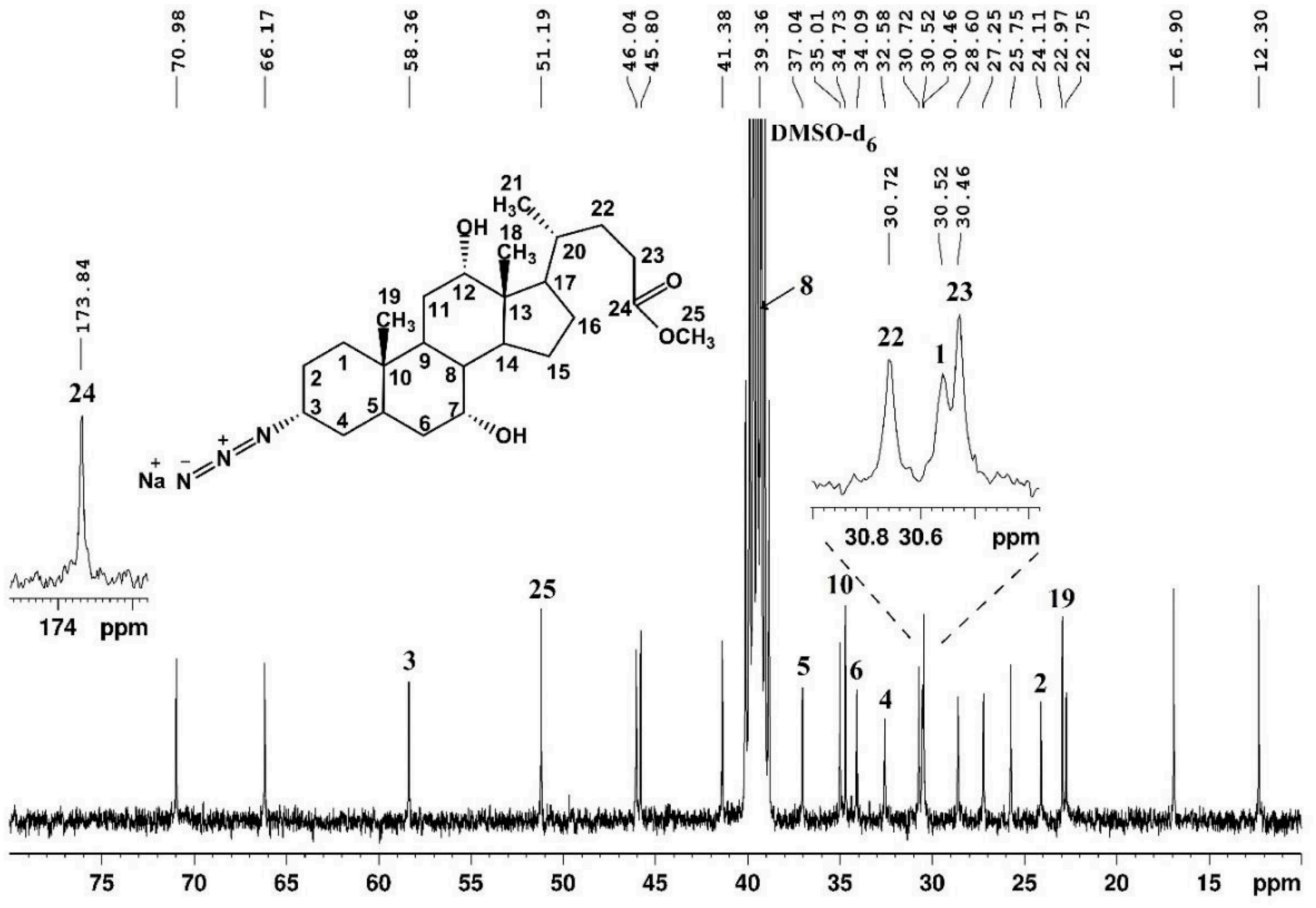
The ^13^C NMR spectrum of compound **4**.

Notably, the H3 proton, directly attached to the azide-substituted C3, was the most deshielded, its signal being shifted to 4.0 ppm, while the H4α signal shifted to 2.63 ppm. The ^13^C NMR spectrum of compound **4** (Figure 10) showed significant shifts of the signals for the carbon atoms located in the vicinity of the azide group. Therefore, the C3 signal moved upfield to 58.36 ppm. Similar effects were observed for the C2 (24.11 ppm), C4 (32.58 ppm), C1 (30.52 ppm), and C5 (37.04 ppm). The DEPT135 experiment (Figure S14) was used to verify carbon assignments and revealed that the C8 chemical signal overlapped with the residual DMSO-*d*_6_ peak. The COSY spectrum (Figure S15) was used to distinguish between the chemical shifts of the OH7 and OH12 groups by identifying the specific cross-peaks of the geminally J-coupled protons, OH7α–H7β and OH12α–H12β. Additionally, the assignment of H4α was validated through its vicinal H4α–H5β cross-correlation. All resonance assignments were also validated by one-bond H-C correlations from the HSQC spectrum (Figure S16) and multiple-bond H-C correlations occurring in the HMBC spectrum (Figure S17). The formation of compound **4** was proved by its FTIR spectrum (Figure S18a), which showed the absorption of the azide band at 2097 cm^−1^, together with the disappearance of the absorbance bands specific to the sulfonate group. The deconvolution of its spectral range between 3800–3080 cm^−1^ (Figure S6d) showed that the peaks associated with the free and intermolecular H-bonded OH stretching vibrations, coming from the hydroxyl groups bound to C7 and C12, were located at positions similar to those observed in compound **3**, indicating that the azidation resulted in minimal perturbation of the existing intermolecular H-bonding network. The ESI-MS (+) analysis of compound **4** (Figure S19a) showed the peak at *m*/*z* 895.5296 corresponding to the protonated dimer, while the monomeric species was identified as a protonated adduct at *m*/*z* 448.2846.

**Figure 11:**
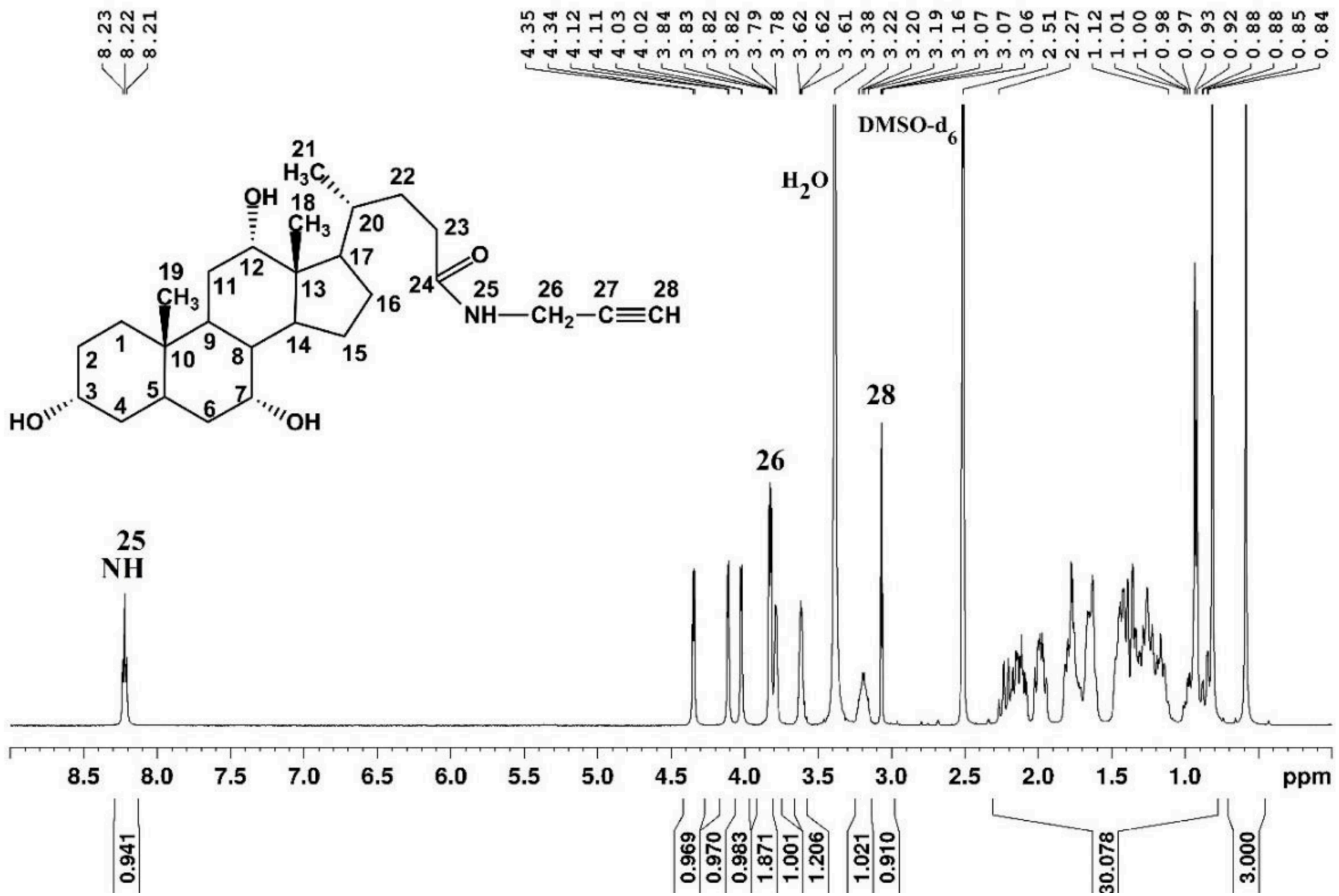
The ^1^H NMR spectrum of compound **5**.

**Figure 12:**
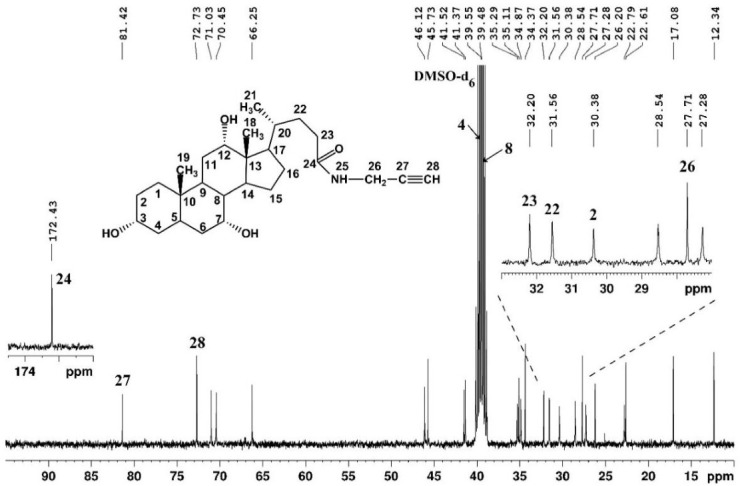
The ^13^C spectrum of compound **5**.

Compound **5** was obtained at room temperature by the propargylation of the cholanic acid (**1**) with propargyl amine, in the presence of EDC-HCl as a coupling catalyst and HOBt as a nucleophilic catalyst (Scheme 1). The structural identity of the propargylated product was demonstrated by its 1D, 2D NMR, FTIR, and ESI-MS (+) spectra. Thus, the ^1^H NMR spectrum (Figure 11) exhibited three additional signals compared to that of cholanic acid: a triplet at 3.06 ppm, characteristic of the methine proton (H28), a doublet of doublets at 3.82 ppm, specific to the methylene protons (H26), and the triplet at 8.22 ppm, attributed to the NH25. Equally, the ^13^C NMR spectrum (Figure 12) of product **5** showed three additional shifts attributable to the propargyl group at 27.71 ppm (C26), 72.73 ppm (C28), and 81.42 ppm (C27). Moreover, the carboxyl C24 signal was upfield shifted to 172.43 ppm, while the C22 and C23 signals appeared downfield shifted at 31.56 ppm and 32.20 ppm, respectively, due to the influence of the electron-withdrawing propargyl group. All ^1^H and ^13^C NMR assignments were confirmed by 2D NMR experiments. The COSY spectrum (Figure S20) showed the cross-peaks corresponding to the couplings between NH25 and H26 and between H26 and H28. The assignments of C26 and C28 in the ^13^C NMR spectrum were proved by the direct one-bond correlations H26-C26 and H28-C28 in the HSQC spectrum (Figure S21). Finally, the HMBC spectrum (Figure S22) revealed long-range bond correlations between the protons and carbons of the propargyl substituent, such as H27-C28, H23-C24, NH25-C24, NH25-C26 (two-bond correlations), and H26-C28, H26-C24 (three-bond correlations), confirming the structure. FTIR spectroscopy (Figure S18b) also indicated the successful synthesis of product **5** by the existence of the stretching bands associated with functional groups, such as the C-H (ethynyl) (3298 cm^−1^), the C≡C (ethynyl) (2123 cm^−1^), the free (3432 cm^−1^) and intermolecular H-bonded N-H (amide) (3384 cm^−1^), and the Amide I (1645 cm^−1^) and Amide II absorption bands (1535 cm^−1^).

Furthermore, in the FTIR spectrum of product **5**, as a result of the propargylation reaction, some absorption bands characteristic of CA disappeared, such as O-H (acid) (3276, 3218, and 3170 cm^−1^) and the C=O (acid) (1715 cm^−1^). The deconvolution of the FTIR spectrum of compound **5** in the 3800–3080 cm^−1^ spectral range revealed additional vibrational modes (Figure S6e) compared to that of the broad O-H absorption envelope specific to compound **1** (Figure S6a). Thus, the bathochromic shifts observed in the O-H bands indicated that the introduction of the amide moiety improved the strength of the intermolecular H-bonding within the propargylated compound. Product **5** exhibited in its ESI-MS (+) spectrum (Figure S19b) a sodium adduct at 468.2277 (*m*/*z*), while its protonated and sodiated dimeric species were visible at 891.4286 (*m*/*z*) and 913.4999 (*m*/*z*), respectively.

**Figure 13:**
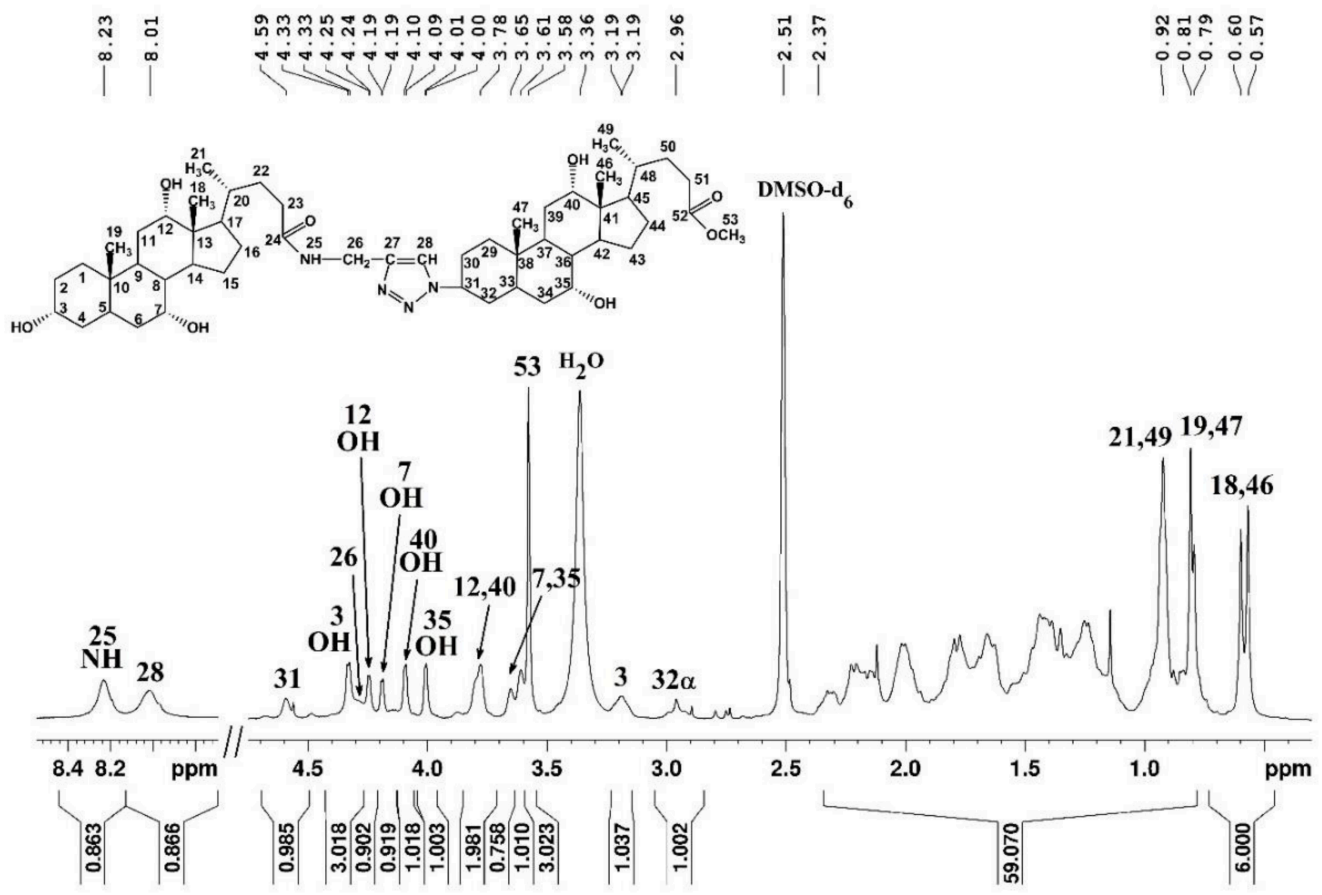
The ^1^H NMR spectrum of compound **6**.

**Figure 14:**
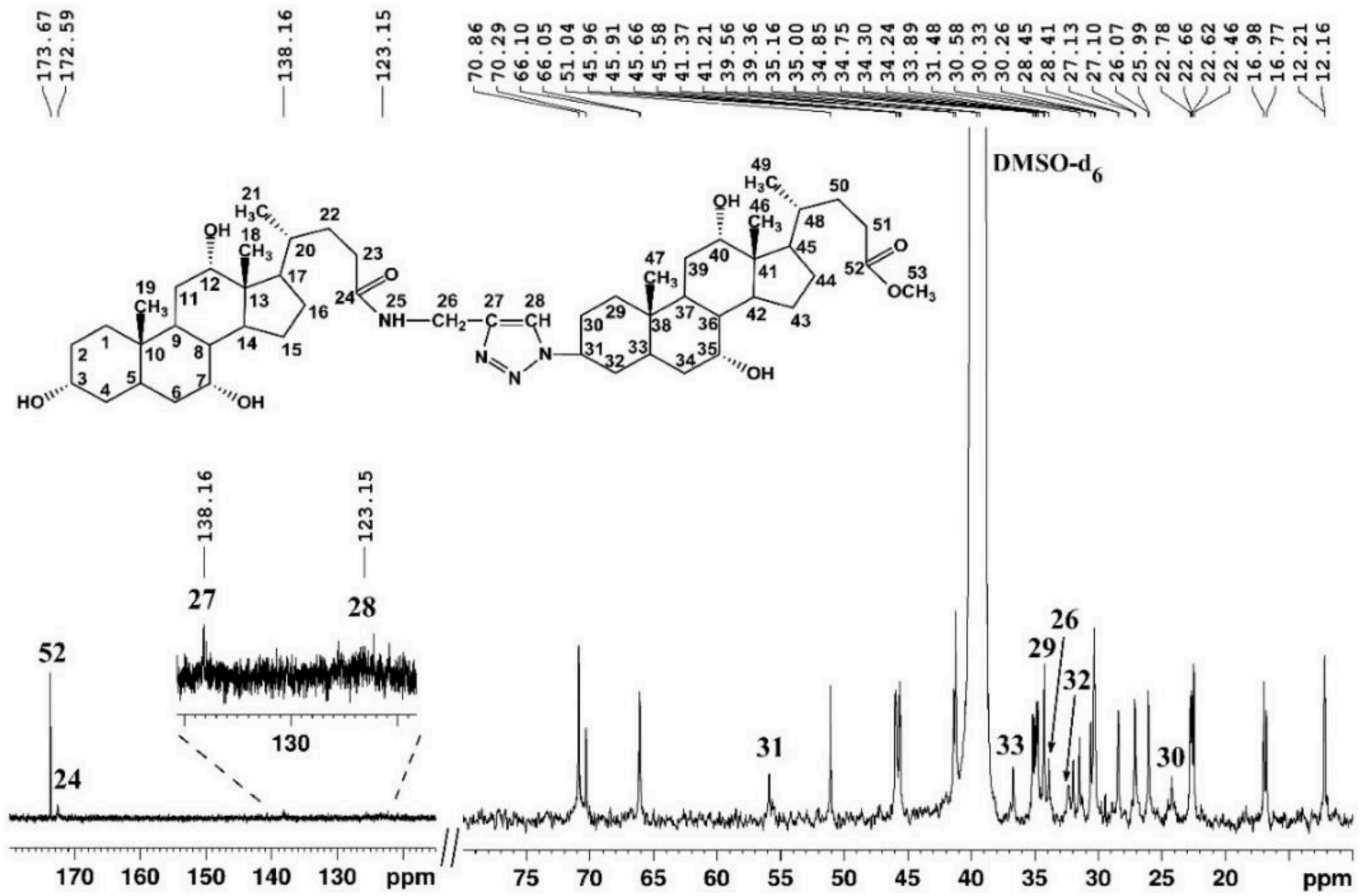
The ^13^C NMR spectrum of compound **6**.

**Figure 15:**
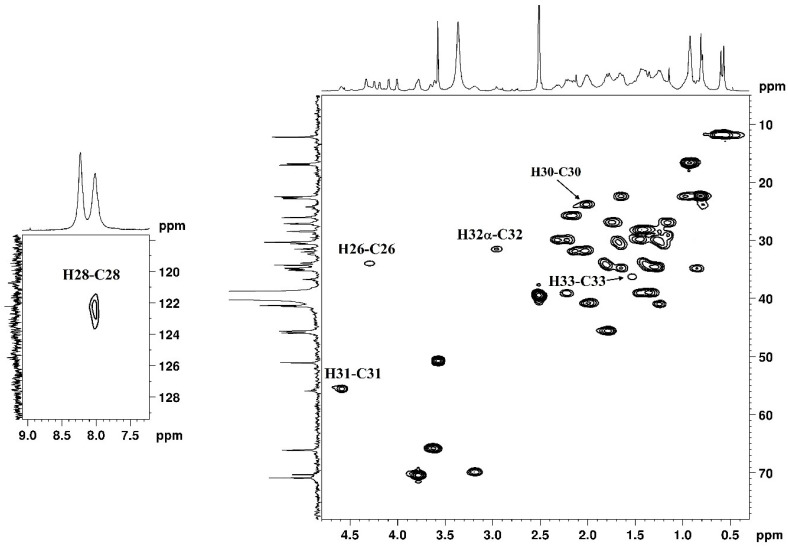
The ^1^H,^13^C HSQC spectrum of compound **6**.

The CuAAC reaction between azide (**4**) and the propargylated product (**5**), in the presence of Cu^+1^ (CuI), PMDETA, and ascorbic acid, afforded the formation of the 1,4-disubstituted 1,2,3-triazole (compound **6**) (Scheme 2). PMDETA was the ligand that stabilized the Cu^+1^ oxidation state, while ascorbic acid was the reducing agent, which kept any Cu^+2^ in the active Cu^+1^ state. NMR, FTIR, and ESI-MS (+) spectra of the click dimer proved its formation. Thus, the occurrence of the 1,2,3-triazole moiety in compound **6** was supported by the presence in its ^1^H NMR spectrum (Figure 13) of the H28 signal at 8.01 ppm and by the downfield shift of the H31 signal from 4.0 ppm in the CA spectrum to 4.59 ppm [44]. The deshielding effect of the nitrogen atoms from the 1,2,3-triazole ring was reflected in the downfield shift of the H32α resonance from 2.63 ppm in compound **4** to 2.96 ppm in compound **6**, and in the appearance of the broad H26 signal at 4.30 ppm in the dimer (**6**) compared with 3.82 ppm in product **5**. Because the dimer (**6**) has two steroidal nuclei that were not identically substituted, its ^1^H NMR spectrum presented signals that were not completely overlapped, but rather, within a very narrow ppm range for the same functional groups located in homologous positions, such as methyl groups (H18 and H46, H19 and H47, or H21 and H49), OH groups (OH7 and OH35 or OH12 and OH40), or the geminal protons of OH groups (H7 and H35 or H12 and H40). In the detailed region of the ^13^C NMR spectrum (Figure 14), the weak signals corresponding to C27 and C28 were also observed. The broadening of these signals is attributed to the slow dynamics associated with the occurrence of the 1,2,3-triazole ring [34]. The correct attribution of H26, H31, and H32α signals in the ^1^H NMR spectrum was sustained by HSQC correlations (Figure 15). Moreover, the left region of the HSQC spectrum, which showed the H28-C28 cross peak, afforded the assignment of the C28 signal despite its low intensity in the ^13^C NMR spectrum.

In the FTIR spectrum (Figure S18c), the C–H (triazolyl) stretching band (3100–3180 cm^−1^), which could prove the formation of the steroidal derivative **6**, was absent because the solitary C–H bond was significantly balanced by a large number of aliphatic C–H bonds from the steroidal skeletons. Other papers also reported the absence of the C–H (triazolyl) stretching band in the FTIR spectrum of click products [45,46]. In such cases, the successful formation of dimer (**6**) was confirmed by the disappearance of the precursor peaks, namely the azide (2097 cm^−1^), the C≡C (2122 cm^−1^), and the C-H (ethynyl) (3313 cm^−1^), along with the presence of the absorbance bands characteristic of the functional groups within the dimer structure. The deconvolution of the FTIR spectrum of the dimer in the 3800–3080 cm^−1^ spectral range (Figure S6f) showed the occurrence of the absorption bands assigned to the stretching vibrations of the free and intermolecular H-bonded O-H and N-H groups. The position of these bands was blue-shifted by 20 cm^−1^ as compared with those of the compound **4**, while the N-H sharper bands characteristic of compound **5** disappeared in the FTIR spectrum of the dimer due to restricted conformational changes and reduced intermolecular H-bonding interactions, confirming the success of the coupling reaction. The ESI-MS (+) spectrum of product **6** displayed the protonated molecular ion at *m*/*z* 893.5550, along with the corresponding sodium adduct at *m*/*z* 915.4697 (Figure S19c).

### 2.2. Convertible Pockets Formed by Steroidal Dimer

The dimer (**6**) can form adaptive molecular pockets depending on the solvent polarity, due to its facial amphiphilicity and curved steroidal skeleton [18,28,29]. The “inversion” is a conformational switch in the 3D shape of the host molecule, produced by solvophobic interactions. The dimer molecule presented hydrophilic/hydrophobic binding pockets depending on the medium polarity. Therefore, in a nonpolar solvent, the host molecule exposed its polar faces inward, creating hydrophilic cavities appropriate for binding hydrophilic guest molecules, while in a polar solvent, the host molecule adopted a specific conformation to minimize unfavorable interactions between the nonpolar faces and water, directing the nonpolar faces toward the interior and creating hydrophobic cavities capable of encapsulating lipophilic guest molecules. This reversible shift of cholic acid pockets in solvents with different polarities was studied using CR dye as the ionic compound, which could be trapped in the hydrophilic cavities of cholanic product, and pyrene as the lipophilic product held by the hydrophobic pockets of compound **6**. The solubilization of the polar dye in organic solvents due to its inclusion in the host–guest complex was investigated through UV–Vis absorbance studies, while the dissolution of pyrene in water by its encapsulation in the host–guest complex was studied by the steady-state fluorescence technique.

#### 2.2.1. Hydrophilic Pockets Formed by Cholic Acid Dimer

**Figure 16:**
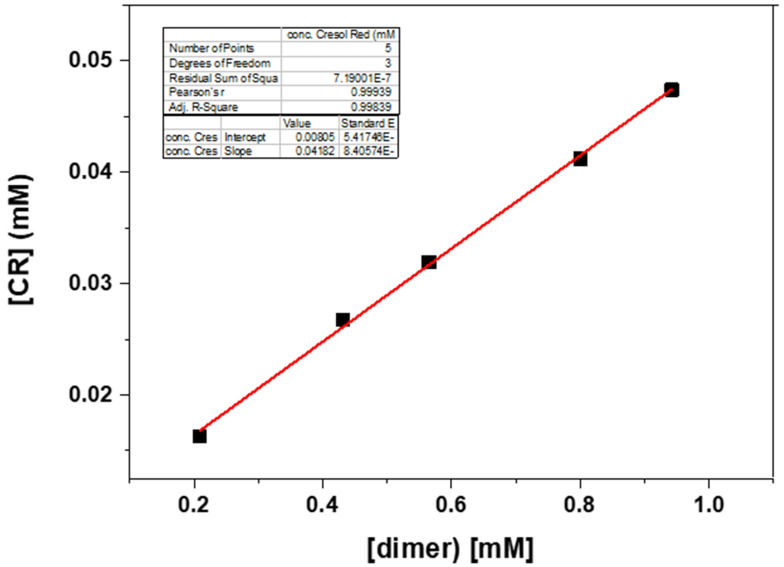
The variation of CR dye concentration vs. the dimer concentration.

Cresol Red sodium salt, CR, a hydrophilic dye, showed a partial solubility in slightly polar organic solvents, like chloroform. Its solubilization in such solvents, proved by UV–Vis absorbance studies, could be explained by the retention of ionic dye (guest) within the hydrophilic cavities formed by the bile acid dimer (host) through hydrogen bonding, ion–dipole, and dipole–dipole interactions. Similar absorption studies showed the storage of the same polar dye within the hydrophilic pockets formed by different CA-based dimers or trimers in nonpolar or slightly polar media. The polar dye concentration, held by the host, was determined by using spectrophotometric absorbance measurements of homogeneous solutions obtained by filtering the mixture of solid CR dye and increasing concentrations of steroidal product. Figure 16 indicates that the relationship between the host and guest concentrations was linear, confirming the formation of the host–guest complex and facilitating the solubilization of the polar dye in chloroform.

**Figure 17:**
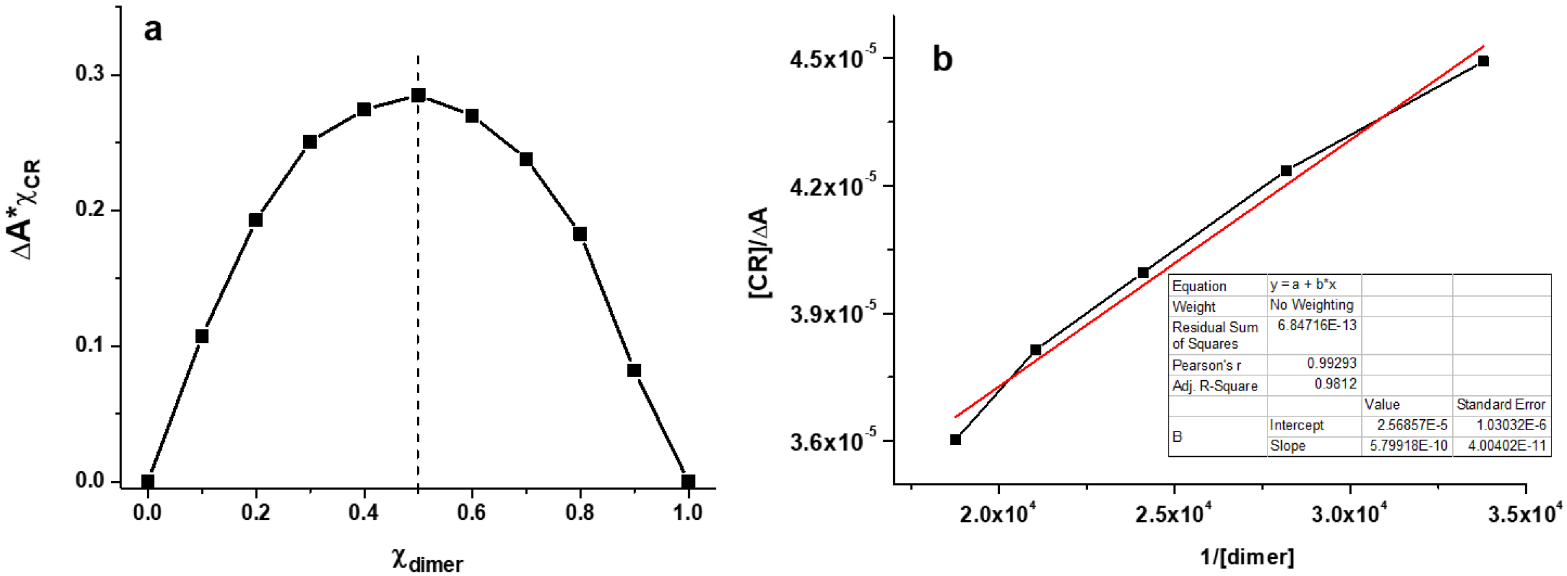
Job’s plot diagram (**a**) and determination of K_a_ for dimer–CR complex (**b**).

Stoichiometry of the complex dimer-CR was determined by means of UV–Vis study (Figure S23) via several solutions in which the mole fractions of host and guest were varied, while maintaining a constant total concentration and volume (Table S1). Job’s plot was constructed by charting χ_CR_ × ΔA as a function of the mole fraction of the dimer (χ_dimer_) from 0 to 1, where ΔA = A_max_ − A_i_, A_max_—the absorbance at λ = 426 nm and χ_CR_ = 1; A_i_—the absorbance at λ = 426 nm and χ_CR_ = 0.1–0.9; i = 2–10. The resulting graph showed the peak position at 0.5, proving the stoichiometry dimer: CR = 1:1 (Figure 17a). The stability constant (K_a_) of the complex dimer–CR was evaluated by the Hildebrand–Benesi equation (Equation (1)), with the main parameters depicted in Table S2.

(1) $$\frac{\left[CR\right]}{∆A}=\frac{1}{\varepsilon }+\frac{1}{\left[dimer\right]\varepsilon K_{a}}$$

where [CR], [dimer], and ε are the concentrations of CR, dimer, and the molar absorption coefficient, respectively; A = A − A_0_, where A is the absorbance at each molar fraction of the dimer to the CR, and A_0_ is the absorbance in the absence of the dimer.

The plot of ([CR]/ΔA) against 1/[dimer] (Figure 17b) is linear (R^2^ = 0.9812), suggesting that the predominant species in solution was host/guest = 1:1. The stability constant (K_a_ = 44 × 10^3^ M^−1^), determined from the ratio between the intercept and slope of the same graph, showed a moderate stability of the inclusion complex in solution. The dissociation constant K_d_ better highlights the host/guest affinity and can be calculated as the inverse of the association constant, K_d_ = 1/K_a_ = 22.7 × 10^−6^M (22.7 μM).

#### 2.2.2. Hydrophobic Pockets Formed by Cholic Acid Dimer

**Figure 18:**
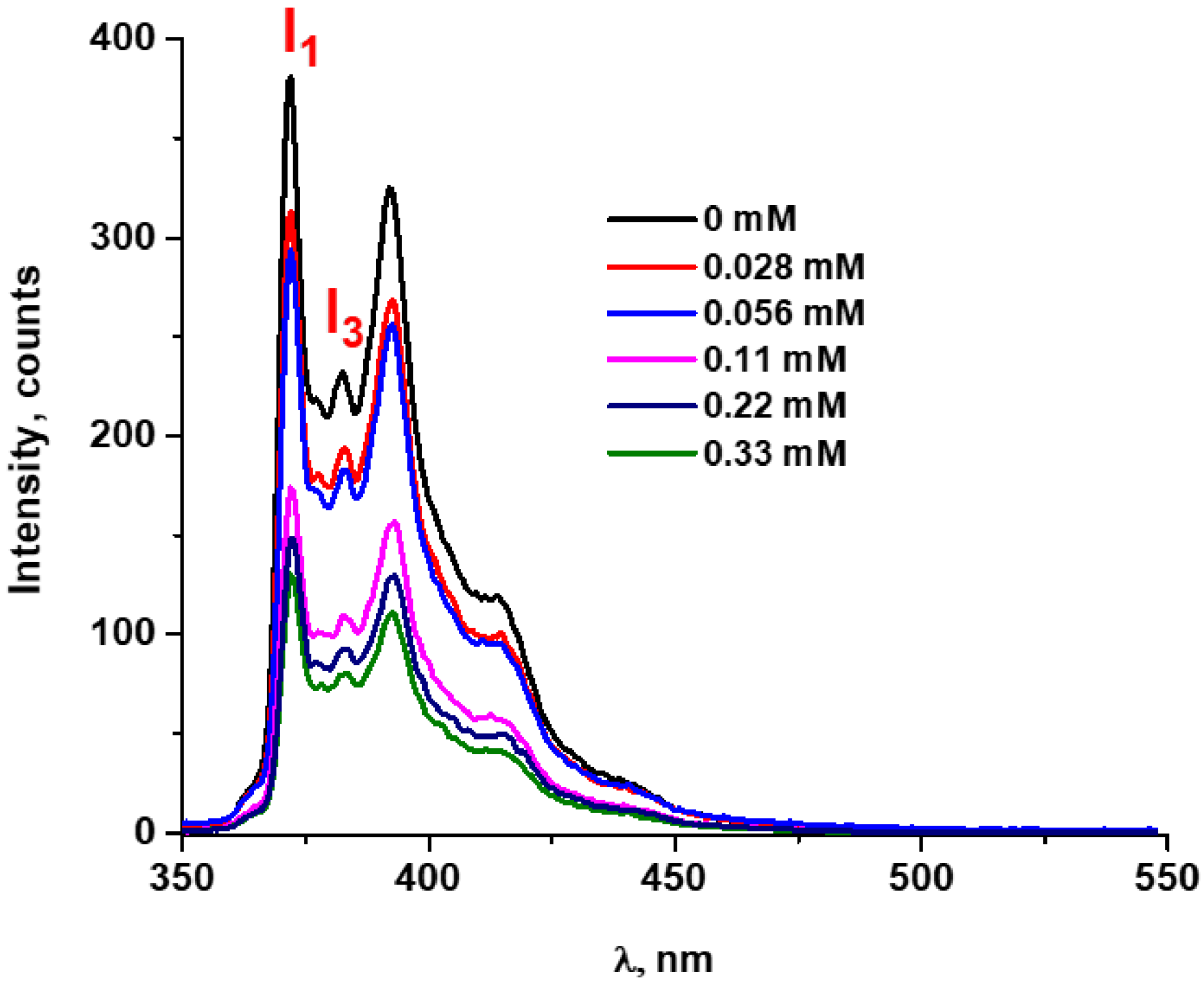
Fluorescence spectra of pyrene in the absence and the presence of various concentrations of the dimer (0–0.33 mM).

Pyrene was chosen as the fluorescence probe to prove the formation of the lipophilic pockets of the dimer in aqueous media. The chromophore was retained by the dimer through hydrophobic interactions, van der Waals, and CH–π forces, along with entropic effects. The hydrophobic interactions are the main forces for complex formation, and pyrene lipophilicity afforded its inclusion within the hydrophobic pockets of the steroidal dimer. The planar geometry of the fluorophore allowed it to fit comfortably within the steroidal cavities of product **6**, thereby maximizing van der Waals forces that stabilized the complex. The CH–π interactions, formed between the hydrogen atoms from the dimer backbone and the pyrene π-electron cloud, positioned the guest molecules within the dimer hydrophobic pockets. The inclusion of the guest molecules in the lipophilic cavities of compound **6** was also facilitated by the entropic effect, which was determined by the release of structured water molecules that had previously surrounded the pyrene molecules into the bulk solvent, thus making the entire assembly process energetically spontaneous. The I_1_/I_3_ ratio of pyrene solution in the absence of the dimer, calculated from Figure 18, was 1.66, suggesting a polar (water) environment [47]. The addition of different concentrations of the dimer (from 0.028 mm to 0.33 mM) resulted in a slight decrease in the polarity parameter, from 1.66 to 1.59. This confirmed the retention of the chromophore probe within the lipophilic cavities of compound **6**. The observed I_1_/I_3_ ratio indicated that pyrene was only partially shielded from the aqueous environment by the flexible dimeric framework and the polar triazole linkers. The relatively flexible skeleton of the dimer–pyrene complex allowed partial solvent penetration, preventing the ratio from dropping to the low levels (<1.0) typically seen in more rigid or tightly packed hydrophobic cavities, like those formed by β-cyclodextrin [48] or cucurbiturils (CB) (especially CB[7]) [49].

Both static and dynamic quenching require molecular contact between the fluorophore (pyrene) and the quencher (dimer). In the case of collisional quenching, the quencher must diffuse to the fluorophore during the lifetime of the excited state. In static quenching, a complex is formed between the fluorophore and the quencher, and this complex is non-fluorescent. In many cases, the fluorophore can be quenched both by collisions and by complex formation with the same quencher.

Based on the evolution of the pyrene fluorescence spectra after the addition of different concentrations of dimer, the mechanism of the fluorescence quenching was analyzed. Fluorescence quenching can occur through static, dynamic, or combined mechanisms.

The Stern-Volmer equation (Equation (2)) is a fundamental mathematical model used to describe the kinetics of fluorescence quenching. This relationship is critical for distinguishing between dynamic quenching, which occurs through collisional encounters, and static quenching, which results from the formation of a non-fluorescent ground-state complex.

(2) $$\frac{F_{0}}{F}=1+K_{SV}\cdot C=1+k_{q}\tau_{0}C$$

(3) $${K_{SV}=k}_{q}\tau_{0}$$

(4) $$k_{q}=\frac{8\cdot R\cdot T}{3\cdot \eta }$$

where F_0_ is the fluorescence intensity of pyrene in the absence of the dimer, F is the fluorescence intensity of pyrene in the presence of the dimer, C is the concentration of the dimer, K_SV_ is the Stern–Volmer constant, k_q_ is the bimolecular quenching rate constant, τ_0_ is the average fluorescence lifetime of pyrene without quencher, T is temperature (°C), η is solvent viscosity, and R is the gas constant.

To characterize the static quenching mechanism, the relationship between fluorescence intensity and quencher concentration was analyzed using the double-logarithmic regression equation (Equation (5)). The linearity of the plot permits the calculation of the binding constant (K_a_) and the number of binding sites per molecule (n).

(5) $$\frac{\log(F_{0}-F)}{F}=\log K_{a}+n\cdot \log C$$

where K_a_ is the association constant between pyrene and dimer, n is the number of binding sites per molecule, and C is the concentration of the dimer.

**Figure 19:**
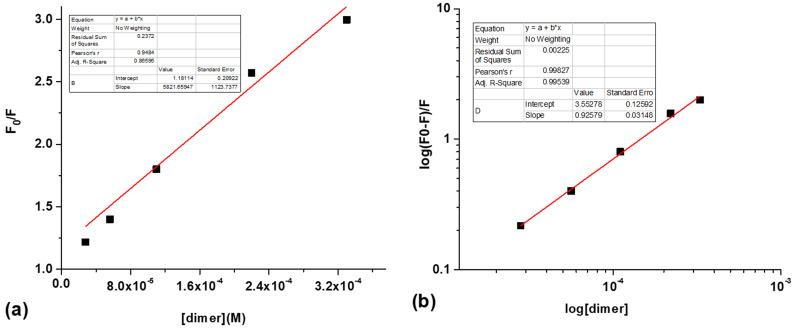
The linearity of the Stern–Volmer equations from the dependence of the fluorescence of pyrene at λ = 372 nm on the concentration of the dimer (**a**); the double-log plots of pyrene fluorescence at λ = 372 nm and the quenching effect of the dimer (**b**).

Based on the slope of Figure 19b, the value of n was determined to be 0.93, suggesting a single pyrene binding site per dimer molecule. From the y-intercept of Figure 19a, the association constant, K_a_, was calculated as 3.548 ·10^3^ M^−1^ (log K_a_ = 3.55), suggesting a moderate affinity between pyrene and the dimer in solution. The dissociation constant, K_d_, better highlights the host: guest affinity and can be calculated as the inverse of the association constant: K_d_ = 1/Ka; K_d_ = 2.82 · 10^−4^ M = 0.282 mM = 282 μM. 

The upward curvature observed in the Stern-Volmer plot (Figure 19a) suggested a combined quenching mechanism (dynamic by collision and static by the formation of a complex dimer-pyrene). Consequently, the apparent quenching constant, K_app_, was calculated to evaluate the total quenching efficiency (Equation (6)). K_app_ defines the quencing mechanism involving both processes, either collisions or complex formation, with the same quencer, at each concentration, to allow the graphical separation of the K_D_ and K_S_. The increase in K_app_ with higher dimer concentrations confirmed that dynamic collisions are predominant, but a minor static component also contributes to the deactivation of the pyrene excited state.

(6) $$K_{app}=(\frac{F_{0}}{F}-1)/C$$

Equations (7) and (8) are used to describe the combined process of pyrene quenching [50].

(7) $$\frac{F_{0}}{F}=\left(1+K_{D}\cdot C\right)\cdot \left(1+K_{S}\cdot C\right)=1+\left(K_{D}+K_{S}\right)\cdot C+(K_{D}\cdot K_{S})\cdot C^{2}$$

where K_D_ is the constant of dynamic quenching and Ks is the constant of static quenching.

The resulting terms, obtained by expanding the combined Stern-Volmer Equation (7), represent different physical aspects of the quenching process. Thus, 1 represents the unquenched state where no quencher is present, while the linear term (K_D_ + K_S_) · C, the dominant factor at low concentrations, shows the additive effect of both dynamic and static quenching, the plot being a straight line for one type of quenching. The quadratic term (K_D_ · K_S_) · C^2^ represents the simultaneous occurrence of both mechanisms and it causes the Stern-Volmer plot to curve upward at higher concentrations.

Equation (7) can be rewritten as Equation (8).

(8) $$\frac{\frac{F_{0}}{F}-1}{C}={{(K}_{S}+K}_{D})+{(K}_{S}\cdot K_{D})\cdot C$$

**Figure 20:**
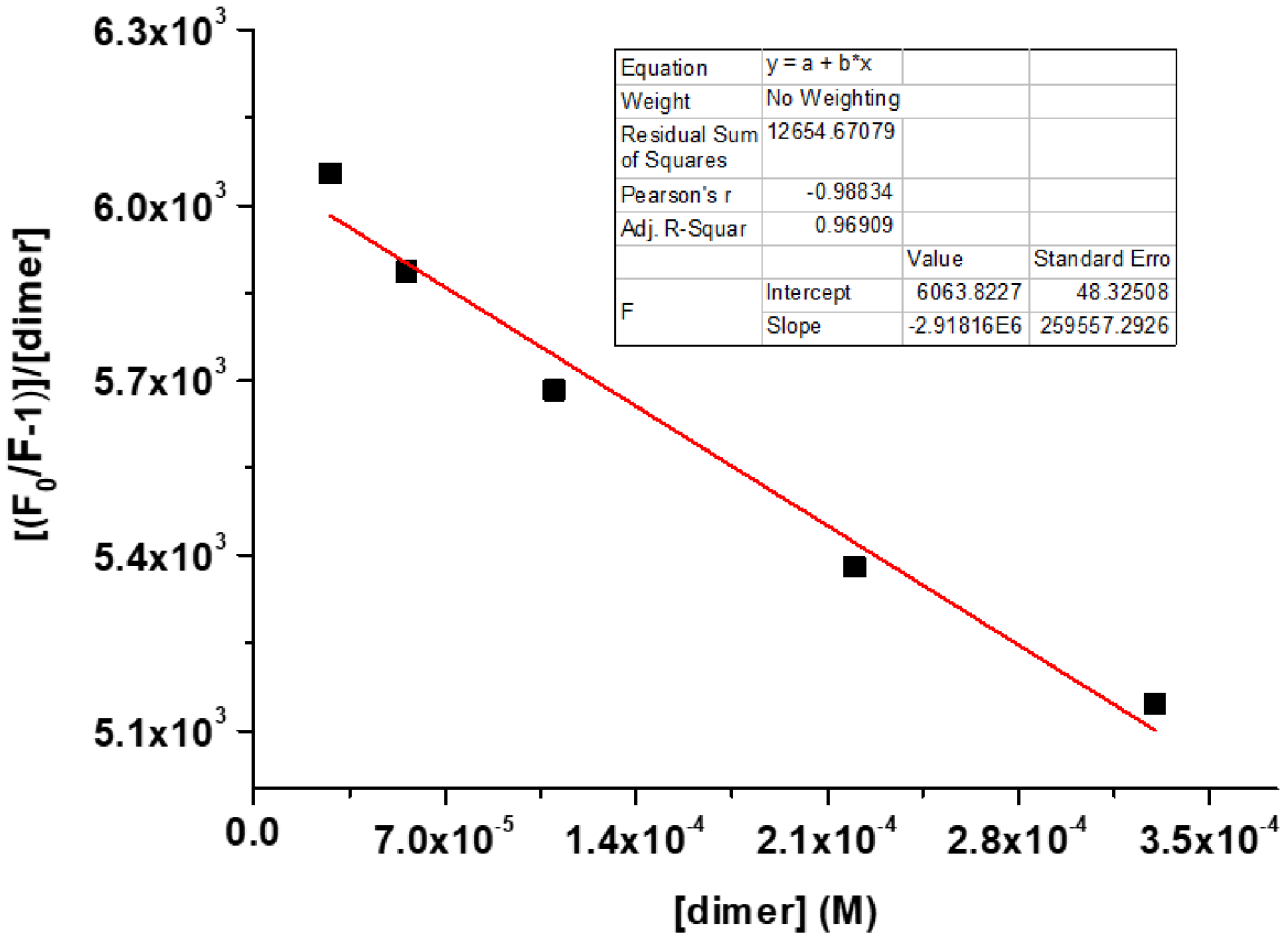
Dependence of the apparent constant, K_app_, on the dimer concentration.

From the plot contained in Figure 20, which illustrates the variation of K_app_ = {(F_0_/F − 1)/[dimer]} as a function of dimer concentration, [dimer], the product K_S_ ·K_D_ can be calculated from the slope, while the sum K_S_ + K_D_ is determined from the y-intercept.

The y-intercept of the graph (Figure 20) is defined as I = K_S_ + K_D_ = 6063.82 M^−1^, while its slope is S = K_D_·K_S_ = −2.92 × 10^6^ M^−2^. To separate the static and the dynamic quenching constants, the quadratic equation (Equation (9)) was solved:

(9) $${Ks}^{2}-Ks\cdot I+S=0$$

where Ks is the constant of static quenching, I is the intercept and S is the slope of the linear fitting plot of the graph contained in Figure 20. 

The solutions of Equation (9) were K_s_ = 5537 M^−1^ or 527 M^−1^. K_D_ value is near 5821.65 M^−1^ (Figure 19a), so the lower value was assigned to the static quenching constant. Based on the data solved by Equation (9), K_D_ = 5537 M^−1^ and K_S_ = 527 M^−1^.

K_S_ = K_app_ − K_D_ = 527 M^−1^.

The fluorescence quenching of pyrene by the dimer in aqueous solution was predominantly a dynamic or collisional process, as determined by the formula 100 · [K_D_/(K_D_ + K_S_)] = 91%, rather than the formation of a ground-state pyrene-dimer complex.

Since the solution-state studies did not fully disclose the exact binding mode of pyrene by the dimer, molecular modeling simulations were carried out. In silico studies were also conducted to clarify the binding of CR by the dimer.

#### 2.2.3. Molecular Modeling Study

**Figure 21:**
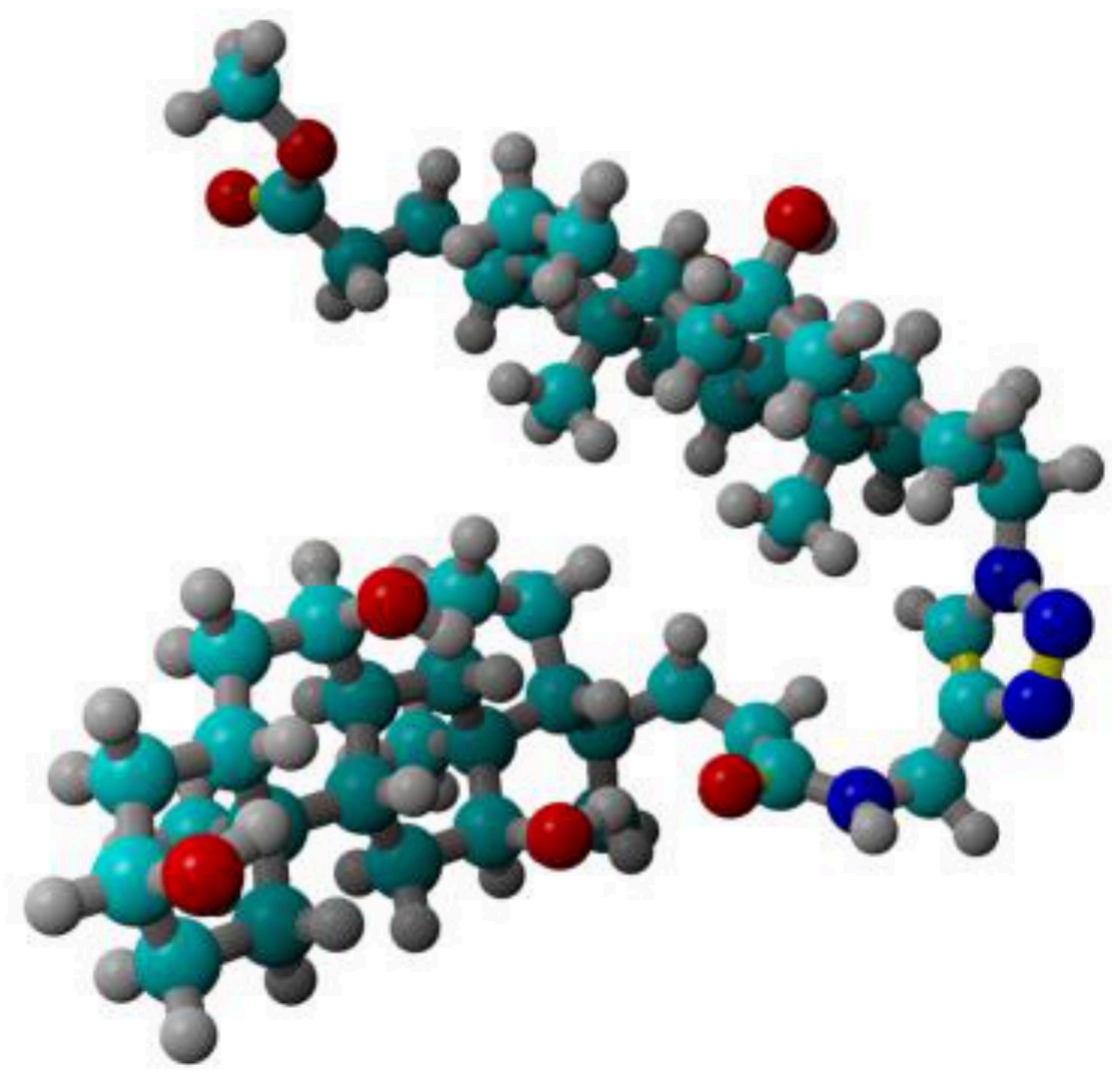
Optimized molecular geometry of the host obtained using the YASARA force field.

Molecular modeling serves as a computational tool to predict the spatial arrangement of chemical structures at an atomic level. By employing force field-based methods, such as the Yasara force field, it is possible to perform geometry optimization to identify the most stable conformation of a molecule. To better understand the dimer’s shape (Figure 21), the distances between its internal parts were calculated using an optimized model built using the Yasara force field. Based on this, a distance of 2.08 Å was found between the –OH and O = C– groups, suggesting they are close enough to interact. Meanwhile, the distance between the –NH group and the triazole nitrogen was larger, measured at 3.25 Å.

Molecular docking is an essential method for modeling non-covalent interactions between a host and a guest when their 3D conformations are known. In silico studies showed that the *syn* conformer of the cholic acid dimer was its optimal state, with a radius of gyration (R_g_) of 6.23 Å. The conformation was stabilized by an intramolecular H-bond between the OH and C = O groups of the steroidal nucleus, characterized by a bond distance of 2.08 Å and an interaction energy of 5.7 kcal/mol. The resulting docking conformations of the complex host–guest, calculated for a ratio of 1:1, were analyzed by a scoring function to determine the binding affinity (i.e., binding energy *E_b_*) and the dissociation constant (*K_d_*) values.

**Figure 22:**
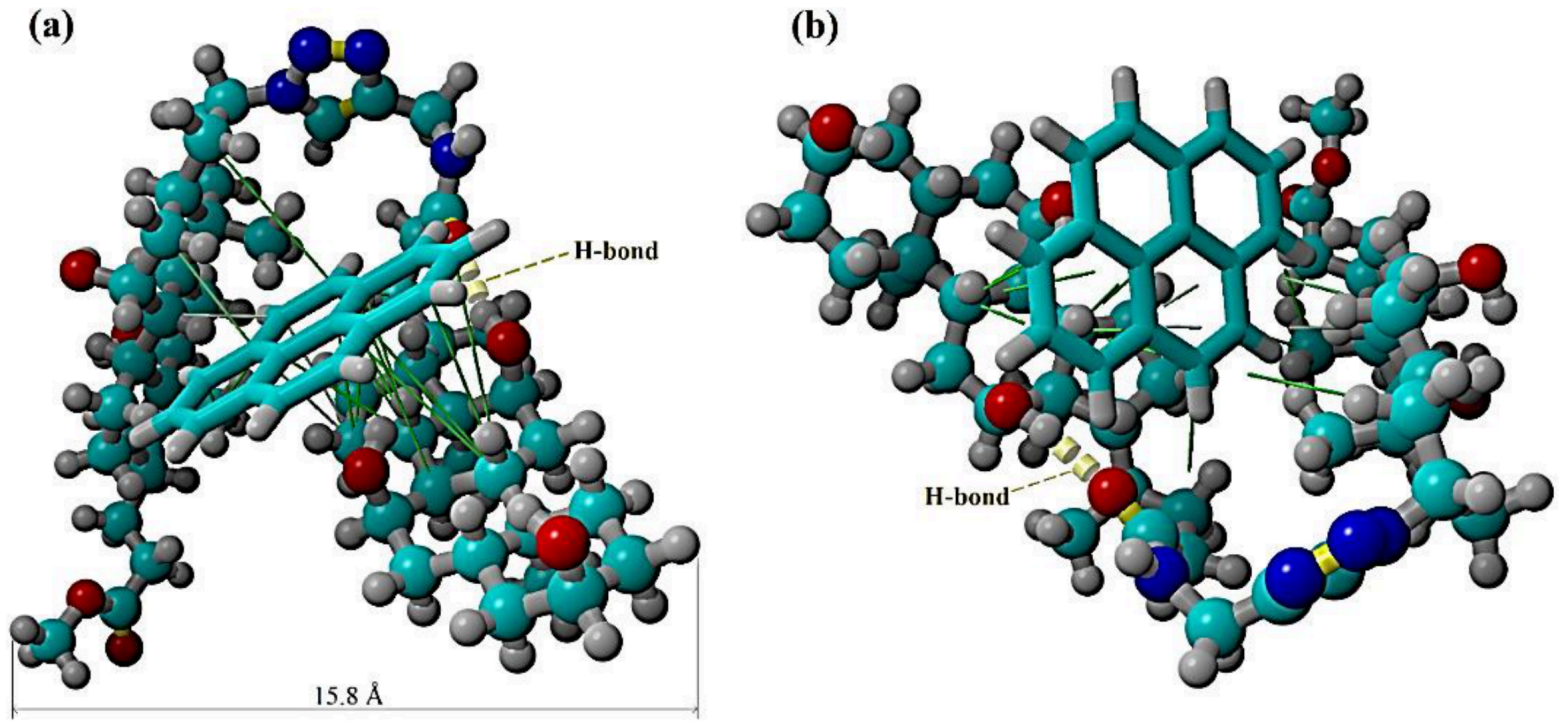
Molecular docking model showing the best-pose interaction complex host–guest (**a**) top view relative to the pyrene molecule and (**b**) side view relative to the pyrene molecule.

Commonly, the lower the values of these two parameters, the stronger the interaction between the host and guest in the complex. Generally, pyrene is used as a model probe for studying the lipophilic media due to its rigidity, neutral charge, and high hydrophobicity [18]. The stability of the dimer–pyrene complex depended on a combination of physical interactions, such as hydrophobic interactions, van der Waals and CH–π forces, together with entropic effects. The molecular docking results revealed the optimal binding pose of the guest–host interaction and revealed a distance of 3.65 Å between the geometric centers of the host and guest (Figure 21). For this docked complex, the binding energy and dissociation constant were found to be *E_b_* = −5.72 kcal/mol and *K_d_* = 64.02 μM, respectively. Computational modeling studies showed that the docked complex is stabilized by hydrophobic interactions, with an average contact distance of 4.12 ± 0.53 Å. The hydrophobic interactions, which act between nonpolar groups (e.g., -CH_3_, -CH_2_, aromatic C–H) at distances below 6 Å [51], are shown in Figure 22 as green solid lines. A previous molecular modeling study on host–guest complexes, based on other cholic acid dimers and pyrene, confirmed the sequestration of the chromophore within the lipophilic cavities of the steroidal compound [23]. The dissociation constant of the docked dimer–pyrene complex, measured by molecular modeling (K_d_ = 64.02 μM), exhibited a moderate stability in the micromolar range.

**Figure 23:**
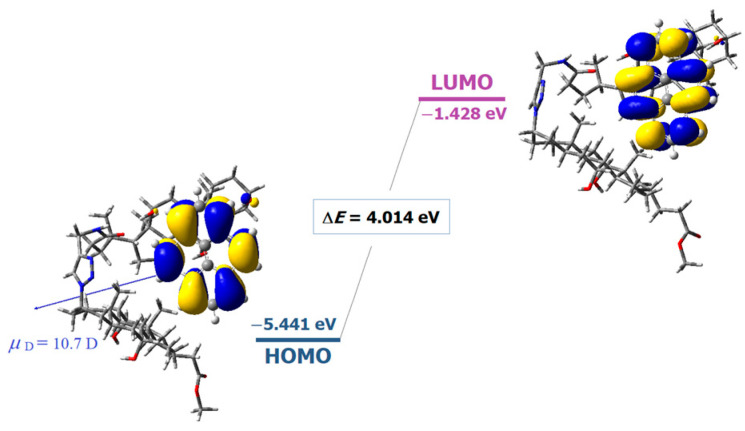
Frontier molecular orbital (HOMO and LUMO) distributions and corresponding energy levels of the dimer–pyrene complex.

Moreover, the conformation of the docked dimer–pyrene complex was further refined to account for electronic effects. To this end, the structure was optimized using Density Functional Theory (DFT) at the APFD/6-311G(2d,p) level of theory, as implemented in the Gaussian 16W program. This enabled mapping of the atomic charge distribution, revealing a significant dipole moment of 10.7 Debye for the complex. Figure 23 illustrates the energy gap between the frontier molecular orbitals (HOMO and LUMO) of the complex, which was found to be 4.014 eV.

As observed, the electron density of both HOMO and LUMO was predominantly localized on the guest molecule, suggesting that the first electronic excitation (S_0_ → S_1_) was mainly associated with a π–π* transition within the conjugated aromatic system of the guest, meaning that the dimer molecule acted as a structural framework, while the electronic and optical properties of the complex host–guest were dominated by pyrene.

**Figure 24:**
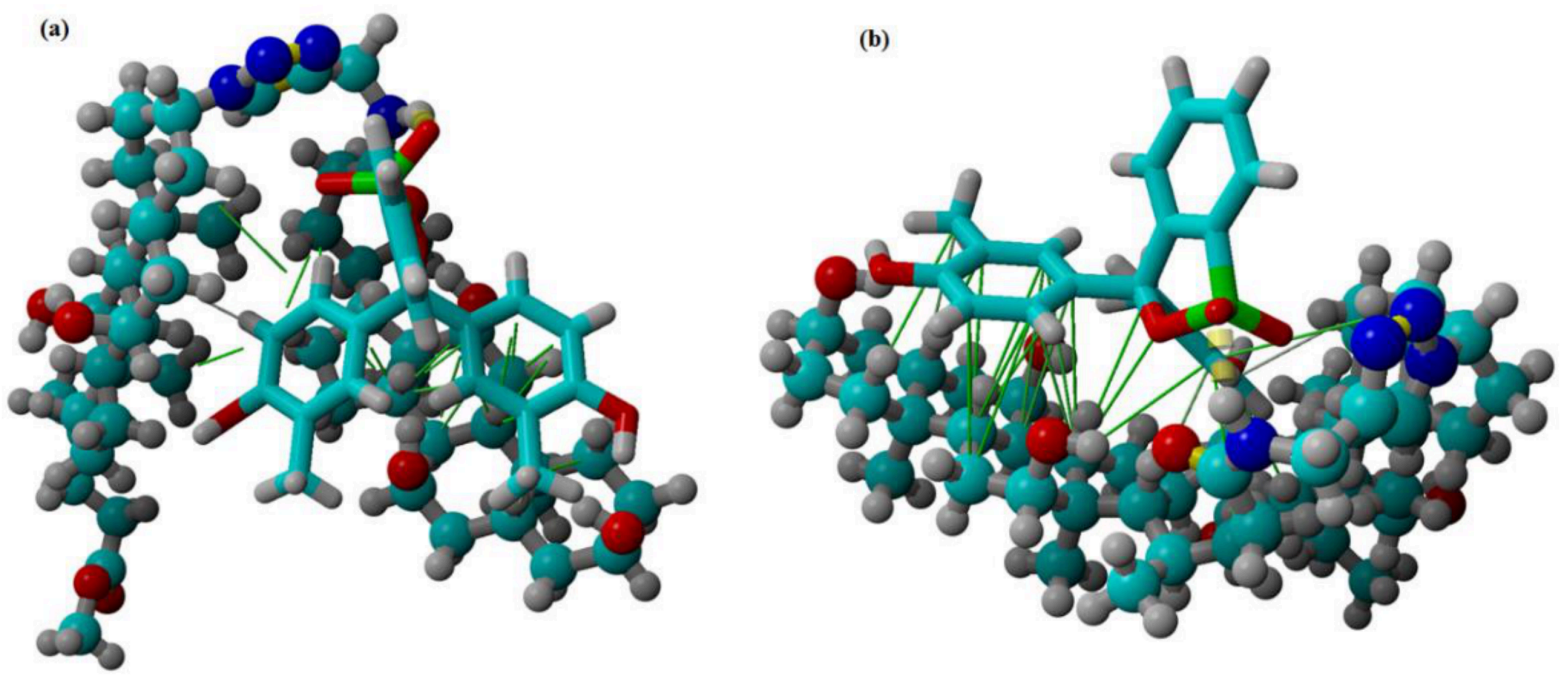
Molecular docking model of the dimer–CR complex showing: (**a**) the best-pose interaction with the sulfonate group oriented toward the polar amide group; (**b**) a detailed view of the H-bonding with the amide group.

Molecular docking simulations of the dimer–CR complex were performed and are depicted in Figure 24. For this inclusion complex, the binding energy, E_b_, was −6.39 kcal/mol, and the dissociation constant, K_d_, was 20.78 μM. These values indicated stronger interactions between the host and the guest compared to those occurring in the docked complex with pyrene, attributed to more robust forces (electrostatic interactions and hydrogen bonds) between the complex’s components.

The dissociation constant of the dimer–CR inclusion complex, measured experimentally by UV–Vis absorbance studies (K_d_ = 22.7 μM) and by molecular modeling (K_d_ = 20.78 μM), exhibited a moderate stability for the complex in the micromolar range. The strong correlation between the above-mentioned dissociation constants suggested that the force fields and parameters used in the computational model accurately reflected the electrostatic and hydrogen-bonding interactions occurring in the dimer–CR complex.

## 3. Materials and Methods

### 3.1. Materials

3α,7α,12α-trihydroxy-5β-cholan-24-oat (CA), p-toluenesulfonic acid (PTSA), triethylamine (TEA), methanesulfonyl chloride (Ms-Cl), sodium azide (NaN_3_), propargyl amine (Pr-NH_2_), 1-hydroxy benzotriazole (HO-Bt), N-(3-dimethylaminopropyl) -N′-ethylcarbodiimide hydrochloride (EDC^.^HCl), ascorbic acid, Cresol Red sodium salt (CR), pyrene, NaHCO_3_, and NaCl were purchased from Sigma-Aldrich (Sigma-Aldrich, St. Louis, MO, USA), N,N′,N′,N″,N″-pentamethyl diethylene triamine (PMDETA) was acquired from Merck (Merck KGaA, Darmstadt, Germany), while anhydrous Na_2_SO_4_ was bought from Riedel de Haën (Honeywell Riedel-de Haën, Seelze, Germany), all of them being used as received. Solvents, such as methanol, dichloromethane, dimethylformamide, ethyl acetate, and chloroform, were obtained from Honeywell Fluka (Honeywell Fluka, Seelze, Germany), and were purified/dried using standard methods. Ultrapure water, with a conductivity of 0.055 μS/cm, was obtained with a Millipore purification system (Merck Millipore, Molsheim, France) and used to prepare the aqueous solutions for the fluorescence studies.

### 3.2. Synthetic Procedures

The reaction protocols and the characterization of compounds **1**–**6** (Scheme 1 and Scheme 2), performed via 1D/2D NMR, FTIR, and ESI-MS (+), are shown below.

#### 3.2.1. 3α,7α,12α-Trihydroxy-5β-cholane-24-oate (**1**): White Solid, mp = 198–200 °C

^1^H NMR (DMSO-d_6_, 400 MHz, δ (ppm)): 0.59 (3H, s, CH_3_, H18), 0.81 (3H, s, CH_3_, H19), 0.92 (3H, d, *J* = 6.2 Hz, CH_3_, H21), 0.84–1.63 (2H, m, CH_2_, H1), 0.95–1.66 (2H, m, CH_2_, H15), 1.18–1.64 (2H, m, CH_2_, H22), 1.19–1.76 (2H, m, CH_2_, H16), 1.24 (1H, m, CH, H5), 1.26–1.46 (2H, m, CH_2_, H2), 1.29 (1H, m, CH, H20), 1.34 (1H, m, CH, H8), 1.39 (2H, m, CH_2_, H11), 1.36–1.80 (2H, m, CH_2_, H6), 1.46–2.22 (2H, m, CH_2_, H4), 1.78 (1H, m, CH, H17), 1.98 (1H, td, *J* = 12.0; 7.1 Hz, CH, H14), 2.07–2.27 (2H, m, CH_2_, H23), 2.15 (1H, m, CH, H9), 3.19 (1H, ddq, *J* = 10.6; 5.5; 4.3 Hz, CH, H3), 3.40 (bs, residual water signal from DMSO-d_6_), 3.61 (1H, t, *J* = 3.2 Hz, CH, H7), 3.78 (1H, d, *J* = 3.4 Hz, CH, H12), 4.02 (1H, d, *J* = 3.2 Hz, OH7), 4.12 (1H, d, *J* = 3.4 Hz, OH12), 4.35 (1H, bs, OH3), 11.95 (1H, bs, COOH24). ^13^C NMR: 12.33 (C18), 16.94 (C21), 22.61 (C19), 22.80 (C15), 26.2 (C9), 27.28 (C16), 28.52 (C11), 30.39 (C2), 30.79 (C22), 30.83 (C23), 34.39 (C10), 34.86 (C6), 35.05 (C20), 35.3 (C1), 39.47 (C8), 39.55 (C4), 41.36 (C14), 41.51 (C5), 45.75 (C13), 46.08 (C17), 66.26 (C7), 70.44 (C3), 71.0 (C12), 174.99 (C24).

FTIR (KBr) (cm^−1^): 3800–3080 (br, ν_O-H (C3, C7, C12 C24)_ free and H-bonded), 2966 (ν_as (CH3)_), 2938 (ν_as (CH2)_), 2872 (ν_s (CH3)_), 1716 (ν_C=O (acid)_), 1468 (δ_CH2_), 1375 (δ_s(CH3)_), 1254 (ν_C-O (acid)_), 1196 (δ_O-H (C3, C7, C12)_), 1120 (ν_(C-C)sk_), 1097 (ν_C-O (C12)_), 1078 (ν_C-O (C7)_), 1037 (ν_C-O (C3)_), ῦ < 1000 cm^−1^ (γ_O-H_ + γ_(C-H)sk_).

ESI-MS (+): *m*/*z* 431.2506 (M + 23 for Na), *m*/*z* 817.4658 (2M + 1 for H), *m*/*z* 839.4621 (2M + 23 for Na).

#### 3.2.2. Synthesis of Methyl 3α,7α,12α-Trihydroxy-5β-cholane-24-oate (**2**)

Product **2** was obtained by modifying the esterification protocol between CA and methanol [52]. The reaction protocol involved dissolving 2.50 g (6.13 mmol) of CA in 13 mL (321 mmol) of methanol, followed by the addition of 0.11 g (0.85 mmol) of PTSA. The mixture was heated to reflux for 4h, and then it was poured dropwise into crushed ice. The obtained solid was separated by filtration, washing with cold water, and drying under pressure.

Methyl 3α,7α,12α-trihydroxy-5β-cholane-24-oate (**2**): white solid, yield: 7.05 g (98%); mp = 155–156 °C;

^1^H NMR (DMSO-d_6_, 400 MHz), δ (ppm): 0.59 (3H, s, CH_3_, H18), 0.81 (3H, s, CH_3_, H19), 0.91–0.93 (3H, d, *J* = 6.2 Hz, CH_3_, H21), 0.84–2.36 (23H, m, 9 CH_2_ and 5 CH: H1, H2, H4–H6, H8, H9, H11, H15–H17, H20, H22, H23), 1.94–2.02 (1H, td, *J* = 12.0; 7.1 Hz, CH, H14), 3.19 (1H, ddq, *J* = 10.8; 8.5; 4.3 Hz, CH, H3), 3.38 (bs, residual water signal from DMSO-d_6_), 3.58 (3H, s, OCH_3_, H25), 3.61 (1H, m, CH, H7), 3.78 (1H, q, *J* = 3.4 Hz, CH, H12), 4.02 (1H, d, *J* = 3.2 Hz, OH7), 4.13 (1H, d, *J* = 3.4 Hz, OH12), 4.34 (1H, d, *J* = 4.3 Hz, OH3). ^13^C NMR: 12.29 (C18), 16.89 (C21), 22.60 (C19), 22.79 (C15), 26.19 (C9), 27.25 (C16), 28.51 (C11), 30.38 (C2), 30.46 (C23), 30.72 (C22), 34.37 (C10), 34.85 (C6), 35.02 (C20), 35.30 (C1), 39.47 (C8), 39.55 (C4), 41.36 (C14), 41.50 (C5), 45.75 (C13), 46.01 (C17), 51.18 (C25), 66.24 (C7), 70.43 (C3), 70.98 (C12), 173.83 (C24).

FTIR (KBr)(cm^−1^): 3800–3080 (br, ν_O-H (C3, C7, C12)_ free and H-bonded), 2926 (ν_as (CH3 +CH2)_), 2870 (ν_s (CH3)(ester)_), 1737 (ν_(C=O) (ester)_), 1437 (δ_as(CH3)(ester)_), 1194 (ν_as_(_C-O-C)(ester)_), ῦ < 1000 cm^−1^ (γ_O-H_ + γ_(C-H)sk_).

ESI-MS (+): *m*/*z* 445.2710 (M + 23 for Na), *m*/*z* 845.5103 (2M + 1 for H), *m*/*z* 868.2158 (2M + 23 for Na).

#### 3.2.3. Synthesis of Methyl-3α-mesyloxy-7α-12α-dihydroxy-5β-cholane-24-oate (**3**)

Product **3** was obtained by changing the protocol of the mesylation reaction between Ms-Cl and compound **2** [52]. The reaction protocol described the addition of 2 mL (14.22 mmol) of TEA to 2.00 g (4.80 mmol) of product **2**, dissolved in 20 mL of dry dichloromethane, followed by the cooling of the mixture in an ice-water bath. A chilled (0 °C) solution of 0.55 mL (7.11 mmol) of Ms-Cl, dissolved in 11 mL of dry dichloromethane, was added dropwise over 30 min to the reaction mixture. The obtained mixture was extracted with dry dichloromethane. The organic layer was washed with NaHCO_3_, water, and brine, dried over anhydrous Na_2_SO_4_, followed by distillation of the solvent and vacuum drying.

Methyl methyl-3α-mesyloxy-7α-12α-dihydroxy-5β-cholane-24-oate (**3**): white solid, yield: 1.37 g (57%); mp = 83–85 °C;

^1^H NMR (CDCl_3_, 400 MHz), δ (ppm): 0.69 (3H, s, CH_3_, H18), 0.91 (3H, s, CH_3_, H19), 0.98 (3H, d, *J* = 6.1 Hz, CH_3_, H21), 1.0–1.96 (19H, m, 8 CH_2_ and 4 CH: H1, H2, H4β–H6, H8, H11, H15–H17, H20, H22), 1.64 (bs, residual water signal from CDCl_3_, OH12, OH7), 2.15-2.41 (3H, m, CH_2_ and CH, H23 and H9), 2.60 (1H, q, *J* = 12.8 Hz, CH_2_, H4α), 2.99 (3H, s, CH_3_, H26), 3.67 (3H, s, CH_3_, H25), 3.86 (1H, s, CH, H7), 4.00 (1H, s, CH, H12), 4.53 (1H, m, CH, H3). ^13^C NMR: 12.51 (C18), 17.32 (C21), 22.30 (C19), 23.09 (C15), 26.59 (C9), 27.40 (C16), 27.89 (C2), 28.25 (C11), 30.82 (C22), 31.02 (C23), 34.12 (C6), 34.45 (C10), 34.78 (C1), 35.13 (C20), 36.05 (C4), 38.88 (C26), 39.46 (C8), 41.40 (C5), 41.87 (C14), 46.50 (C13), 47.18 (C17), 51.51 (C25), 68.11 (C7), 72.84 (C12), 82.70 (C3), 174.71 (C24).

FTIR (KBr)(cm^−1^): 3800–3080 (br, ν_O-H (C7, C12)_ free and H-bonded), 2958 (ν_as (CH3)(ester)_), 2936 (ν_as (CH2)_), 2871 (ν_s (CH2)_), 1735 (ν_C=O (ester)_), 1451 (δ_as (CH3)(ester)_), 1371 (δ_s (CH3)(mesyloxy)_), 1334 (ν_as (SO2)_), 1173 (ν_s (SO2)_), 1120 (ν_C-C_ + ν_C-O_), ῦ < 1000 cm^−1^ (γ_O-H_ + γ_(C-H)sk_).

ESI-MS (+): *m*/*z* 523.4809 (M + 23 for Na), *m*/*z* 1001.4532 (2M + 1 for H).

#### 3.2.4. Synthesis of Methyl-3β-azido-7α,12α-dihydroxy-5β-cholane-24-oate (**4**)

Product **4** was obtained by modifying the protocol of the azidation reaction between NaN_3_ and product **3** [52]. The reaction protocol involves the addition of 0.65 g (10 mmol) of NaN_3_ to 0.83 g (1.67 mmol) of compound **3**, dissolved in 20 mL of dry dimethylformamide, and stirred at 60 °C for 24 h. The reaction mixture was allowed to cool to room temperature, and the product was isolated by precipitation in ice-cold water, filtration, and drying under pressure.

Methyl-3β-azido-7α,12α-dihydroxy-5 β-cholane-24-oate (**4**)*:* white solid, yield: 0.77 g (92%); mp = 169–170 °C;

^1^H NMR (DMSO-d_6_, 400 MHz), δ (ppm): 0.60 (3H, s, CH_3_, H18), 0.86 (3H, s, CH_3_, H19), 0.92 (3H, d, *J* = 6.2 Hz, CH_3_, H21), 0.82–2.37 (22H, m, 9 CH_2_ and 5 CH: H1, H2, H4β–H6, H8, H9, H11, H15–H17, H20, H22, H23), 1.94–2.02 (1H, td, *J* = 12.0; 6.9 Hz, CH, H14), 2.63 (1H, m, CH_2_, H4α), 3.34 (s, residual water signal from DMSO-d_6_), 3.58 (3H, s, OCH_3_, H25), 3.63 (1H, t, *J* = 3.3 Hz, CH, H7), 3.79 (1H, m, CH, H12), 4.00 (1H, m, CH, H3), 4.14 (1H, d, *J* = 3.5 Hz, OH12), 4.16 (1H, d, *J* = 3.3 Hz, OH7). ^13^C NMR: 12.30 (C18), 16.90 (C21), 22.75 (C15), 22.97 (C19), 24.11 (C2), 25.75 (C9), 27.25 (C16), 28.60 (C11), 30.46 (C23), 30.52 (C1), 30.72 (C22), 32.58 (C4), 34.09 (C6), 34.73 (C10), 35.01 (C20), 37.04 (C5), 39.36 (C8), 41.38 (C14), 45.80 (C13), 46.04 (C17), 51.19 (C25), 58.36 (C3), 66.17 (C7), 70.98 (C12), 173.84 (C24).

FTIR (KBr) (cm^−1^): 3800–3080 (br, ν_O-H (C7, C12)_ free and H-bonded), 2940 (ν_as (CH3)_), 2926 (ν_as (CH2)_), 2868 (ν_s (C-H)_), 2097 (ν_as N3_), 1732 (ν_C=O (ester)_), 1453 (δ_as (CH3)_/δ_s (CH2)_), 1378 (δ_s (CH3)_), 1273-1258 (ν_C-O-C (ester)_+ δ_O-H)_), 1197 (δ_O-H (C3, C7, C12)_), 1173 (ν_(C-C)sk_), 1087 (ν_C-O (C12)_), 1074 (ν_C-O (C7)_), 1034 (ν_C-O (C3)_), ῦ < 1000 cm^−1^ (γ_O-H_ + γ_(C-H)sk_).

ESI-MS (+): *m*/*z* 448.2846 (M + 1 for H), *m*/*z* 895.5296 (2M + 1 for H).

#### 3.2.5. Synthesis of N-propargyl-3α,7α,12α-trihydroxy-5β-cholan-24-amide (**5**)

Product **5** was obtained by modifying the protocol of the propargylation reaction between Pr-NH_2_ and compound **4** [53]. The reaction protocol reported the addition of 2.12 g (11 mmol) of EDC^.^HCl and 0.57 g (3.7 mmol) of HO-Bt to a chilled solution (0 °C) of 3 g (7.3 mmol) of CA and 1.92 mL (30 mmol) of Pr-NH_2_ in 30 mL of dry dimethylformamide. Then, the reaction mixture was warmed up to room temperature and stirred for 24 h. The reaction was quenched with crushed ice, and the crude compound was extracted several times with ethyl acetate. The combined extracts were washed with water and brine, dried over anhydrous Na_2_SO_4_, followed by distillation of the solvent and vacuum drying.

N-propargyl-3α,7α,12α-trihydroxy-5β-cholan-24-amide 15 (**5**): beige solid, yield: 2.96 g (91%); mp = 276–278 °C;

^1^H NMR (DMSO-d_6_, 400 MHz), δ (ppm): 0.58 (3H, s, CH_3_, H18), 0.81 (3H, s, CH_3_, H19), 0.92 (3H, d, *J* = 6.2 Hz, CH_3_, H21), 0.84–2.26 (24H, m, 9 CH_2_ and 6 CH: H1, H2, H4–H6, H8, H9, H11, H14–H17, H20, H22, H23), 3.06 (1H, t, *J* = 2.5 Hz, CH, H28), 3.19 (1H, dq, *J* = 11.1; 5.6 Hz, CH, H3), 3.38 (bs, residual water signal from DMSO-d_6_), 3.61 (1H, m, CH, H7), 3.78 (1H, d, *J* = 3.5 Hz, CH, H12), 3.82 (2H, dd, *J* = 5.5; 2.5 Hz, CH_2_, H26), 4.02 (1H, d, *J* = 3.2 Hz, OH7), 4.11 (1H, d, *J* = 3.5 Hz, OH12), 4.35 (1H, d, *J* = 4.3 Hz, OH3), 8.22 (1H, t, *J* = 5.5 Hz, NH25). ^13^C NMR: 12.33 (C18), 17.08 (C21), 22.61 (C19), 22.79 (C15), 26.20 (C9), 27.28 (C16), 27.71 (C26), 28.54 (C11), 30.37 (C2), 31.56 (C22), 32.2 (C23), 34.37 (C10), 34.87 (C6), 35.11 (C20), 35.29 (C1), 39.47 (C8), 39.55 (C4), 41.37 (C14), 41.52 (C5), 45.73 (C13), 46.12 (C17), 66.25 (C7), 70.44 (C3), 71.02 (C12), 72.72 (C28), 81.42 (C27), 172.43 (C24).

FTIR (KBr)(cm^−1^): 3800–3080 (br, ν_O-H (C3, C7, C12) +_ ν_N-H (amide)_ overlapping, including free and H-bonded), 3298 (ν_C-H (ethynyl)_), 2933 (ν_as (CH2)_), 2856 (ν_s (CH3)_), 2123 (ν_C≡C_), 1645 (ν_C=O_, Amide I), 1535 (ν_C-N +_ δ_N-H_, Amide II), 1451 (δ_CH2_), 1378 (δ_s (CH3)_), 1196 (δ_O-H (C7, C12)_), 1077 (ν_C-O (C7)_), 1044 (ν_C-O (C3)_), ῦ < 1000 cm^−1^ (γ_O-H_ + γ_(C-H)sk_).

ESI-MS (+): *m*/*z* 468.2277 (M + 23 for Na), *m*/*z* 891.4286 (2M + 1 for H), *m*/*z* 913.4999 (2M + 23 for Na).

#### 3.2.6. Synthesis of the Dimer (**6**)

A total of 2.10 g (4 mmol) of compound **4** and 1.78 g (4 mmol) of compound **5** were dissolved separately in 25 mL of dry dimethylformamide. The two solutions were mixed, and 0.84 mL of PMDETA (4 mmol), 0.77 g (4 mmol) of CuI, and 0.72 g (4 mmol) of ascorbic acid were added to the resulting solution. The reaction mixture was stirred for 72 h at 70 °C in the dark. After that, the solution was precipitated in water, and the crude mixture, isolated by filtration and drying under pressure, was dissolved in dichloromethane and washed several times with a 0.5 M EDTA solution (Ph = 8), followed by washing with water and brine and dried over anhydrous Na_2_SO_4_. The obtained solid was purified by flash chromatography on silica gel (95/5 dichloromethane/methanol).

Dimer (**6**): beige solid, yield: 2.98 g (85%); mp = 119 = 120 °C;

^1^H NMR (DMSO-d_6_, 400 MHz), δ (ppm): 0.57–0.60 (6H, s, CH_3_, H18, H46), 0.79–0.81 (6H, s, CH_3_, H19, H47), 0.92 (6H, m, CH_3_, H21, H49), 2.96 (1H, m, CH_2_, H32α), 3.19 (1H, m, CH, H3), 3.36 (s, residual water signal from DMSO-d_6_), 3.58 (3H, s, OCH_3_, H53), 3.61 (1H, m, CH, H35), 3.65 (1H, m, CH, H7), 3.78 (2H, m, CH, H12, H40), 4.00 (1H, d, *J* = 2.7 Hz, OH35), 4.09 (1H, d, *J* = 2.9 Hz, OH40), 4.19 (1H, d, *J* = 3.3 Hz, OH7), 4.24 (1H, d, *J* = 2.9 Hz, OH12), 4.25 (2H, m, CH_2_, H26), 4.33 (1H, d, *J* = 3.3 Hz, OH3), 4.59 (1H, m, CH, H31), 8.01 (1H, bs, CH, H28), 8.23 (1H, bs, NH25). ^13^C NMR: 12.16 (C46), 12.21 (C18), 16.77 (C49), 16.98 (C21), 22.46 (C47), 22.62 (C19), 22.66 (C43), 22.78 (C15), 24.22 (C30), 25.99 (C37), 26.07 (C9), 27.10 (C44), 27.13 (C16), 28.41 (C39), 38.45 (C11), 30.26-30.58 (C2, C34, C50, C51), 31.48 (C22), 31.96 (C23), 32.34 (C32), 33.89 (C26), 34.24 (C29), 34.30 (C10), 34.75 (C38), 34.85 (C6), 35.0 (C20, C48), 35.16 (C1), 36.70 (C33), 39.45 (C8, C36), 39.49 (C4), 41.21 (C14, C42), 41.37 (C5), 45.58 (C41), 45.66 (C13), 45.91 (C45), 45.96 (C17), 51.04 (C53), 55.90 (C31), 66.05 (C35), 66.10 (C7), 70.29 (C3), 70.86 (C12, C40), 123.15 (C28), 138.16 (C27), 172.59 (C24), 173.67 (C52).

FTIR (KBr)(cm^−1^): 3800–3080 (br, ν_O-H (C3, C7, C12) +_ ν_N-H (amide)_ overlapping, including free and H-bonded), 2936 (ν_as (CH3)_), 2868 (ν_s (CH3)_), 1647 (ν_C=O_, Amide I), 1551 (ν_C-N +_ δ_N-H_, Amide II), 1463 (δ_as (CH3)_), 1437 (δ_(CH2)sk_), 1378 (δ_s (CH3)_), 1077 (ν_C-O (C7)_), 1035 (ν_C-O (C3)_), ῦ < 1000 cm^−1^ (γ_O-H_ + γ_(C-H)sk_).

ESI-MS (+): *m*/*z* 893.5550 (M + 1 for H), *m*/*z* 915.4697 (M + 23 for Na).

### 3.3. Analytical Methods

#### 3.3.1. Structural Characterization

1D NMR (^1^H, ^13^C, and DEPT135) and 2D NMR (^1^H,^1^H COSY; ^1^H,^13^C HSQC; ^1^H,^13^C HMBC) spectra were recorded at 25 °C on a Bruker Avance NEO 400 MHz spectrometer (Bruker BioSpin, Rheinstetten, Germany), operating at 400.1 MHz for ^1^H and 100.6 MHz for ^13^C nuclei. The spectrometer was equipped with a 5 mm four-nucleus direct-detection z-gradient probe. All experiments were performed using standard pulse sequences in the z-gradient version for 2D spectra, as delivered by Bruker with TopSpin software, version 4.5.0 (Bruker BioSpin GmbH, Rheinstetten, Germany). Chemical shifts are reported in δ units (ppm) and were referenced to the internal standards deuterated dimethyl sulfoxide (DMSO-d_6_) and deuterated chloroform (CDCl_3_) at 2.51 ppm and 7.26 ppm, respectively. The abbreviations for NMR data are s (singlet), d (doublet), t (triplet), q (quartet), m (multiplet), dd (doublet of doublet), td (triplet of doublet), dq (doublet of quartet), ddq (doublet of doublet of quartet), and bs (broad singlet). FTIR spectra were recorded on a Bruker Vertex 70 spectrophotometer (Bruker, Ettlingen, Germany) using KBr pellets as the matrix. Data were processed using OPUS 6.5 software, and the analysis included second-derivative spectroscopy and deconvolution with the 50% Lorentzian + 50% Gaussian curve-fitting procedure. The fitting procedure yielded the best fit of the original curve, with an error less than 0.003. ESI-MS (+) spectra were acquired using an Agilent 6500 Series Accurate-Mass Quadrupole Time-of-Flight (Q-TOF) LC/MS instrument (Agilent Technologies, Santa Clara, CA, USA). The flow rate was set at 0.1 mL/min, and the injection volume was 10 µL. The mass spectrometric conditions were as follows: positive ESI mode, fragmentor voltage 175 V, drying gas temperature 325 °C, and capillary voltage 4000 V. The ESI-MS spectra were recorded over an *m*/*z* range of 100–2000. The mass scale was calibrated using the standard calibration procedure and reference compounds provided by the manufacturer. Data acquisition and processing were performed using MassHunter Workstation Software—Data Acquisition for 6200/6500 Series, version B.07.00 (Agilent Technologies, Santa Clara, CA, USA). The Krüss M3000 semi-automatic melting point meter (Krüss Optronic GmbH, Hamburg, Germany) was used for the determination of the melting point (mp) of CA and its derivatives.

#### 3.3.2. Absorbance Studies

A stock solution of the dimer (**6**) in chloroform (3 mM) was prepared, and four samples, having concentrations ranging from 0 to 1.0 mM, were obtained by diluting it with the same solvent. A known amount (10 mg) (0.025 mmol) of CR dye was added to each solution, and the heterogeneous mixtures were stirred at room temperature for 36 h to reach equilibrium. The samples were then filtered through a PTFE filter (0.2 μm) to remove the solid dye excess. The filtrates were diluted 1:10 with methanol, and their absorbance spectra were recorded using a UV–Vis 155 spectrophotometer Specord 200 Plus (Analytic Jena, Jena, Germany). The dissolved concentration of CR dye was calculated based on the maximum absorption (λ_max_ = 426 nm) for each solution, the molar extinction coefficient of the colorant in methanol being ε_max_ = 17,500 (mol/L)^−1^ cm^−1^ [18,54,55]. The study of the stoichiometry of the dimer was as follows: a CR system by Job’s plot was performed in methanol using the CR absorption maximum at λ_max_ = 426 nm. The concentrations of the dimer and CR were the same, namely 5.95 × 10^−5^ M. The experimental test consisted of preparing a series of solutions of the complex host–guest by varying the mole fractions (χ_CR_ and χ_dimer_) while maintaining a constant total concentration (5.95 × 10^−5^ M) and a constant total volume (5 mL). The binding affinity of the dimer towards CR was evaluated through spectrophotometric titrations, with the association constant (K_a_) being calculated using the Benesi–Hildebrand method for 1:1 stoichiometry. For the titration experiment, the concentration of CR was kept constant (2.96 × 10^−5^ M), and the dimer concentration was varied from 0 to 5.33 × 10^−5^ M, maintaining a constant total volume (5 mL).

#### 3.3.3. Fluorescence Technique

The solution of pyrene was obtained by dissolving the fluorophore in methanol (0.5 mM), followed by a thousand-fold dilution in Millipore water (0.5 μM). The 5 mM stock solution of the dimer in DMF was followed by dilution in Millipore water to obtain several solutions, having concentrations between 0.028 and 0.33 mM; this procedure ensured the complete dissolution and prevented the formation of any suspension. To show the encapsulation of pyrene in the dimer’s pockets, the concentration of the fluorophore was maintained constant, and the fluorescence spectra were registered at different concentrations of the dimer, with the dimer solutions in Millipore water being added separately to a 1 mL solution of pyrene 0.5 μM. The concentration of pyrene in each sample was 0.15 μM. The obtained solutions were maintained at room temperature for 24 h to ensure complete encapsulation of the chromophore within the dimer (**6**) hydrophobic cavities. Pyrene was excited at 337 nm, with excitation and emission slit widths set at 5 nm and 3 nm, respectively. The emission spectra of the chromophore were recorded from 350 to 550 nm using a DuettaTM spectrometer (Horiba Ltd., Piscataway, NJ, USA) equipped with a CCD detector and a light source (75 Mw Xenon) using 10 mm quartz cuvettes. The emission intensities at 372 nm (I_1_) and 383 nm (I_3_) were measured and used to calculate the polarity parameter (I_1_/I_3_).

#### 3.3.4. In Silico Study

The 3D structure of the guest (pyrene) was retrieved from the PubChem database (PubChem Compound Summary for CID 31423, Pyrene. Available online: https://www.nih.gov (accessed on 14 February 2026) and subsequently optimized using the YASARA force field within the YASARA Structure program (version 20.8.23, YASARA Biosciences GmbH, Vienna, Austria) [56,57]. The 3D structure of the host (steroidal dimer) was constructed using the ChemDraw package (ChemOffice Ultra 2004, version 8.0, CambridgeSoft Corp., Cambridge, MA, USA) and then imported into YASARA Structure for conformational optimization based on energy minimization. For molecular docking modeling to assess the guest–host binding modes, the AutoDock version 4.2.6 (LGA) algorithm (The Scripps Research Institute, La Jolla, CA, USA) [58] was employed, which is included in the YASARA Structure program package (version 20.8.23, YASARA Biosciences GmbH, Vienna, Austria). The molecular docking computations were performed by considering 100 docking modes of host–guest interactions, followed by clustering analysis with an RMSD (root mean square deviation) threshold of 5.0 Å (for heavy atoms). The computer-aided molecular docking simulations were performed using a Dell Precision workstation T7910 equipped with 32 CPU threads (2 × Intel Xeon OCTA Core E5–2630).

## 4. Conclusions

A new tail-to-head steroidal dimer was synthesized from the native CA through a six-step reaction sequence. The ability of the dimer to form switchable molecular pockets in solvents of different polarity was demonstrated through UV–Vis absorption and steady-state fluorescence studies. Both experimental data and computational analysis confirmed a 1:1 stoichiometry for the dimer–CR complex, exhibiting moderate stability within the micromolar range. The same 1:1 stoichiometry and moderate stability within the micromolar range were proved for the docked dimer–pyrene complex by in silico studies. Steady-state fluorescence studies suggested that the quenching of pyrene by the dimer in aqueous solution was predominantly (91%) a dynamic or collisional process rather than the formation of a dimer–pyrene ground-state complex. The successful encapsulation of both hydrophilic and hydrophobic compounds in the convertible dimer cavities demonstrated its versatility as an adaptable nanocarrier and suggested it for specific applications, such as controlled drug delivery, chemical sensing, diagnostics, etc. The synthesis of novel trimers based on different bile acids and their evaluation as chemosensors for metal ions will be the subject of upcoming work.

## Figures and Tables

**Table 1:** ^1^H and ^13^C NMR chemical shifts (δ, ppm) for compounds **1**–**5** in DMSO-d_6_.

Compound 1					Compound 2			Compound 3			Compound 4			Compound 5		
		^1^H		^13^C	^1^H		^13^C	^1^H		^13^C	^1^H		^13^C	^1^H		^13^C
No.	Type	α	β		α	β		α	β		α	β		α	β	
1	CH_2_	1.63	0.84	35.30	1.65	0.85	35.30	1.85	1.02	34.78	1.48	1.17	30.52	1.65	0.85	35.29
2	CH_2_	1.26	1.46	30.39	1.27	1.44	30.38	1.70	1.86	27.89	1.41	1.66	24.11	1.28	1.44	30.37
3	CH	3.19 *		70.44	3.19		70.43	4.53		82.70	4.00		58.36	3.19		70.44
4	CH_2_	2.22	1.46	39.55	2.22	1.46	39.55	2.65	1.87	36.05	2.63	1.42	32.58	2.22	1.46	39.55
5	CH	1.24		41.51	1.24		41.50	1.46		41.40	1.48		37.04	1.24		41.52
6	CH_2_	1.80	1.36	34.86	1.80	1.36	34.85	1.94	1.52	34.12	1.86	1.35	34.09	1.80	1.36	34.87
7	CH	3.61 *		66.26	3.61		66.24	3.86		68.11	3.63		66.17	3.61		66.25
8	CH	1.34		39.47	1.34		39.47	1.51		39.46	1.38		39.36	1.34		39.47
9	CH	2.15		26.20	2.15		26.19	2.18		26.59	2.12		25.75	2.15		26.20
10	C	-		34.39	-		34.37	-		34.45	-		34.73	-		34.37
11	CH_2_	1.39		28.52	1.39		28.51	1.57		28.25	1.42		28.60	1.40		28.54
12	CH	3.78 *		71.00	3.78		70.98	4.00		72.84	3.79		70.98	3.78		71.02
13	C	-		45.75	-		45.75	-		46.50	-		45.80	-		45.73
14	CH	1.98		41.36	1.98		41.36	1.91		41.87	1.98		41.38	1.98		41.37
15	CH_2_	1.66	0.95	22.80	1.65	0.96	22.79	1.67	1.14	23.09	1.66	0.97	22.75	1.65	0.96	22.79
16	CH_2_	1.76	1.19	27.28	1.74	1.16	27.25	1.90	1.31	27.40	1.74	1.18	27.25	1.75	1.16	27.28
17	CH	1.78		46.08	1.79		46.01	1.75		47.18	1.79		46.04	1.77		46.12
18	CH_3_	0.59 *		12.33	0.59		12.29	0.69		12.51	0.60		12.30	0.58		12.33
19	CH_3_	0.81 *		22.61	0.81		22.60	0.91		22.30	0.86		22.97	0.81		22.61
20	CH	1.29		35.05	1.29		35.02	1.40		35.13	1.29		35.01	1.28		35.11
21	CH_3_	0.92 *		16.94	0.93		16.89	0.98		17.32	0.92		16.90	0.92		17.08
22	CH_2_	1.18	1.64	30.79	1.24	1.68	30.72	1.35	1.80	30.82	1.24	1.68	30.72	1.18	1.64	31.56
23	CH_2_	2.27	2.07	30.83	2.32	2.21	30.46	2.36	2.23	31.02	2.32	2.21	30.46	2.10	1.99	32.20
24	C	-		174.99	-		173.83	-		174.71	-		173.84	-		172.43
25	CH_3_/NH	-		-	3.58		51.18	3.67		51.51	3.58		51.19	8.22		-
26	CH_3_/CH_2_	-		-	-		-	2.99		38.88	-		-	3.82		27.71
27	C	-		-	-		-	-		-	-		-	-		81.42
28	CH	-		-	-		-	-		-	-		-	3.06		72.72
	OH3	4.35 *		-	4.34		-	-		-	-		-	4.35		-
	OH7	4.02 *		-	4.02		-	1.64		-	4.16		-	4.02		-
	OH12	4.12 *		-	4.13		-	1.64		-	4.14		-	4.11		-
	OH24	11.95 *		-	-		-	-		-	-		-	-		-

* Chemical shift values shown in blue were previously reported in the literature.

## Data Availability

Data are contained within the article.

## Supplementary Materials

The following supporting information can be downloaded at: https://www.mdpi.com/article/10.3390/ijms27114765/s1.
