# Supplementary material for: An Adaptive Cholic Acid Dimer for Selective Encapsulation
Source: Int J Mol Sci. 2026 May 25;27(11):4765. doi: 10.3390/ijms27114765 (PMC13257168; doi:10.3390/ijms27114765)
Supplement: Supplementary file 1 [file ijms-27-04765-s001.zip › ijms-4257642-supplementary.pdf]

Supplementary Material

An Adaptive Cholic Acid Dimer for Selective Encapsulation

Magdalena-Cristina Stanciu [1,*], Gabriela-Liliana Ailiesei [1], Mirela-Fernanda Zaltariov [1], Corneliu Cojocaru [1], Carmen Gherasim [1], Sofia-Maria Ciocan [2] and Marcela Mihai [1]

[1] "Petru Poni" Institute of Macromolecular Chemistry, 41A, Grigore Ghica Voda Alley, 700487 Iasi, Romania; gdarvaru@icmpp.ro (G.-L.A.); zaltariov.mirela@icmpp.ro (M.-F.Z.); cojocaru.corneliu@icmpp.ro (C.C.); gherasim.carmen@icmpp.ro (C.G.); marcela.mihai@icmpp.ro (M.M.)
[2] "National College", 4, Arcu Street, 700125 Iasi, Romania; ciocansofiamaria@gmail.com
* Correspondence: cstanciu@icmpp.ro

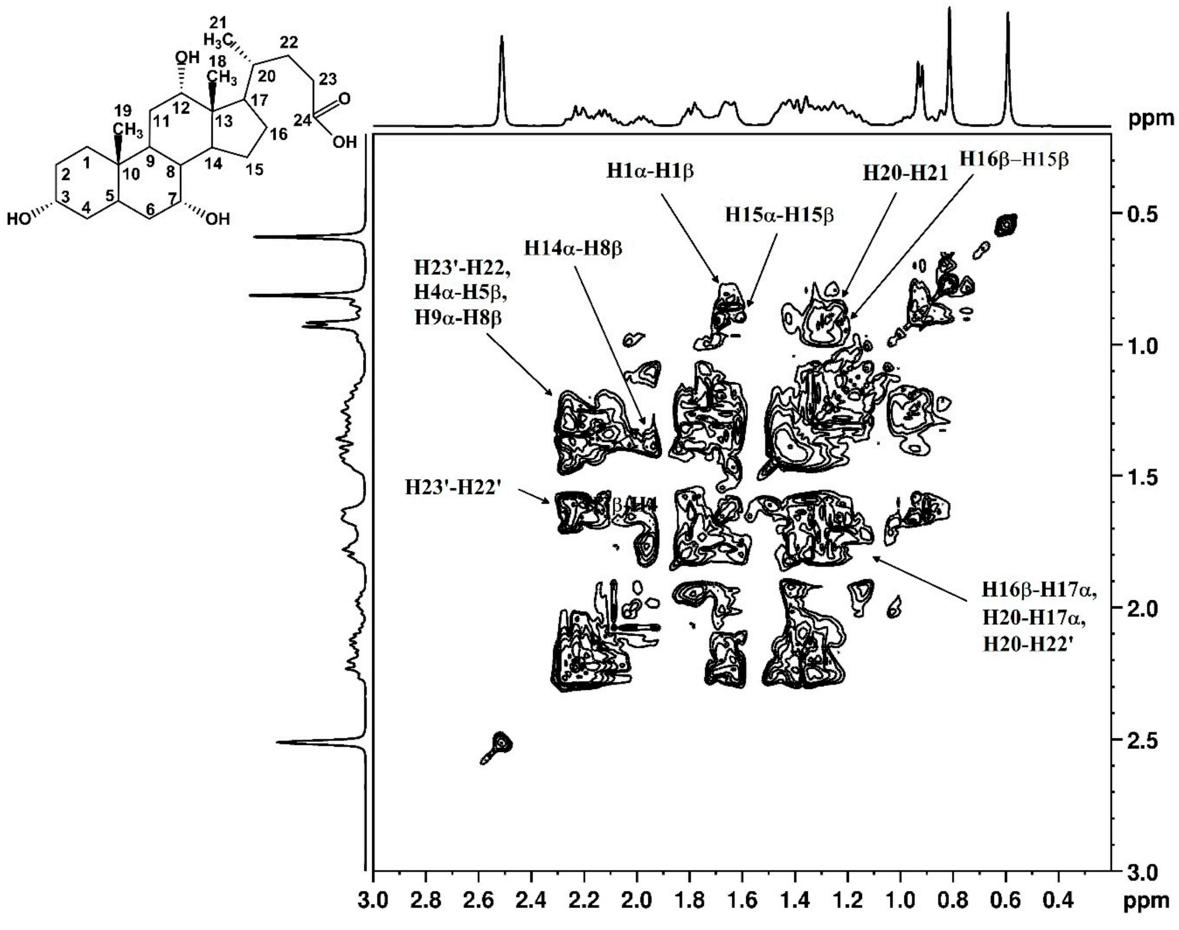

Figure S1. An enlarged view of the 0.8–2.4 ppm region of the COSY spectrum of CA.

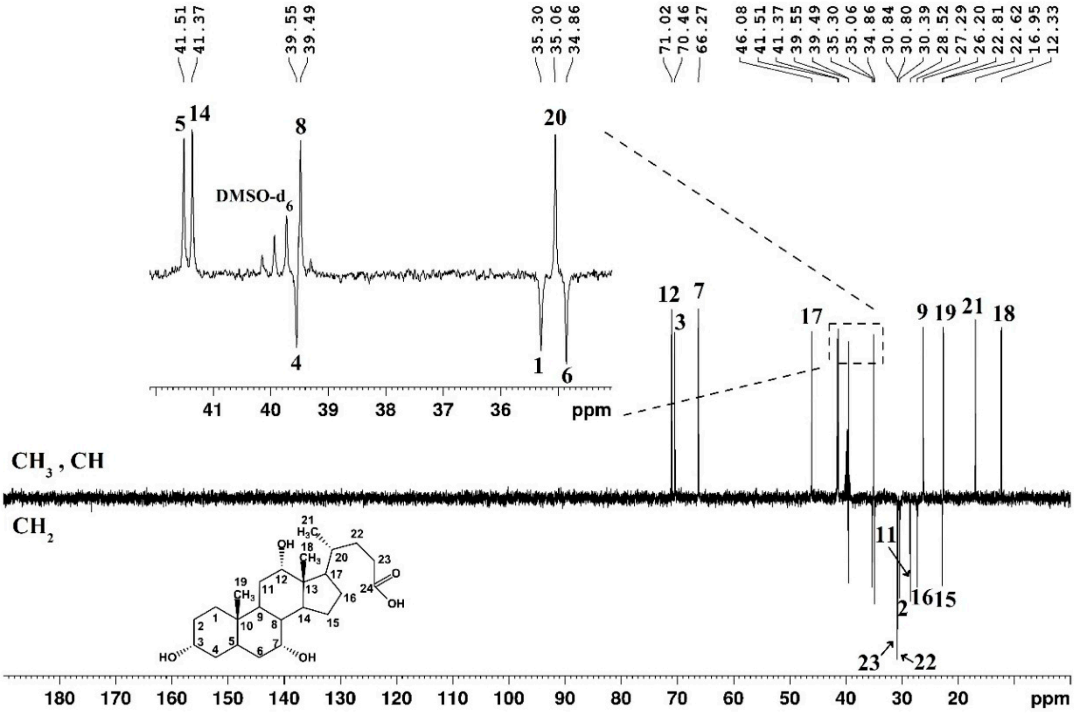

Figure S2. The DEPT135 NMR spectrum of compound **1.**

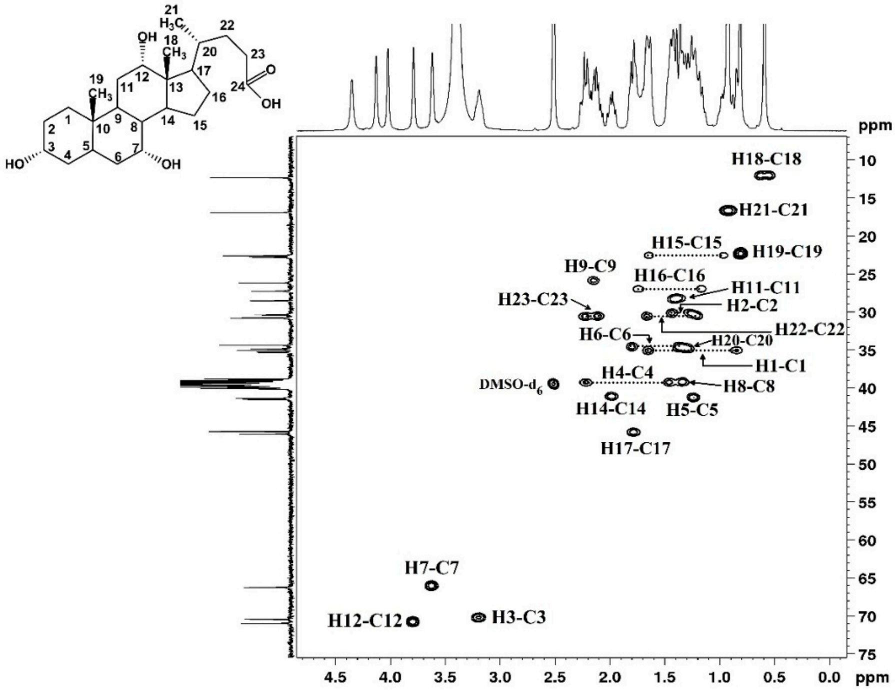

Figure S3. The ^{1}H,^{13}C HSQC spectrum of compound **1.**

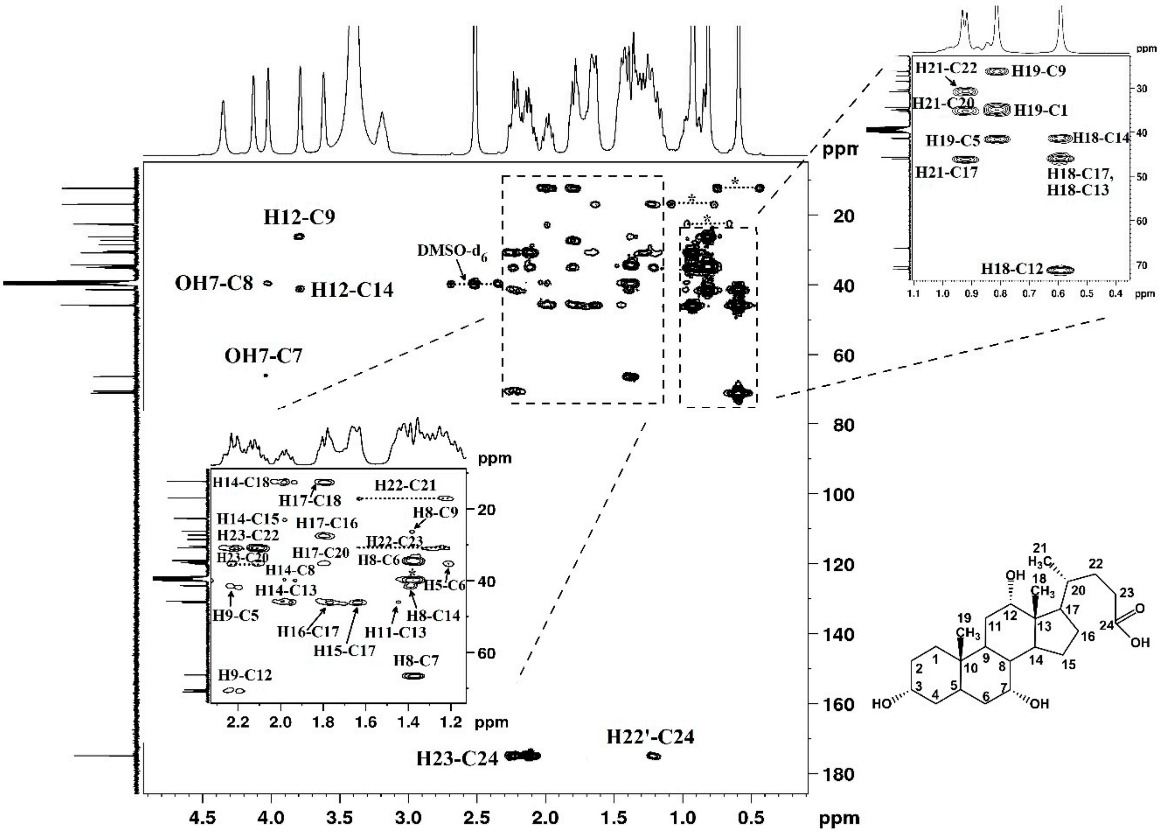

Signals marked with an asterisk (*) represented one-bond (1*J*) correlations, appearing as doublets at the ^{13}C satellite positions of the corresponding proton signals

Figure S4. The ^{1}H,^{13}C HMBC spectrum of compound **1.**

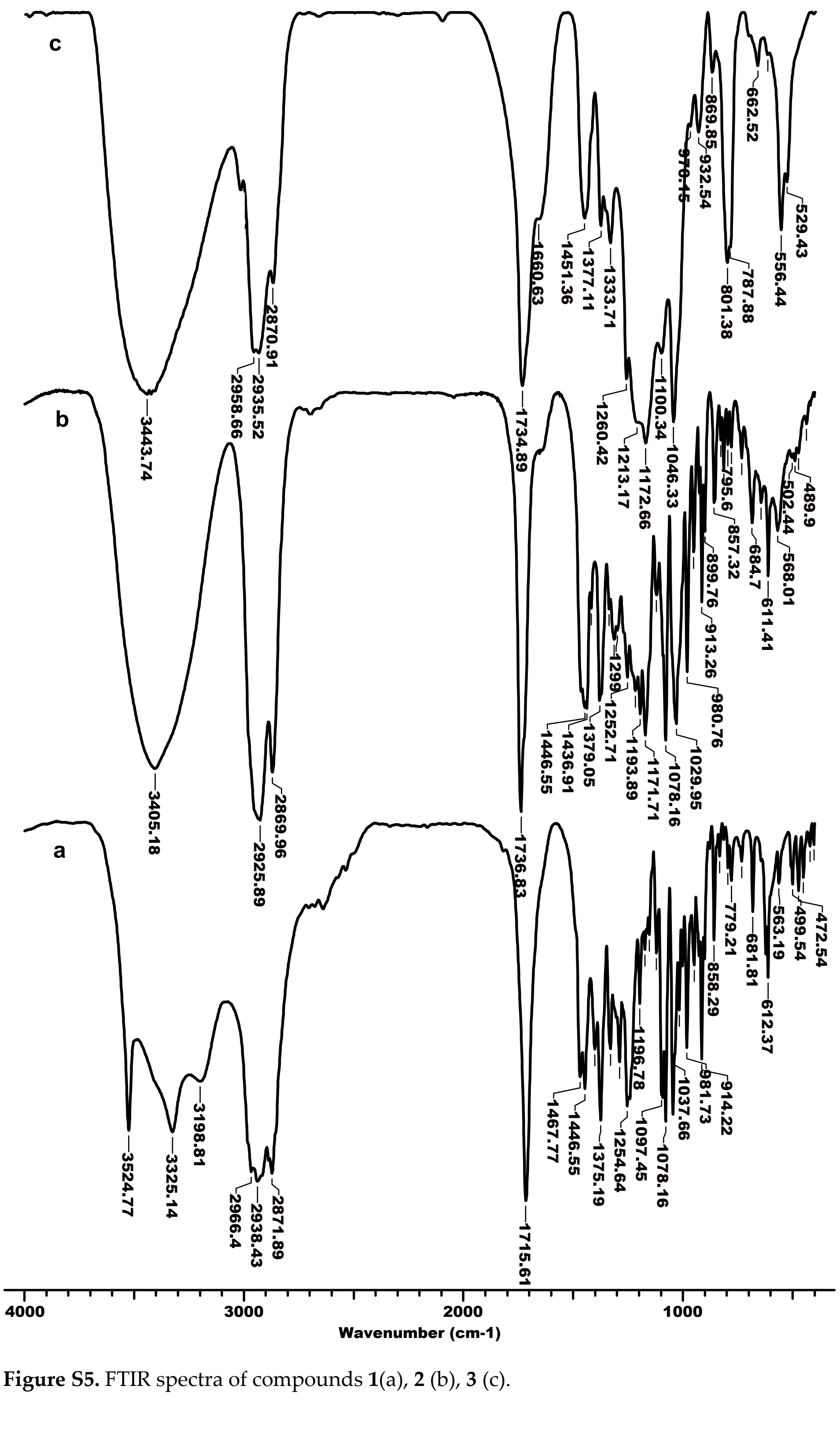

Figure S5. FTIR spectra of compounds **1**(a), **2** (b), **3** (c).

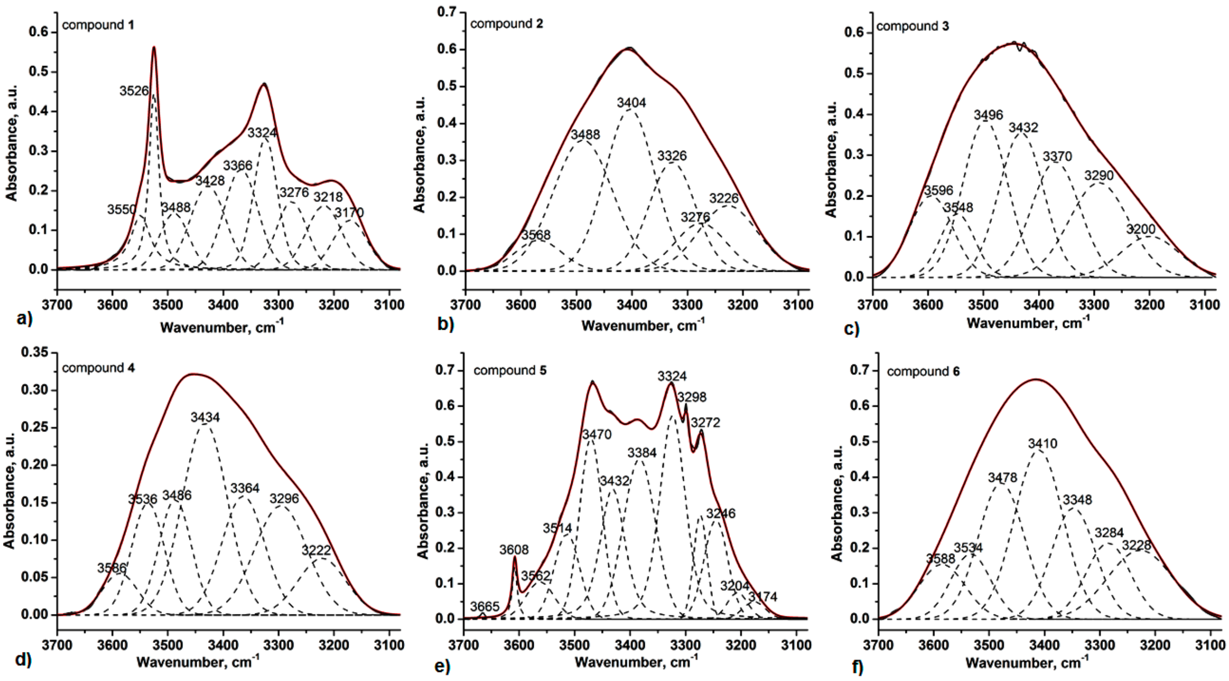

Figure S6. The deconvoluted FTIR spectra in the 3800-3080 cm^{-1} spectral region of the compounds **1-6.**

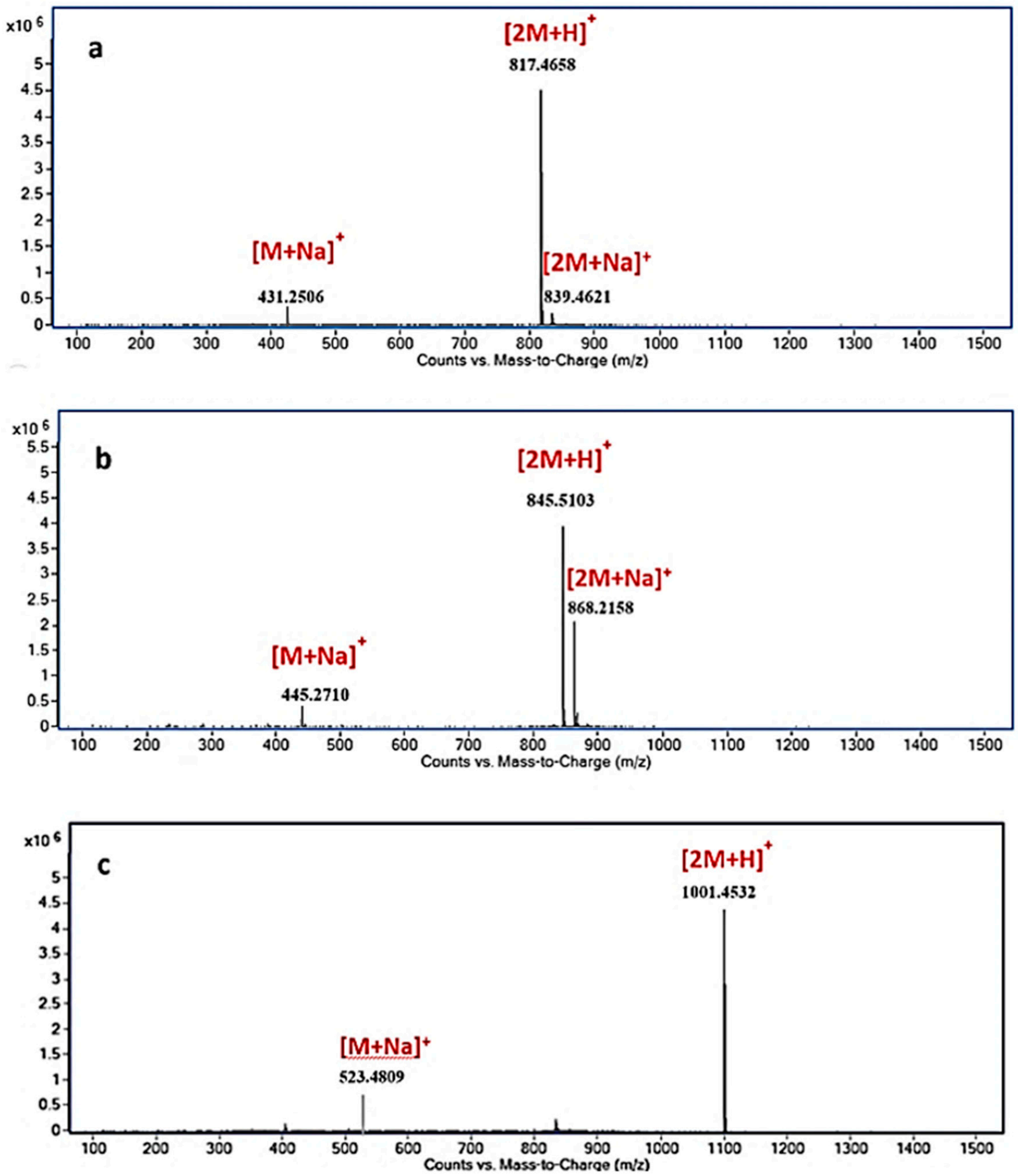

Figure S7. ESI-MS spectra of compounds **1** (a), **2** (b), **3** (c).

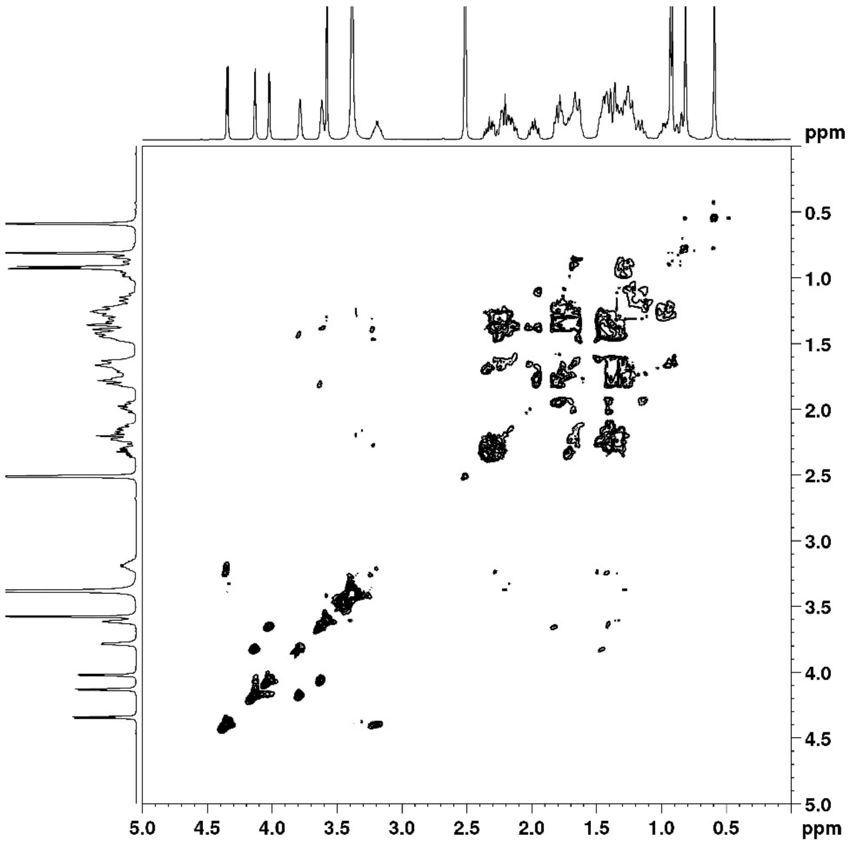

Figure S8. The $^{1}H,^{1}H$ COSY spectrum of compound **2.**

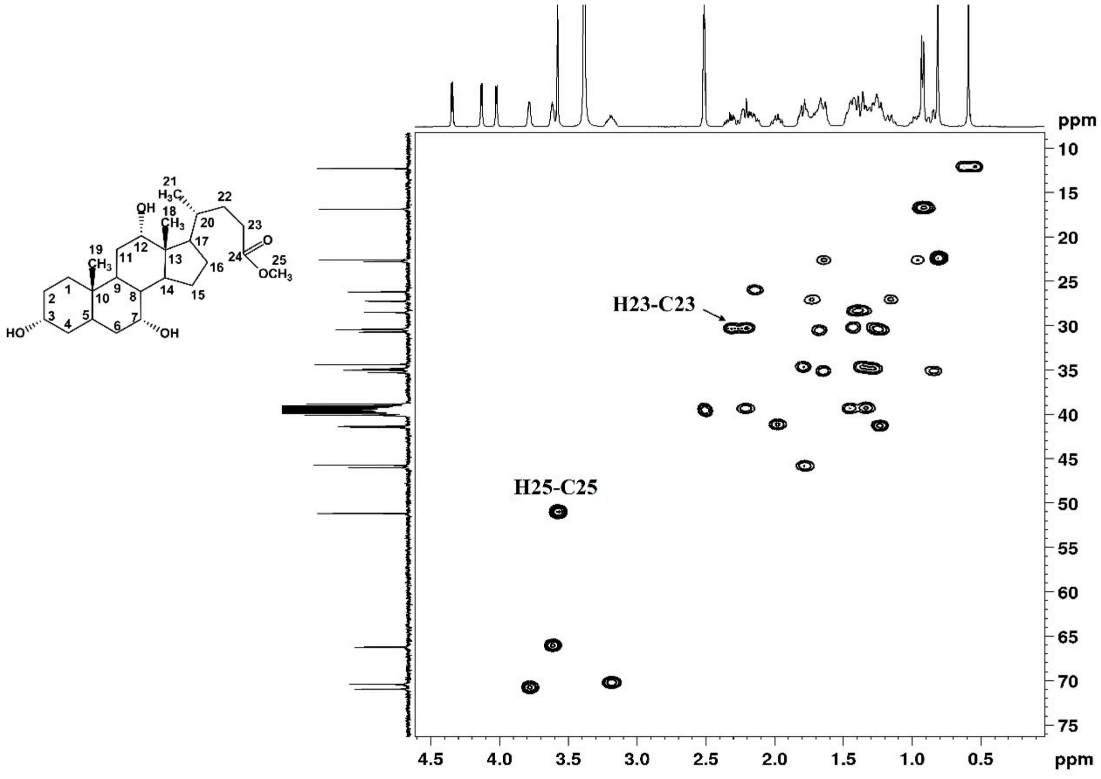

Figure S9. The $^{1}H,^{13}C$ HSQC spectrum of compound **2.**

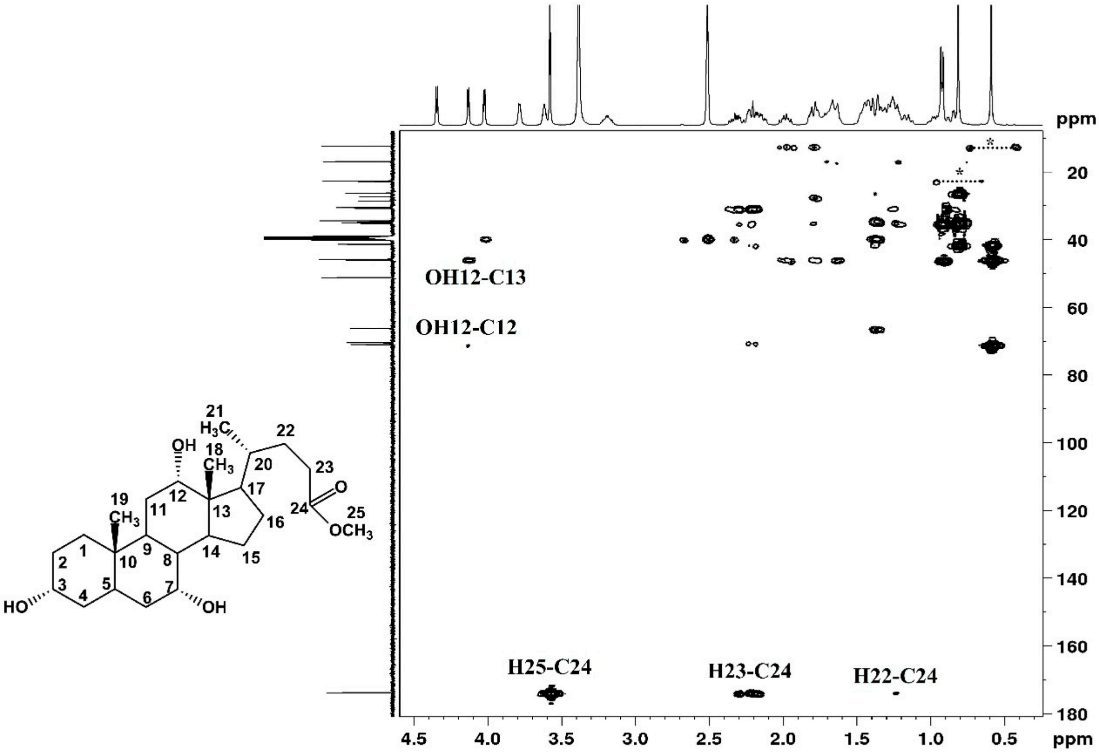

Figure S10. The $^{1}H,^{13}C$ HMBC spectrum of compound **2.**

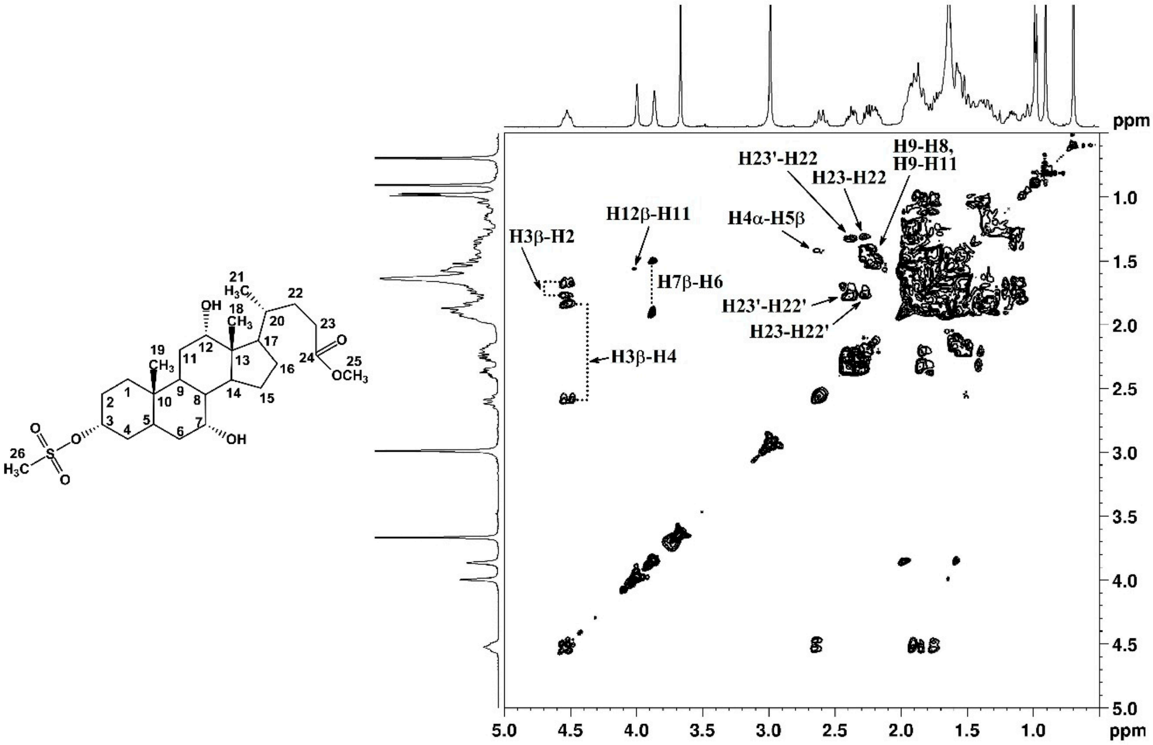

Figure S11. The $^{1}H,^{13}H$ COSY spectrum of compound **3.**

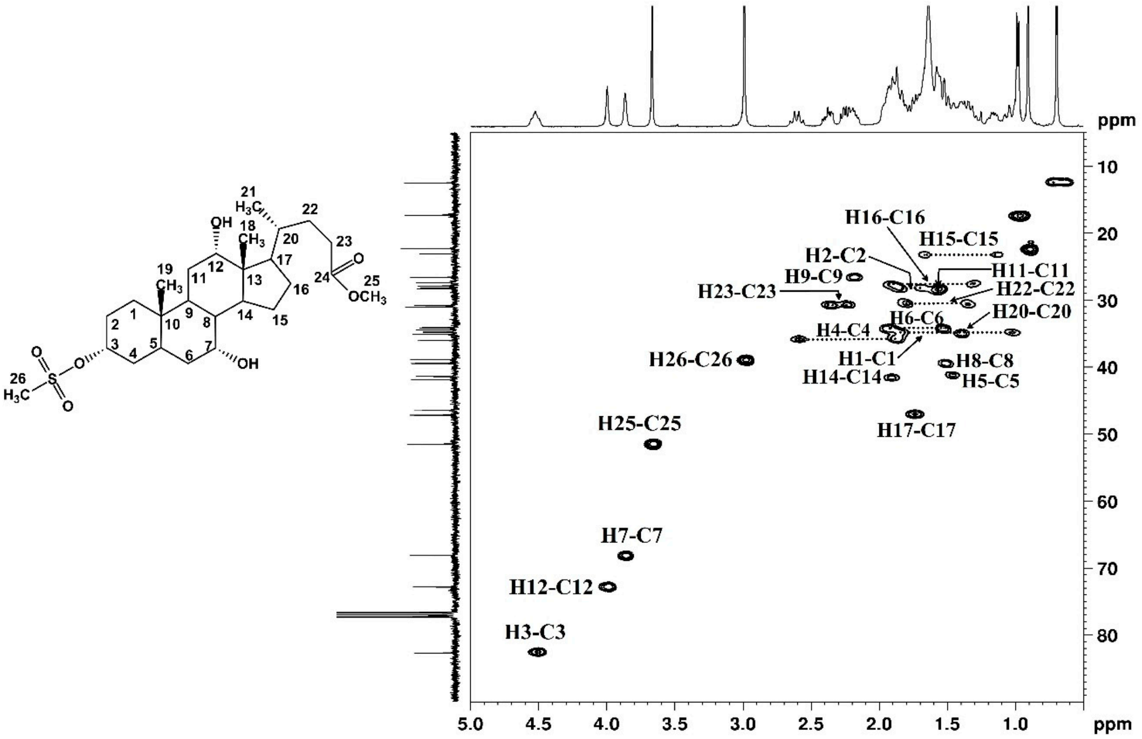

Figure S12. The ^{1}H,^{13}C HSQC spectrum of compound **3**.

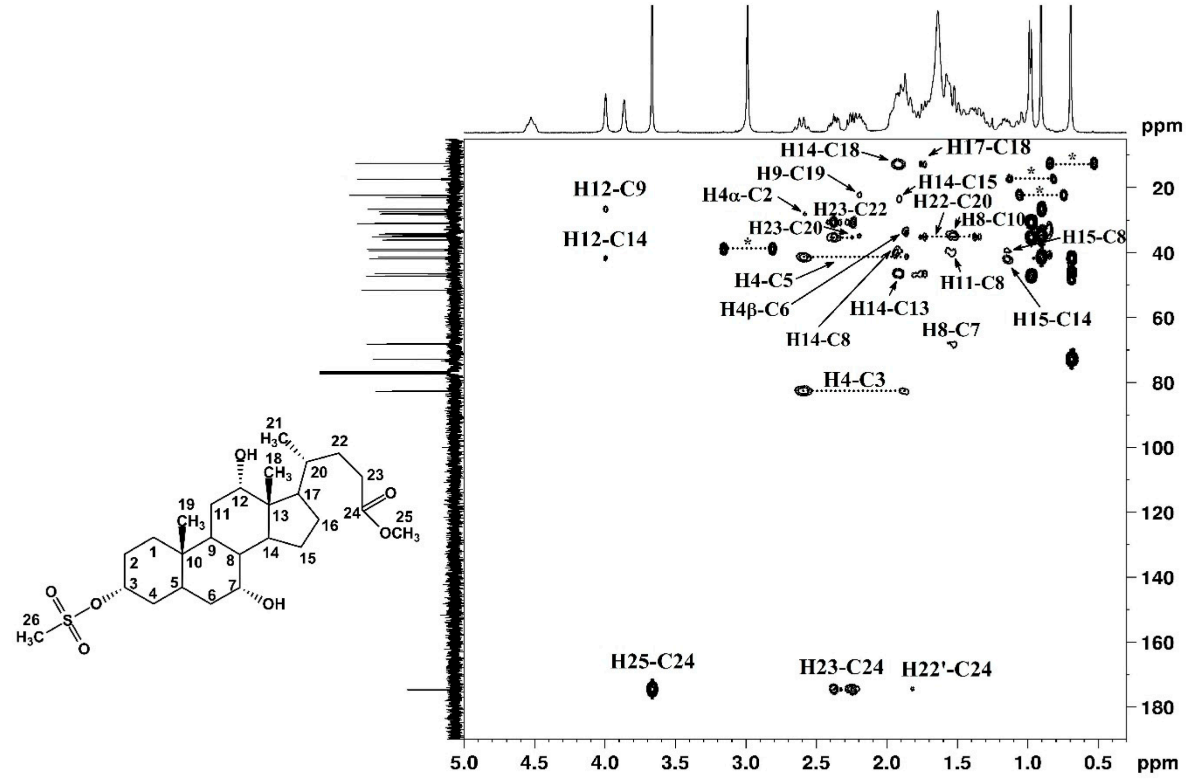

Figure S13. The ^{1}H,^{13}C HMBC spectrum of compound **3**.

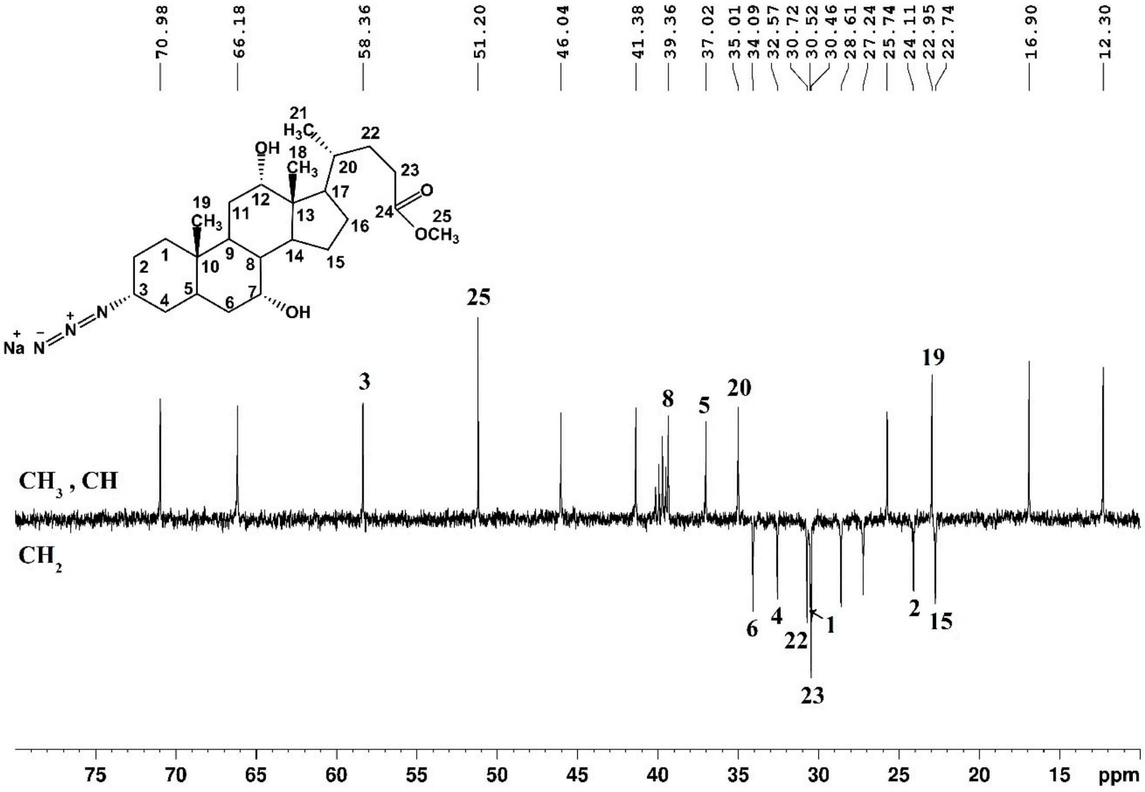

Figure S14. The DEPT135 spectrum of compound **4.**

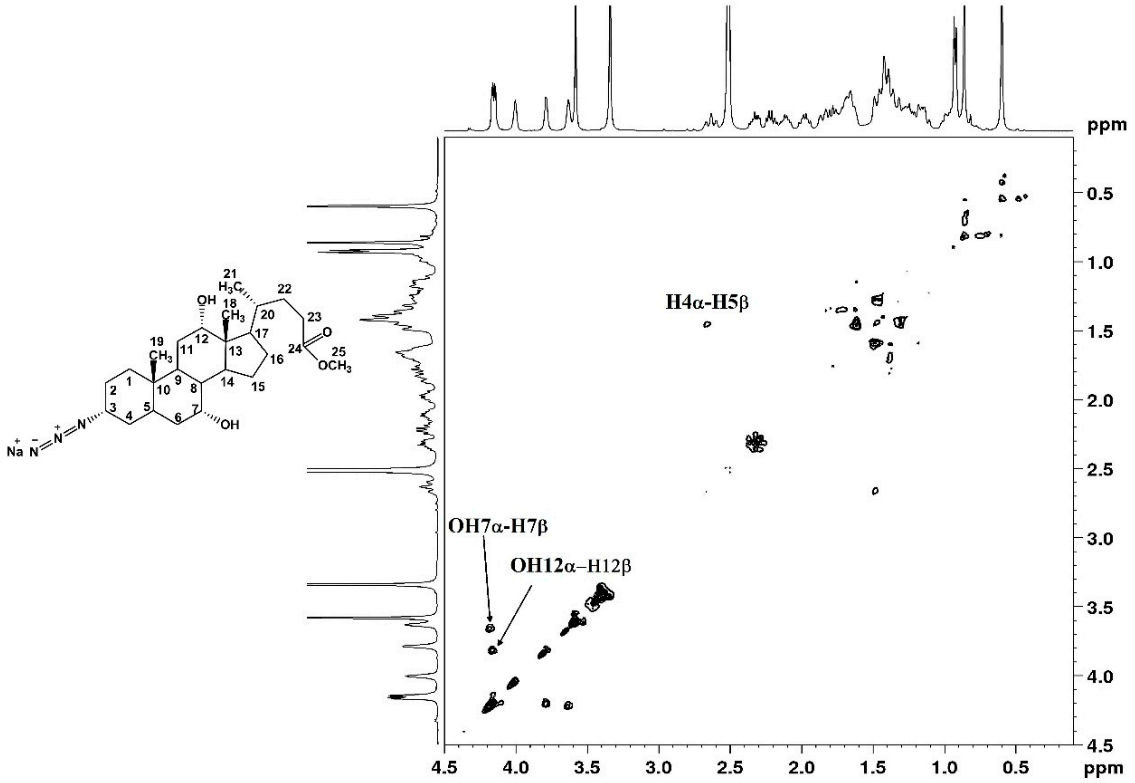

Figure S15. The $^1H,^1H$ COSY spectrum of compound **4.**

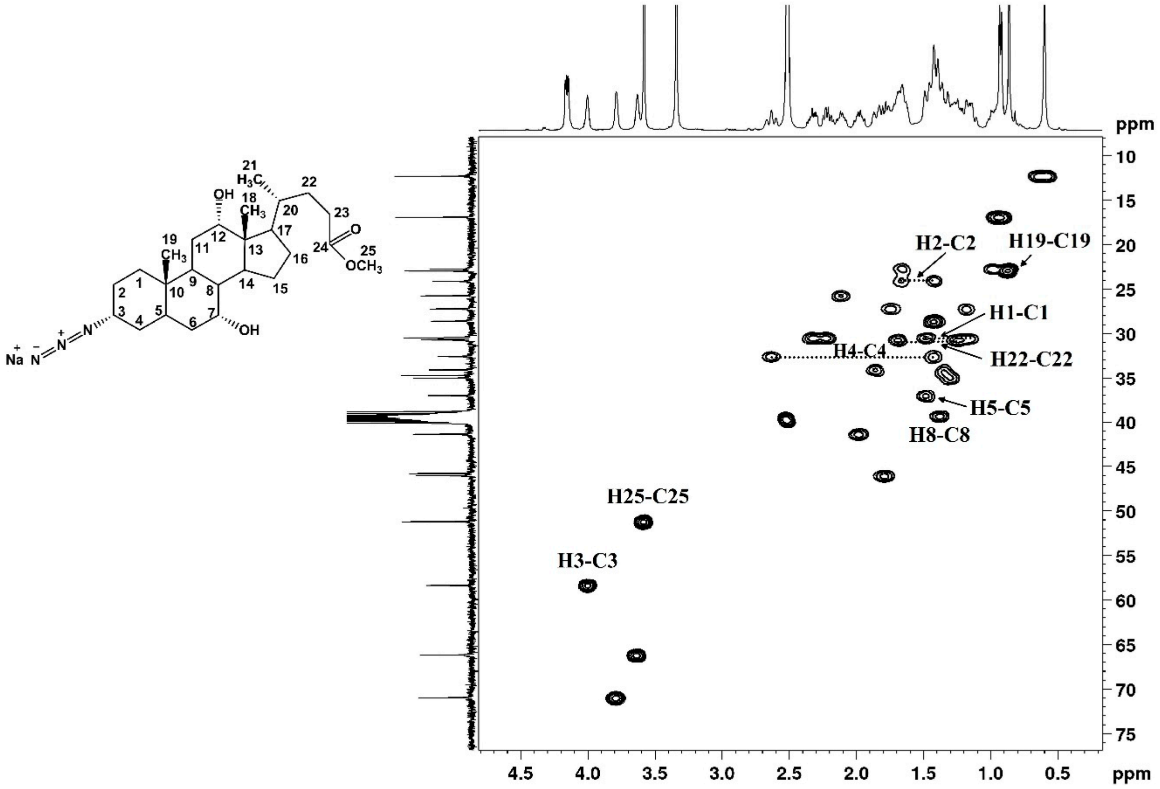

Figure S16. The $^{1}H,^{13}C$ HSQC spectrum of compound **4.**

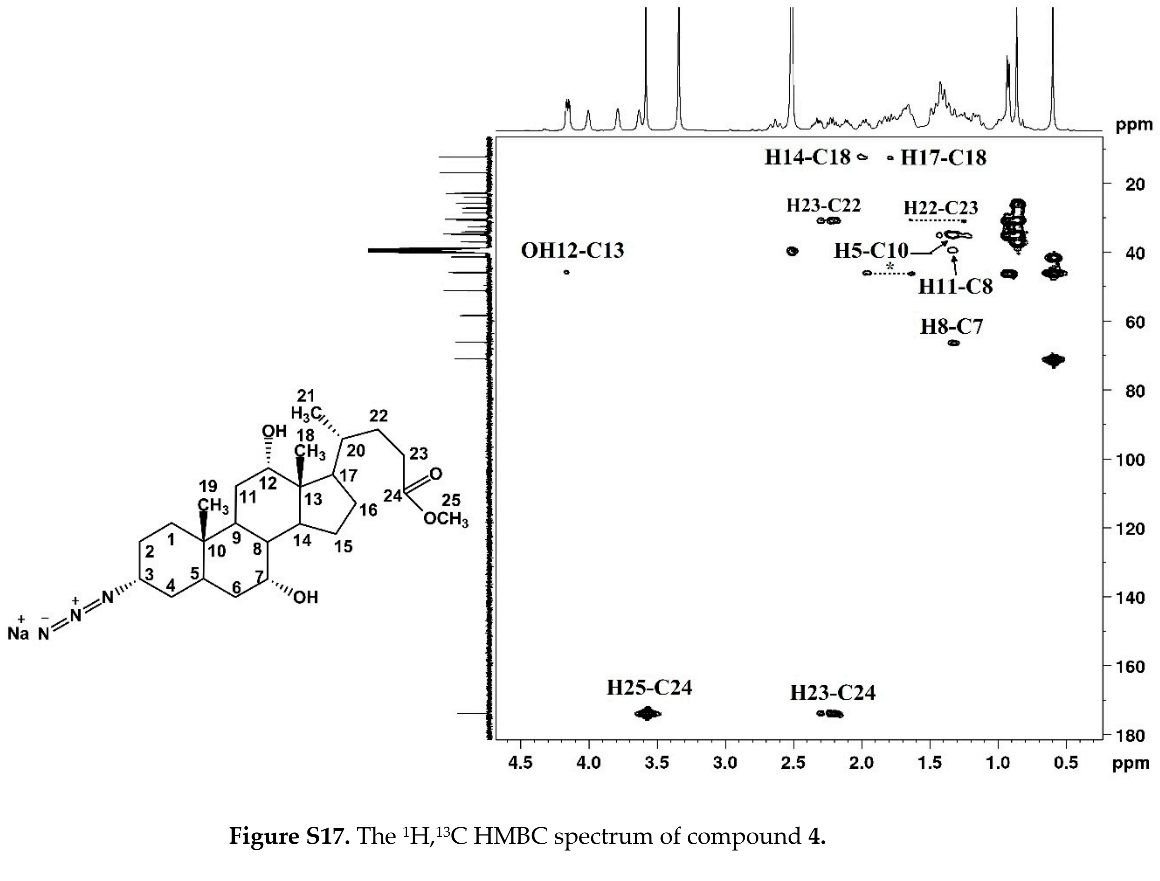

Figure S17. The $^{1}H,^{13}C$ HMBC spectrum of compound **4.**

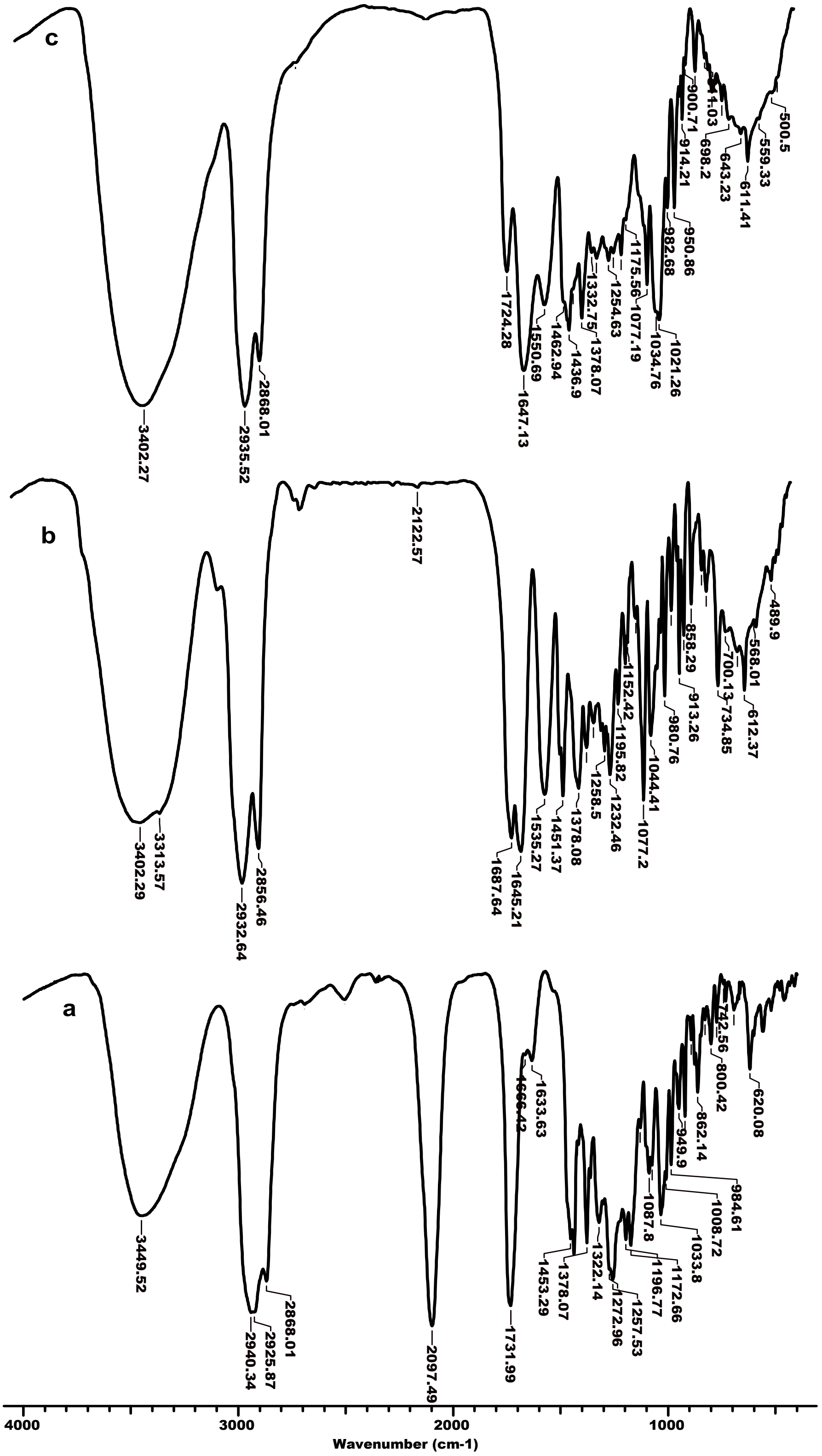

Figure S18. FTIR spectra of compounds **4** (a), **5** (b), **6** (c).

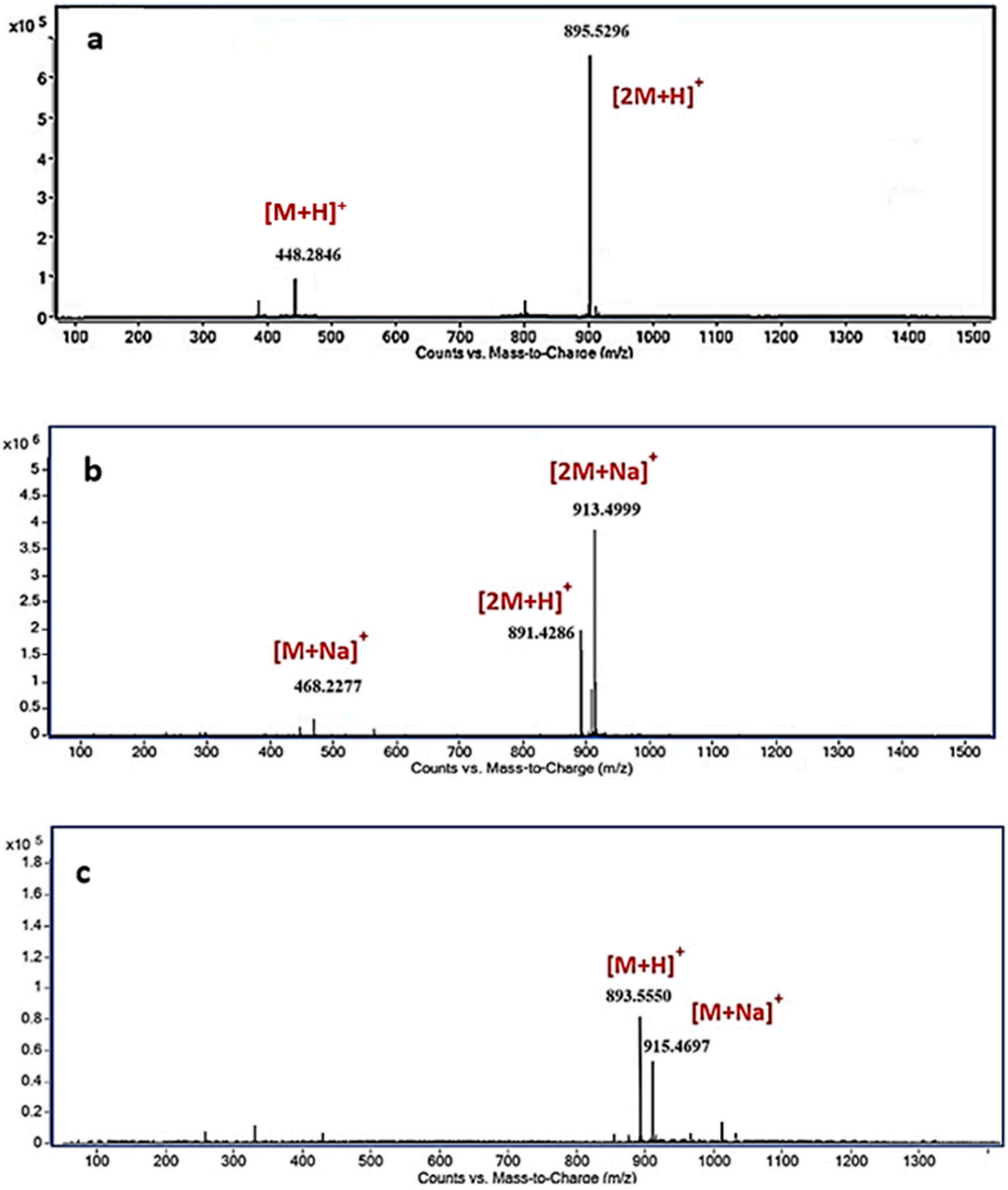

Figure S19. ESI-MS spectra of compounds **4** (a), **5** (b), **6** (c).

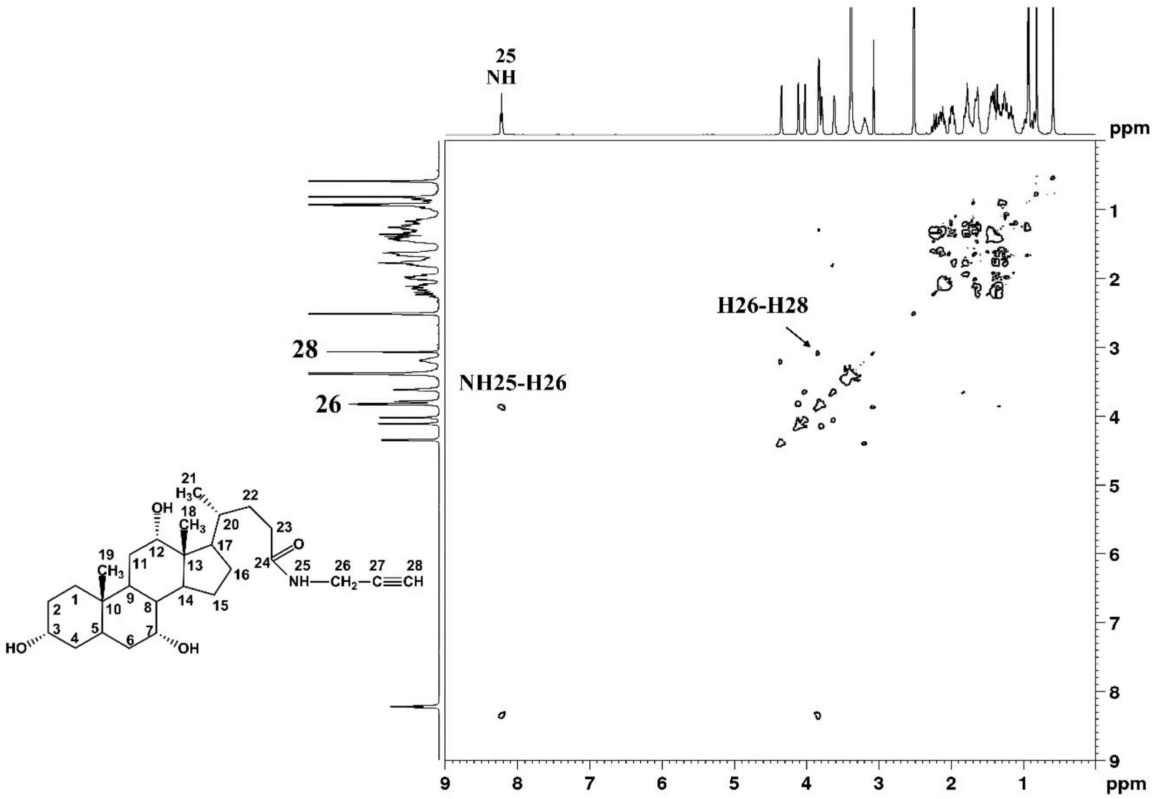

Figure S20. The ^{1}H,^{1}H COSY spectrum of compound **5**.

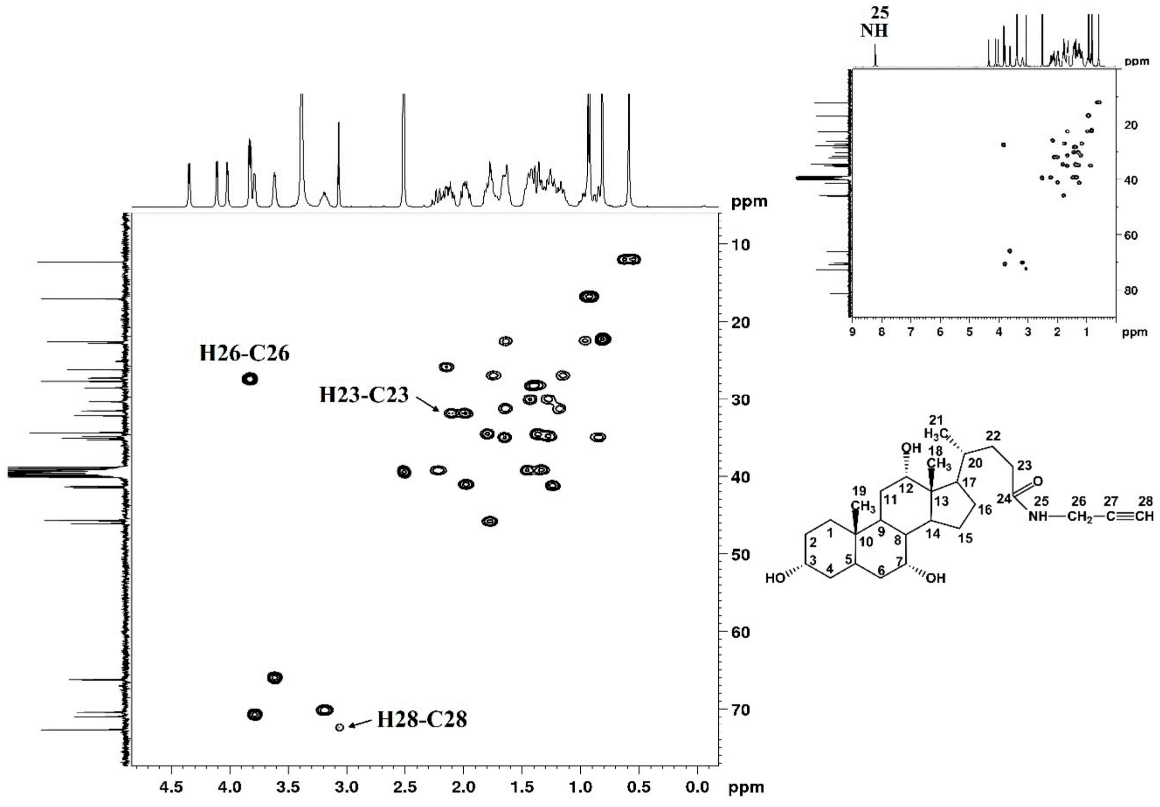

Figure S21. The ^{1}H,^{13}C HSQC spectrum of compound **5**.

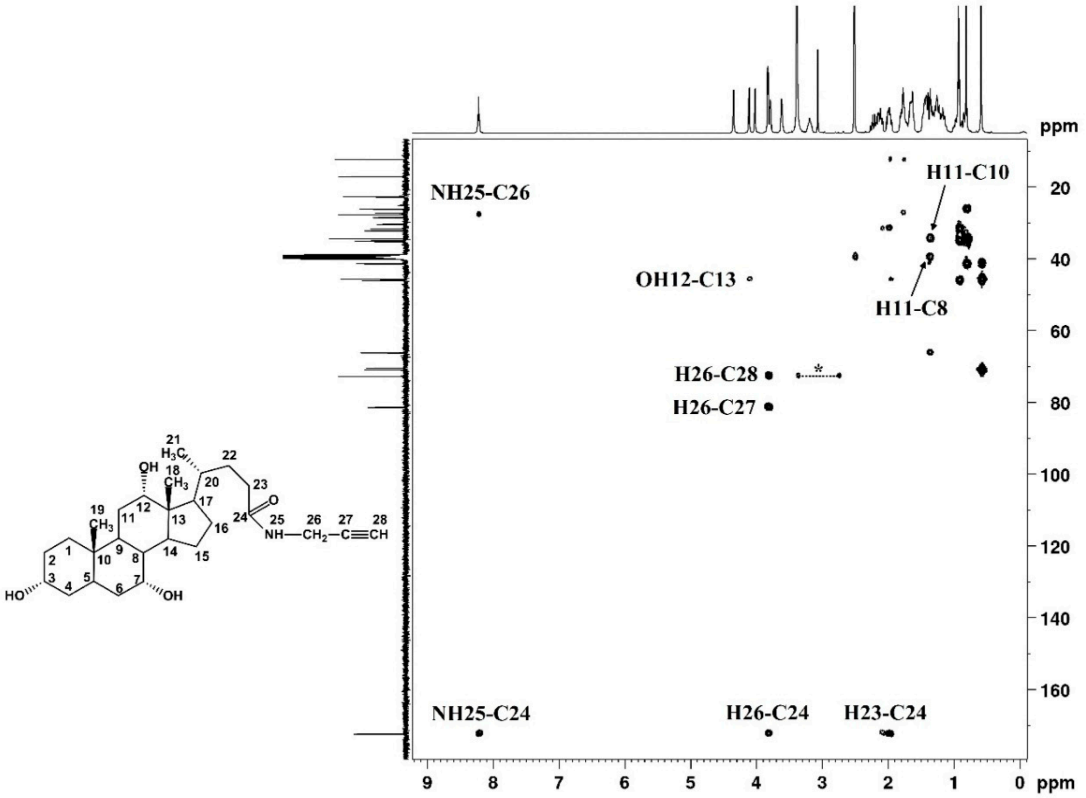

Figure S22. The ^{1}H,^{13}C HMBC spectrum of compound **5.**

Table S1. The experimental data used in Job's plot of the dimer: CR system.

**Sample**	χ_{CR}	χ_{dimer}	**V_{total} = 5 mL**		**$\Delta A \times \chi_{CR}$**
			**V_{CR} (mL)**	**V_{dimer} (mL)**	
1	0	1	0	5	0
2	0.1	0.9	0.5	4.5	0.1074
3	0.2	0.9	1	4	0.1926
4	0.3	0.7	1.5	3.5	0.2505
5	0.4	0.6	2	3	0.2740
6	0.5	0.5	2.5	2.5	0.2850
7	0.6	0.4	3	2	0.2699
8	0.7	0.3	3.5	1.5	0.2377
9	0.8	0.2	4	1	0.1827
10	0.9	0.1	4.5	0.5	0.08193
11	1	0	5	0	0

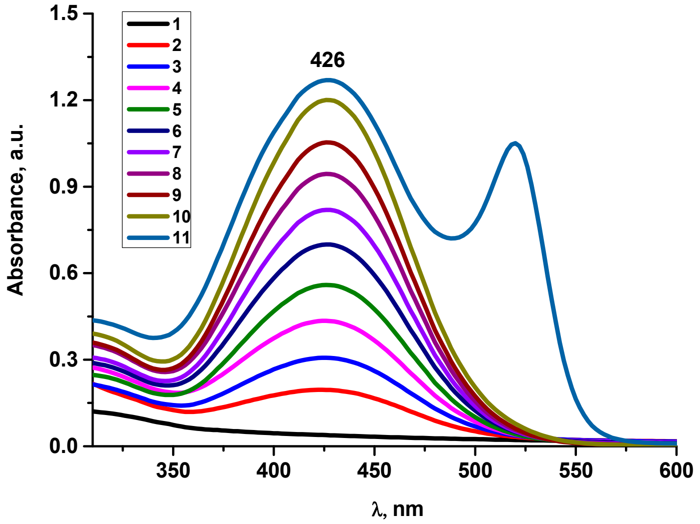

Figure S23. The UV-Vis spectra of dimer :CR complex by varying the mole fractions of both components.

Table S2. The main parameters of the Benesi-Hildebrand model.

No	[dimer]	[CR]	K_a (M^{-1})
1	0	2.96 ×10^{-5}	44 × 10^3
2	3.55 ×10^{-5}	2.37 ×10^{-5}	
3	4.15 ×10^{-5}	1.78 ×10^{-5}	
4	4.75 ×10^{-5}	1.18 ×10^{-5}	
5	5.33 ×10^{-5}	5.93 ×10^{-6}	
